# Multi-omic dataset of patient-derived tumor organoids of neuroendocrine neoplasms

**DOI:** 10.1093/gigascience/giae008

**Published:** 2024-03-07

**Authors:** Nicolas Alcala, Catherine Voegele, Lise Mangiante, Alexandra Sexton-Oates, Hans Clevers, Lynnette Fernandez-Cuesta, Talya L Dayton, Matthieu Foll

**Affiliations:** Rare Cancers Genomics Team (RCG), Genomic Epidemiology Branch (GEM), International Agency for Research on Cancer/World Health Organization (IARC/WHO), Lyon 69008, France; Rare Cancers Genomics Team (RCG), Genomic Epidemiology Branch (GEM), International Agency for Research on Cancer/World Health Organization (IARC/WHO), Lyon 69008, France; Rare Cancers Genomics Team (RCG), Genomic Epidemiology Branch (GEM), International Agency for Research on Cancer/World Health Organization (IARC/WHO), Lyon 69008, France; Department of Medicine, Stanford University School of Medicine, Stanford, CA 94305, USA; Rare Cancers Genomics Team (RCG), Genomic Epidemiology Branch (GEM), International Agency for Research on Cancer/World Health Organization (IARC/WHO), Lyon 69008, France; Hubrecht Institute, Royal Netherlands Academy of Arts and Sciences (KNAW) and UMC Utrecht, 3584 CT Utrecht, The Netherlands; Oncode Institute, Hubrecht Institute, 3584 CT Utrecht, The Netherlands; Rare Cancers Genomics Team (RCG), Genomic Epidemiology Branch (GEM), International Agency for Research on Cancer/World Health Organization (IARC/WHO), Lyon 69008, France; Hubrecht Institute, Royal Netherlands Academy of Arts and Sciences (KNAW) and UMC Utrecht, 3584 CT Utrecht, The Netherlands; Oncode Institute, Hubrecht Institute, 3584 CT Utrecht, The Netherlands; Rare Cancers Genomics Team (RCG), Genomic Epidemiology Branch (GEM), International Agency for Research on Cancer/World Health Organization (IARC/WHO), Lyon 69008, France

**Keywords:** organoid, cancer, neuroendocrine neoplasm, genomics, transcriptomics, quality control

## Abstract

**Background:**

Organoids are 3-dimensional experimental models that summarize the anatomical and functional structure of an organ. Although a promising experimental model for precision medicine, patient-derived tumor organoids (PDTOs) have currently been developed only for a fraction of tumor types.

**Results:**

We have generated the first multi-omic dataset (whole-genome sequencing [WGS] and RNA-sequencing [RNA-seq]) of PDTOs from the rare and understudied pulmonary neuroendocrine tumors (*n* = 12; 6 grade 1, 6 grade 2) and provide data from other rare neuroendocrine neoplasms: small intestine (ileal) neuroendocrine tumors (*n* = 6; 2 grade 1 and 4 grade 2) and large-cell neuroendocrine carcinoma (*n* = 5; 1 pancreatic and 4 pulmonary). This dataset includes a matched sample from the parental sample (primary tumor or metastasis) for a majority of samples (21/23) and longitudinal sampling of the PDTOs (1 to 2 time points), for a total of *n* = 47 RNA-seq and *n* = 33 WGS. We here provide quality control for each technique and the raw and processed data as well as all scripts for genomic analyses to ensure an optimal reuse of the data. In addition, we report gene expression data and somatic small variant calls and describe how they were generated, in particular how we used WGS somatic calls to train a random forest classifier to detect variants in tumor-only RNA-seq. We also report all histopathological images used for medical diagnosis: hematoxylin and eosin–stained slides, brightfield images, and immunohistochemistry images of protein markers of clinical relevance.

**Conclusions:**

This dataset will be critical to future studies relying on this PDTO biobank, such as drug screens for novel therapies and experiments investigating the mechanisms of carcinogenesis in these understudied diseases.

Key points:

- Tumor-derived organoids are revolutionary experimental resources to test biological hypotheses and treatment options.
- We have generated the first multi-omic dataset for neuroendocrine tumor organoids of the lung and for the rare neuroendocrine tumors of the pancreas and small intestine (ileum).

## Data Description

### Context

Organoids are 3-dimensional experimental models that summarize the anatomical and functional structure of an organ [1, 2]. Organoids are revolutionizing fundamental and medical research by allowing us to recapitulate human physiology better than animal models, as well as developmental biology contrary to traditional cell cultures [2]. Patient-derived tumor organoids (PDTOs) have been successfully derived for tumors, providing the experimental tools to model disease progression and the preclinical models for personalized treatment testing [3–5]. Although a promising experimental model, PDTOs have currently been developed only for a fraction of tumor types, focusing on the most frequent cancers and those easiest to culture, leaving rare cancers without appropriate experimental models.

We have recently described one of the very first patient-derived organoid biobanks for the rare and understudied neuroendocrine neoplasms [6]. Neuroendocrine neoplasms are rare tumors that can arise in multiple body sites, predominantly in the lung and gastrointestinal tract [7–9]. Neuroendocrine neoplasms are further classified into neuroendocrine tumors (NETs) and neuroendocrine carcinomas (NECs). NETs are themselves subdivided into grades (ranging from 1 to 2 or 3 depending on the organs), while NECs are subdivided into small cell and large cell (LCNEC). While small cell carcinomas are more common (e.g., 15% of lung tumors), have benefited from more studies, and have dedicated treatment options [10], the best treatment option for LCNEC is still unclear [11], and although most NETs progress slowly and have a good prognosis, a subgroup of tumors metastasize and relapse [12].

We report here the multi-omic dataset (whole-genome sequencing [WGS] and RNA sequencing [RNA-seq]) of the neuroendocrine neoplasm PDTO biobank described in [6] (see Table 1). The dataset contains PDTOs of the lung (*n* = 12; 6 grade 1, 6 grade 2) and small intestine ileum (*n* = 6; 2 grade 1 and 4 grade 2), as well as LCNEC of the lung (*n* = 4) and pancreas (*n* = 1). This dataset includes longitudinal sampling of the organoids (2 to 3 time points) and sequencing of the matched parental tumor for most samples (21/23, either primary tumors or metastases). Along with raw and processed data, we provide quality controls for each technique and scripts to run a complete molecular analysis. We also report hematoxylin and eosin–stained (H&E) slides for parental tumors and organoids, brightfield images of organoids, and immunohistochemistry images of neuroendocrine markers (chromogranin A, synaptophysin, CD56, and proliferation marker Ki67) and the EGFR protein. This unique dataset will provide a reference for future research on the understudied neuroendocrine neoplasms.

**Table 1:** Sample summary

ID	Primary site	Tumor type	WGS	RNA-seq	Normal sample (ID)	Tumor sample (ID)	Organoid passages (IDs)
LCNEC1	Pancreas	LCNEC	Yes	Yes	Blood (PANEC1N)	Primary (PANEC1T)	4 (PANEC1Tp4), 14 (PANEC1Tp14)
LNET2	Lung	NET (G1)	Yes	No	Normal-derived organoid passage 7 (LNET2Np7)	Primary (LNET2T)	12 (LNET2Tp12), normal-derived organoid passage 12 (LNET2Np12)
LCNEC3	Lung	LCNEC	Yes	Yes	Tissue (LCNEC3N*)	Primary (LCNEC3T)	17 (LCNEC3Tp17.2), 24 (LCNEC3Tp24)
LCNEC4	Lung	LCNEC	Yes	Yes	Normal-derived organoid passage 6 (LCNEC4Np6)	Primary (LCNEC4T)	7 (LCNEC4Tp7), 24 (LCNEC4Tp24)
LNET5	Lung	NET (G1)	Yes	Yes	Blood (LNET5N)	Primary (LNET5T)	4 (LNET5Tp4), 7 (LNET5Tp7), 2 (LNET5Tp2.2)^†^
LNET6	Lung	NET (G1)	Yes	Yes	Tissue (LNET6N)	Primary (LNET6T)	1 (LNET6Tp1)
mSINET7	Small intestine (ileum)	NET (G2)	Yes	Yes	Blood (SINET7N)	Mesenteric metastasis (SINET7M)	2 (SINET7Mp2)
mSINET8	Small intestine (ileum)	NET (G2)	Yes	Yes	Blood (SINET8N)	Ovary metastasis (SINET8M)	2 (SINET8Mp2)
mSINET9	Small intestine (ileum)	NET (G2)	Yes	No	Blood (SINET9N)	Mesenteric metastasis (SINET9M)	1 (SINET9Tp1)
LNET10	Lung	NET (G2)	Yes	Yes	Blood (LNET10N)	Primary (LNET10T)	4 (LNET10Tp4)
mLCNEC11	Lung	LCNEC	No	Yes	None	Brain metastasis (LCNEC11M)	3 (LCNEC11Mp3)
mSINET12	Small intestine (ileum)	NET (G2)	No	Yes	None	Mesenteric metastasis (SINET12M)	1 (SINET12Mp1 and SINET12Mp1.3)^‡^
LNET13	Lung	NET (G1)	No	Yes	None	Primary (LNET13T)	1 (LNET13Tp1)
LNET14	Lung	NET (G1)	No	Yes	None	Primary (LNET14T)	1 (LNET14Tp1)
mLNET15	Lung	NET (G2)	No	Yes	None	Skin/soft tissue metastasis (LNET15M)	2 (LNET15Mp2)
LNET16	Lung	NET (G2)	No	Yes	None	Primary (LNET16T)	2 (LNET16Tp2)
mLNET16	Lung	NET (G2)	No	Yes	None	Metastasis to the ribcage (LNET16M)	1 (LNET16Mp1)
LNET18	Lung	NET (G2)	No	Yes	None	None	2 (LNET18Tp2, from primary)
LNET19	Lung	NET (G1)	No	Yes	None	Primary (LNET19T)	2 (LNET19Tp2)
mLNET20	Lung	NET (G2)	No	Yes	None	Paravertebral Th1 metastasis (LNET20M)	2 (LNET20Mp2)
mSINET21	Small intestine (ileum)	NET (G1)	No	Yes	None	Paravertebral Th1 metastasis (SINET21M)	2 (SINET21Mp2)
mSINET22	Lung	NET (G1)	No	Yes	None	Paravertebral Th1 metastasis (SINET22M)	2 (SINET22Mp2)
mLCNEC23	Unknown	LCNEC	No	Yes	None	None	3 (LCNEC23Mp3, from paravertebral Th1 metastasis)

For the normal samples, only WGS was performed.

* One normal tissue for this experiment was excluded due to discordance with the tumor (see Fig. 4).

† Two lines were derived for LNET5, one sequenced at passages 4 and 7 (samples LNET5Tp4 and LNET5Tp7) and one at passage 2 (LNET5Tp2.2).

‡ Two lines were derived for SINET12, each sequenced at passage 1 (samples SINET12Mp1.1 and SINET12Mp1.3).

### Methods

#### Sample collection

PDTO lines of the biobank described in [6] were established from surgical resections or biopsies, put in culture, and expanded. PDTOs periodically underwent passaging, a process by which organoids are subcultured to allow future growth [13]; passage time varied from a week to several months depending on the growth rate ([6], Fig. 2). H&E stainings were performed and samples underwent an independent pathological review, and immunohistochemistry of common neuroendocrine markers (chromogranin A, synaptophysin) were performed to confirm the tumoral neuroendrocrine nature of the parental tumors and PDTOs. See [6] for a detailed description of the protocol and the GigaDB repository associated with this article for digital versions of H&E stainings and immunohistochemistry.

**Figure 1:**
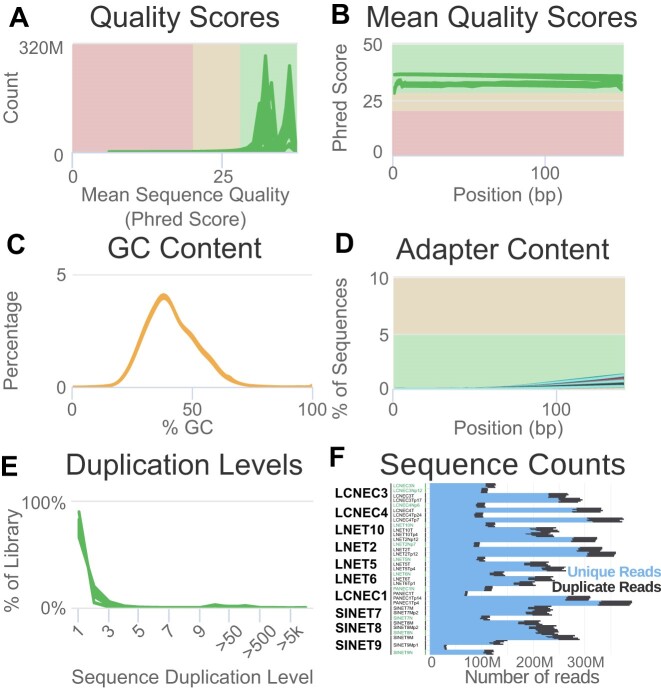
Quality control of the raw WGS data. (A) Distribution of the mean sequence quality of the reads in Phred score. (B) Mean sequence quality score as a function of the position in the read in base pairs (bp). (C) Distribution of the GC content in percentages. (D) Percentage of reads containing a sequence corresponding to the Illumina adapter sequence as a function of the position in the read in bp. (E) Percentage of the library with a given level of duplication. (F) Number of unique and duplicated reads per file. In panels (A–E), each line corresponds to a fastq file, with each of the 34 samples from Table 1 subdivided into 4 sequencing lanes (except SINET9Mp1, subdivided into 8 lanes) and additionally subdivided into 2 read pair files, for a total of 4 × 2 × 33 + 8 × 2 = 280 files; in panel (F), each horizontal bar corresponds to a file. In (A–E), green lines correspond to files that passed the most stringent quality control filters of software FastQC; orange lines correspond to files that passed a less stringent filter.

**Figure 2:**
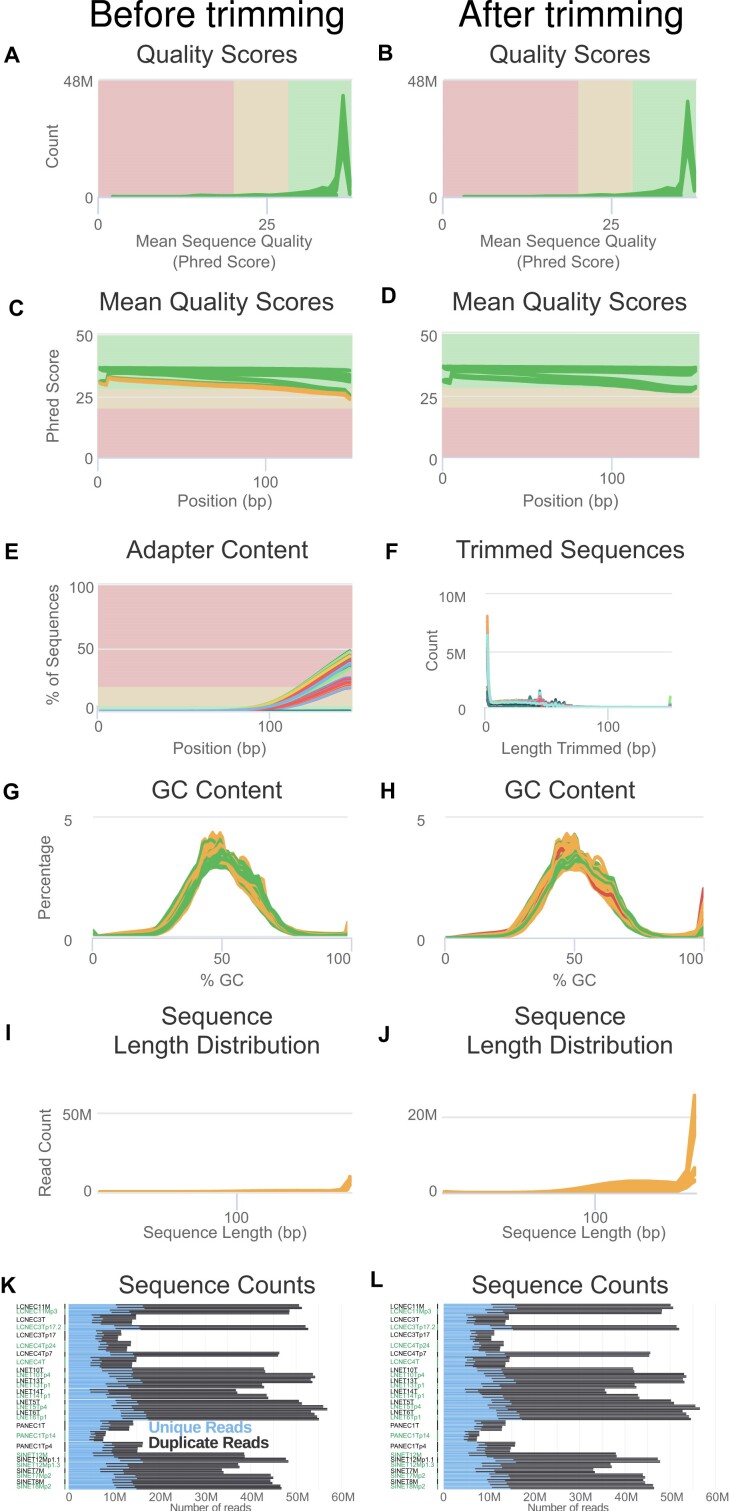
Quality control of the raw RNA-seq data. Panels (A), (C), (E), (G), (I), and (K) correspond to controls before read trimming for quality and adapter content by wrapper Trim Galore for software cutadapt; panels (B), (D), (F), (H), (J), and (L) correspond to controls after read trimming. Figure legends for panels (A–E) and (G–L) follow that of Fig. 1. (F) Distribution of the length of the reads trimmed by software cutadapt, for each file (colored lines). In panels (A–J), each line corresponds to a fastq file, with each of the 10 nonnormal samples from Table 1 divided into 2 or 4 sequencing lanes and further subdivided into 2 read pair files, for a total of 2 × 2 × 21 + 4 × 2 × 7 = 140 files; in panels (K) and (L), each horizontal bar corresponds to a file.

#### Extraction

For each tumor or PDTO, DNA and RNA were extracted from the same sample using the QIAGEN All Prep DNA/RNA Mini kit.

#### Sequencing

##### WGS

Whole-genome sequencing was performed by the Utrecht Sequencing Facility. After DNA quality control, genomic DNA (0.5–1 μg) was used to prepare the whole-genome sequencing library, using the Illumina TruSeq DNA Nano Kit. Libraries were then sequenced on a Novaseq 6000 platform, as paired-end 150-bp reads, with a target average coverage of 30× for normal samples and 60× to 90× for tumor tissue and PDTOs.

##### RNA-seq

RNA sequencing was performed by the Utrecht Sequencing Facility. After RNA quality control, libraries were prepared using the Illumina TruSeq Stranded mRNA polyA Kit. Libraries were sequenced either on a Nextseq 2000 or an Illumina Novaseq 6000 (RRID:SCR_016387), as paired-end 150-bp reads.

#### Data processing

All data processing was performed using the workflows developed by the rare cancers genomics team of the International Agency for Research on Cancer/World Health Organization [14], as detailed in [15] and [16]. The workflows are written in the popular domain-specific language nextflow [17]. All software dependencies are contained in conda environments and containerized with Docker and Singularity (containers available online [18, 19]).

##### WGS

Raw reads were mapped to reference genome GRCh38 using workflow *alignment-nf* v1.2 [20]. This workflow first maps reads (software bwa-mem2 v2.0 [21, 22]), then marks duplicates (software samblaster, v0.1.26 [23]), and finally sorts reads (software sambamba, v0.7.1 [24]).

##### RNA-seq

Raw reads were mapped to reference genome GRCh38 with annotation gencode v33 using the workflow *RNAseq-nf* v2.4 [25]. This workflow removes adapter sequences (wrapper Trim Galore v0.6.5 [26] for software cutadapt [27]), maps reads (software STAR v2.7.3a [28]), marks duplicated reads (software samblaster, v0.1.25), and finally sorts reads (software sambamba, v0.7.1).

Alignments were then postprocessed using 2 workflows to improve their quality. Workflow *abra-nf* v3.0[29] performs local realignment using software ABRA2 (v2.22 [30]), and *BQSR-nf* v1.1[31] performs base quality score recalibration using gatk (v4.0.5.1 [32]).

##### Variant calling from WGS

Single-nucleotide variants were called on all WGS samples using software Mutect2 from GATK4 (v4.2.0.0 [33, 34]) with workflow *mutect-nf* v2.2b[35], as described in [6]. Resulting variant calling format (VCF) files were normalized using bcftools v1.10.2 [36] (workflow vcf_normalization-nf v1.1 [37]) and annotated using ANNOVAR v2020Jun08 (workflow table_annovar-nf v1.1.1 [38]). Indels and multinucleotide variants were additionally filtered using the intersection of Mutect2 and strelka2 [39] calls (workflow strelka2-nf v1.2a[40]), in order to reduce false positives that are more frequent in indel calls due to the difficulty of detecting such variants with short-read sequencing.

##### Variant calling from RNA-seq

Variants were called on all RNA-seq samples using software Mutect2 from GATK4 (v4.2.0.0 [33, 34]) with workflow *mutect-nf* (branch RNAseq)[35] in RNA-seq and tumor-only modes. The RNA-seq mode incorporates a preprocessing step to fix CIGAR strings (removing NDN elements and ensuring that mapping quality 255 is not used as some mappers like STAR can do) and GATK4’s SplitNCigarReads method that splits reads with Ns in their CIGAR string, in order to improve variant calling quality. Resulting VCF files were normalized using bcftools v1.10.2 [36] (workflow vcf_normalization-nf v1.1[37]) and annotated using ANNOVAR v2020Jun08 (workflow table_annovar-nf v1.1.1 [38]). For samples that also had WGS data, RNA-seq–detected variants were classified as somatic or germline based on the WGS variant calls described above.

### Quality control

For each omic technique, quality controls (QCs) of the samples were performed at each step.

#### Raw reads

Software FastQC (v0.11.9 [41]; RRID:SCR_014583) was used to check raw reads quality, and software MultiQC (v1.9 [42]; RRID:SCR_005275) was used to aggregate the QC results across samples and generate interactive plots; all plots from Figs. 1 and 2 were generated by MultiQC from the FastQC outputs. Original MultiQC reports are available in Supplementary Information (Files S1–S4) to allow a free exploration of the QC statistics.

##### WGS

Raw reads passed quality control filters in all samples. All samples displayed good sequence quality scores (mode above 30 Phred, indicating an error rate below 0.2%), both on average and across all positions in the read (Fig. 1A, B), with samples sequenced later (lower part of Table 1, from LNET5 to LNET10) displaying better scores (highest mode in Fig. 1A). GC content was slightly skewed toward lower values but proved consistent across samples (Fig. 1C), and adapter content (Fig. 1D, less than 5% of sequences with adapter sequence detected) and duplication levels (Fig. 1E, less than 20% of sequences present twice or more) were adequate. The number of reads was consistent between read pairs and consistent with target read depths (Fig.   1F): samples with a target depth of 30×—normal, normal-derived organoids, the primary tumor from experiment LCNEC1, and tumor organoid passage 14 from experiment LCNEC3 (LCNEC3Tp14)—having a lower number of reads (∼4 × 100 M reads = 400 M reads) than the others samples (∼4 × 250 M = 1,000 M reads), which had a target depth of 90×. Note that the metastasis organoid of experiment SINET9 (SINET9Mp1) has been sequenced in 8 lanes, with 4 lanes with a low number of reads (∼30 M) and 4 additional ones with a larger number (∼140 M), so the total is comparable with that of the other samples.

##### RNA-seq

Raw reads passed quality filters after reads trimming for adapter content and quality. All samples displayed good sequence quality scores on average both before and after read trimming (mode above 30 Phred; Fig. 2A, B), with samples sequenced later (lower part of Table 1, from LNET5 to LNET14) displaying better scores (highest mode in Fig. 1A). Six samples displayed lower scores at the end of the reads before trimming (Fig. 2C) but better scores after trimming (Fig. 2D). Indeed, most samples displayed high adapter content before trimming (Fig. 2E), and the trimming step successfully removed them (less than 0.1% in all samples; Supplementary Information File S2). The trimming step mostly removed less than 5 bp from the read but occasionally could remove up to around 50 bp (Fig. 2F). GC content was consistent across samples (Fig. 2G, H), although the read-trimming step resulted in an excess of reads with high GC content, presumably due to some reads being strongly shortened by the trimming step. Hopefully, in general, the trimming step did not increase much the proportion of short reads (Fig. 2I, J). The number of reads was consistent between read pairs and across sequencing runs both before and after trimming (Fig. 2K, L), and total read numbers for each sample was consistent with the target number of 50 M (25 M pairs): the smallest number, 60.8 M, corresponded to sample PANEC1Tp14.

#### Alignments

##### WGS

The software qualimap (v2.2.2b [43]; RRID:SCR_001209) was called by our workflow *alignment-nf* to generate QC statistics for the WGS alignments in parallel to the data processing (Table 2). All normal and normal tissue–derived organoids displayed a mean coverage ≥30×, and all tumor and tumor-derived organoids except passage 24 from the organoid of experiment LCNEC4 (sample LCNEC4Tp24) and passage 1 of the organoid of experiment SINET9 (sample SINET9Mp1) had a coverage ≥60×; all samples displayed at least 65% of the genome with a coverage larger than or equal to 30× except LCNEC4Tp24 and (57.4%). Percentages of aligned reads exceeded 99.8% for all samples. Interestingly, some tumor and tumor-derived organoid samples displayed bimodal coverage distributions compatible with variations in copy number state (Supplementary Information File S3).

**Table 2:** Quality control of the WGS alignments

Sample Name	% GC	≥30×	≥50×	Coverage	% Aligned
PANEC1N	42%	84.6%	16.2%	41.0×	99.9%
PANEC1T	42%	93.8%	91.7%	104.0×	99.8%
PANEC1Tp4	42%	93.9%	93.3%	127.0×	99.9%
PANEC1Tp14	42%	93.6%	90.9%	89.0×	99.8%
LNET2Np7	41%	67.2%	1.9%	33.0×	99.9%
LNET2Np12	41%	93.4%	92.9%	104.0×	99.9%
LNET2T	41%	93.3%	92.9%	109.0×	99.9%
LNET2Tp12	42%	93.4%	93.1%	115.0×	99.8%
LCNEC3N	41%	82.5%	11.1%	39.0×	99.9%
LCNEC3Np12	42%	82.5%	11.7%	38.0×	99.9%
LCNEC3T	41%	93.9%	90.0%	89.0×	99.9%
LCNEC3Tp17	42%	93.5%	90.9%	90.0×	99.8%
LCNEC4Np6	42%	69.7%	2.7%	34.0×	99.9%
LCNEC4T	41%	93.0%	88.1%	102.0×	99.8%
LCNEC4Tp7	42%	91.7%	87.9%	102.0×	99.9%
LCNEC4Tp24	42%	51.9%	11.3%	30.0×	99.9%
LNET5N	41%	68.9%	2.5%	33.0×	99.9%
LNET5T	42%	91.9%	77.6%	68.0×	99.9%
LNET5Tp4	42%	93.3%	87.6%	75.0×	99.9%
LNET6N	42%	86.6%	22.8%	43.0×	99.9%
LNET6T	42%	93.0%	83.1%	72.0×	99.9%
LNET6Tp1	42%	90.2%	76.8%	61.0×	99.9%
SINET7N	42%	77.2%	4.9%	36.0×	99.9%
SINET7M	41%	92.8%	83.5%	73.0×	99.9%
SINET7Mp2	42%	92.8%	85.7%	69.0×	99.9%
SINET8N	42%	93.1%	91.7%	75.0×	99.9%
SINET8M	41%	92.6%	81.2%	64.0×	99.9%
SINET8Mp2	42%	93.0%	85.5%	70.0×	99.9%
SINET9N	42%	84.4%	7.2%	38.0×	99.9%
SINET9M	41%	93.0%	90.0%	81.0×	99.9%
SINET9Mp1	42%	90.2%	49.1%	49.0×	99.9%
LNET10N	42%	86.4%	9.5%	39.0×	99.9%
LNET10T	42%	93.0%	90.1%	71.0×	99.9%
LNET10Tp4	42%	93.0%	87.1%	66.0×	99.9%

##### RNA-seq

**Figure 3:**
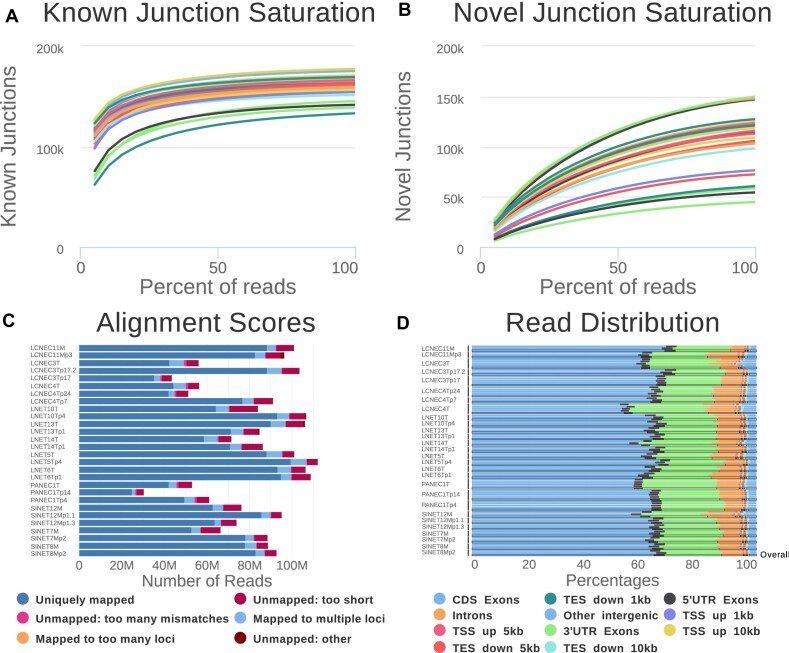
Quality control of the RNA-seq alignments. (A) Number of known junctions identified by software STAR in a subsample as a function of the percentage of reads in the subsample. (B) Number of novel junctions identified by STAR in a subsample as a function of the percentage of reads in the subsample. (C) Number of sequence tags with each alignment score. (D) Distribution of reads among annotated regions.

Software RSeQC (v3.0.1 [44]; RRID:SCR_005275) was called to check alignment quality in parallel to the data processing by workflow *RNAseq-nf*. For all samples, the number of known junctions (i.e., junctions annotated in the gencode v33 annotation file) was stable when resampling subsets of 75% to a 100% of the reads (all lines plateau in Fig. 3A), indicating a good saturation and suggesting that the sequencing depth was sufficient to detect known junctions. In contrast, the number of novel junctions (i.e., junctions not in the annotation file) was increasing slowly as a function of the percentage of reads resampled but did not completely saturate (no complete plateau in Fig. 3B). This indicates that we probably detected the most abundant novel junctions but that some low-abundance novel junctions were probably not detected.

Alignment scores were good, with more than 25 M mapped read pairs (50 M reads) for all samples, and from 4 M to 7 M unmapped reads, mainly due to reads being too short or having too many mismatches (Fig. 3C). The distribution of the alignments within annotated regions matched our expectations, with most reads (≥80%) aligning to exons (≥50%), 3′ UTR (∼20-25%), and 5′ UTR (∼3%) (Fig.   3D).

### Data validation

#### Sample matching

**Figure 4:**
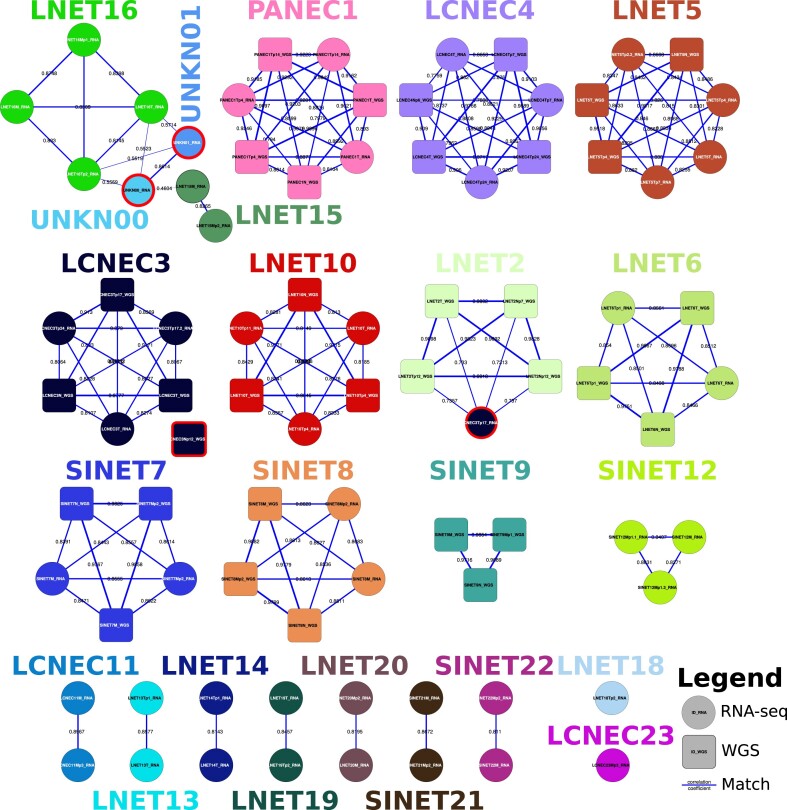
Network of matches between WGS and RNA-seq samples, computed with software NGSCheckmate. Numbers on the edges and edge thickness correspond to the Pearson correlation coefficient *r* between allelic fractions for the germline SNP panel; colors: experiments (see Table 1); squares: WGS, circles: RNA-seq, red contour: mismatches.

We used software NGSCheckMate (cloned from the GitHub repository [45] revision 10799087bdfe4b990add5b5e536f87c47bbdb688; RRID:SCR_022994) to check that samples from the same experiment indeed came from the same individual, in both WGS and RNA-seq simultaneously, using our workflow *NGSCheckMate-nf* v1.1[45]. The sample-matching algorithm correctly identified all experiments except one (Fig. 4). The WGS normal-derived organoid sample from experiment LCNEC3 (LCNEC3Np12_WGS in Fig. 4) was found not to match other LCNEC3 samples, suggesting a possible sample swap, and thus excluded from further analyses. Also, the RNA-seq tumor sample for the late-passage organoid of experiment LCNEC3 (sample LCNEC3Tp17_RNA in Fig. 4) was found to better match experiment LNET2 and thus excluded from the subsequent analyses. Finally, 2 samples were found to partially match LNET15 and LNET16, suggesting contamination, and also excluded (UNKN00 and UNKN01).

#### Sex validation

**Figure 5:**
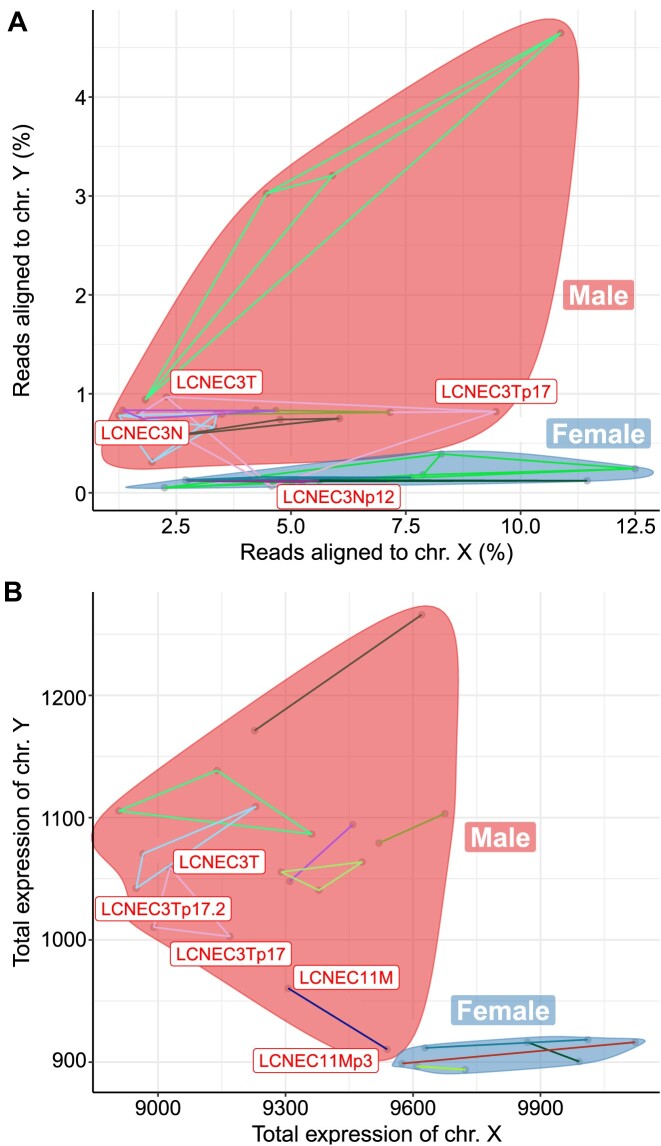
Validation of reported sex. (A) Percentage of reads aligned to chromosomes X and Y in the whole-genome sequencing data. (B) Total gene expression in X and Y chromosomes, in units of variance-stabilized read counts, computed from RNA-seq data. In all panels, samples from each sex are encircled (red: male, blue: female), excluding LCNEC3Np12, which we report as not matching the other samples from the LCNEC3 experiment.

We validated the sex reported in the clinical data using the multi-omic data. For the WGS data, we used the proportion of reads aligned to the sex chromosomes to assess whether samples clustered by sex (Fig. 5A). We found that all samples clustered by sex except for the normal of experiment LCNEC3 (sample LCNEC3Np12), which clustered with females despite other samples from the experiment clearly clustering with males. This further supports the sample matching reports that suggest that this sample does not match the rest of the experiment. For the RNA-seq data, we compared the total expression level on the sex chromosomes, using the variance-stabilized read counts as a quantification of gene expression (vst function from R package DESeq2 v1.26.0 [46]) (Fig. 5B). We find that samples from the same sex cluster together for all experiments, suggesting concordance with the clinical data.

#### Small variant calls from RNA-seq

**Figure 6:**
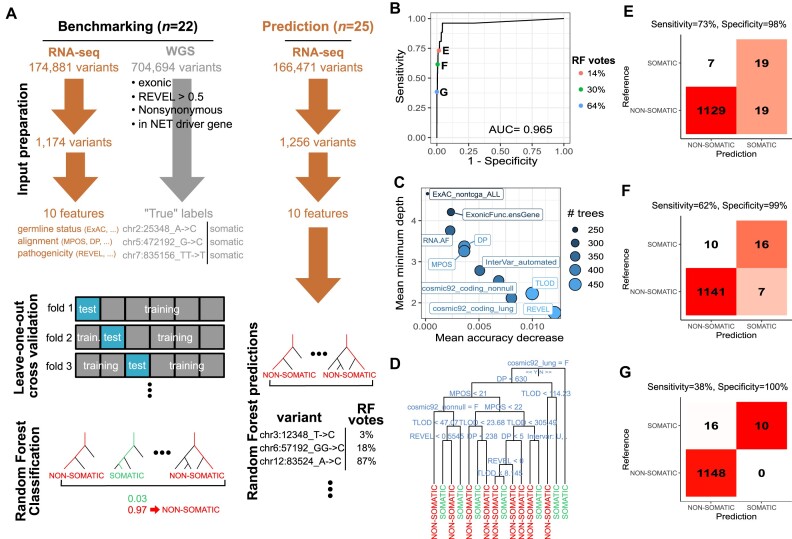
RF classification of variants as somatic or germline from RNA-seq data. (A) Schematic of the RF training, test, and prediction. (B) ROC curve. (C) Feature importance for classification accuracy. Mean accuracy decrease: mean difference in accuracy between trees with the feature and trees without the feature; high values indicate important features. Mean minimum depth: tree depth (1: root, value >>1: leaves) of the first time the feature is used for classification, averaged across all trees; low values indicate features often used at the root and thus particularly important. (D) Representative tree of the RF. At each split, the split condition is written above; the left branch corresponds to a Yes and the right branch to a No. Final decision (SOMATIC or NON-SOMATIC) is represented by the leaves. (E–G) Confusion matrix for different levels of sensitivity and specificity. Reference: somatic status assessed from whole-genome sequencing data. Prediction: somatic status predicted from RNA-seq data using the RF algorithm.

We classified small variants called from RNA-seq in 241 known neuroendocrine neoplasm driver genes (see from Table S4 from reference [6]) as somatic or germline, using a random forest (RF) algorithm [47] (R package randomForest v4.7-1.1 [48]; Fig. 6) and a similar approach as we recently did to classify mutations in tumor-only WGS [49]. After filtering out nonexonic, synonymous, and nonsynonymous mutations with a REVEL score [50] below 0.5, and mutations not in the list of 241 drivers, we were left with 2,430 variants. Among them, 1,174 variants were in samples with WGS data available and their somatic status was thus known.

We used 10 features in the RF model. One feature was directly informative about the potential germline status and came from a public database: the frequency of the allele in human populations from the ExAc database excluding cancers from The Cancer Genome Atlas (TCGA )(feature *ExAC_nontcga_ALL*). Four features were informative about the alignment and came from the sequencing data themselves: the median distance from the end of the read (feature *MPOS*), the likelihood ratio score of variant existence (feature *TLOD*), the coverage at the position (feature *DP*), and the allelic fraction of the alternative allele (RNA.AF). Finally, the other features were informative about the pathogenicity of the variant and came from public databases: the REVEL score of pathogenicity (feature *REVEL*), the presence in the COSMIC 92 database (feature *cosmic*92_*coding_nonnull*), the presence in the COSMIC 92 database in a lung tumor (feature *cosmic*92_*coding_lung*), the InterVar annotation (feature *InterVar_automated*; with levels “.,” “Uncertain_significance,” “likely_pathogenic,” and “Pathogenic”), and the exonic function of the variant (missense, nonsense, inframe or frameshift insertion, etc).

The RF algorithm was trained and tested on the 1,174 variants with known status (1,148 germline, 26 somatic) called in 22 samples from 8 experiments (Fig. 6A). Note that although the data are imbalanced, we chose to keep this imbalance in the training set to force the algorithm to take into account the fact that most variants are not somatic, and thus having a very good specificity is key to avoid large false discovery rates. We used leave-one-out cross-validation at the experiment level (8 folds), excluding all samples from one same experiment from the model fit at each iteration in order to avoid overfitting due to the inclusion of variants from the same individual but different samples (e.g., LCNEC3T and LCNEC3Tp17) in the training and test sets. We used 5,000 trees and 3 features per split (the square root of the total number of features as recommended by default) and a minimal node size of 1. We estimated the performance of the model using the receiver operating characteristic (ROC) curve and its area under the curve (AUC, computed using the trapezoid rule), showing the sensitivity as a function of 1 − specificity across different thresholds for the proportion of votes for the somatic class. We also computed the false discovery rate to get a sense of the proportion of variants classified as somatic that would actually be false positives. Once the RF model performance was assessed, we trained a RF model on the full 1,174 variants and predicted the status of the remaining 1,256 variants. See the GitHub repository associated with the article for the complete R script [51]. Note that the same approach allowed us to classify variants called from tumor-only WGS data as somatic or germline with high performance (accuracy greater than 92%; [49]).

We find that we can classify variants as somatic or germline with a balanced accuracy of 86%, with both specificity greater than 98% and sensitivity greater than 73% (AUC = 0.965). Interestingly, although somatic variants are just a fraction of the calls (2%), the high sensitivities and specificities of our RF algorithm allowed us to classify variants with false discovery rates below 50% while still preserving sensitivities above 60% (see Fig. 6B, E–G). We also tested the predictive accuracy of the model fitted on this set of 1,174 variants from known neuroendocrine neoplasm genes on the set of somatic variants from other recurrently mutated genes in our cohort (Supplementary Fig. S1). We find that the predictive power of the RF model was similar (AUC = 0.90, sensitivity up to 73% with a specificity above 87%).

We evaluated the importance of features for the classification using both the mean decrease in accuracy, which captures how much the model loses accuracy when the feature is excluded, and the mean tree depth at which the feature was observed, with a low value meaning that the feature is used early in the decision trees and thus separates many variants [47, 52] (R package randomForestExplainer v0.10.1). The most important features for the classification were the REVEL score, the TLOD, and the cosmic annotation, while the frequency in the ExAC database was the least important, presumably because all these variants were very rare (Fig. 6C). Indeed, the most representative tree from the RF, computed using the reprtree R package v0.6 using the d2 distance metric between tree predictions [53], relied on these 3 variables, with all alterations present in a lung tumor from the COSMIC 92 database automatically classified as somatic (root of the tree), and TLOD and REVEL score being the most common features used for splitting (Fig. 6D). Of note, using the most important feature alone (the REVEL score) led to a much lower accuracy, consistent with the importance of other features such as TLOD and pathogenic annotations (COSMIC, InterVar).

#### Comparing molecular profiles of PDTOs and parental tumors

We report here all the R scripts used in Dayton et al. [6] to validate that PDTOs faithfully represent their parental tumors (available on the GitHub repository associated with the article [51]). In particular, we provide the code that we used to compare the expression profiles of PDTOs and reference lung and small intestine (SI) NETs and LCNECs with that of PDTOs and their parental tumors (file Fig3B_S3BCE.md [51]). This analysis confirmed the neuroendocrine nature of the PDTOs by showing that they express neuroendocrine markers routinely used in the clinic (>1 transcript per million, TPM in at least 1 of 6 markers). We also provide the code (file Fig3CD_S3FGHI.md [51]) used in Dayton et al. [6] to demonstrate that pure PDTOs preserve the expression profiles of their parental tumor using dimensionality reduction techniques (Uniform Manifold Approximation and Projection, UMAP). In addition, we provide the code (files Fig4BC_S4BC.md and Fig4D_S4D.md [51]) used to show that PDTOs preserve the genomic profile (small variants, copy number variants, and structural variants) of their parental tumor. To do so, we focused on mutations known to be drivers of neuroendocrine neoplasms [15, 54–60]. Both variants identified with WGS and variants identified with RNA-seq include driver mutations in key recurrently altered LCNEC driver genes such as *TP53* (mutated in 5/5 LCNECs) and *STK11* (mutated in 3/5 LCNECs). We also identified mutations or structural variants in known driver genes in all but 1 neuroendocrine tumors (17/18), but as previously reported, they involve multiple genes instead of recurrently mutated genes [15, 61]. This confirms that PDTOs recapitulate the genomic profile of neuroendocrine neoplasms.

We also report the R scripts used in Dayton et al. [6] to analyze the temporal evolution of the PDTOs (file Fig5_S5.md [51]). These analyses showed that PDTOs preserve the genetic diversity and clonal architecture of their parents across long periods of time (6 months to more than a year). In particular, the analysis of 2 samples with multiple time points (LCNEC1 and LCNEC4) highlighted that the genetic makeup of the parental tumor is preserved across PDTO passages.

Of note, 1 sample, LCNEC23 was a paravertebral metastasis of an LCNEC of unknown primary. As mentioned in Dayton et al. [6] (fig. 3), the transcriptome of this sample did cluster with the other LCNEC from the lung and pancreas; in addition, we detected from the RNA-seq 2 high-confidence somatic mutations characteristic of LCNEC: a nonsynonymous *TP53* and a nonsense *PIK3CA* mutation. These molecular results comfort the LCNEC nature of the PDTO, but the overlap between known lung and pancreas LCNEC profiles does not allow to infer the site of origin of the tumor.

### Reuse potential

We describe here some of the very first multi-omic datasets for patient-derived tumor organoids of pancreatic, small intestine (ileum), and pulmonary neuroendocrine neoplasms, in particular including the first lung neuroendocrine tumor organoids. Because such low-grade tumors are difficult to cultivate *in vitro*, there is currently a lack of adequate experimental systems for these tumors, and we expect the biobank associated with the data presented here to be the basis for future experimental studies—either fundamental or treatment oriented—on neuroendocrine neoplasms across body sites. The multi-omic dataset we provide here constitutes the molecular fingerprints of these experimental models and will be key to investigate oncogenic processes responsible for tumor initiation and progression and to link drug responses to molecular features to design future personalized treatments.

To facilitate future studies, we used the exact same data processing as in our previous studies of neuroendocrine neoplasms [15, 16] and other rare cancers [62], in particular using rigorous RNA-seq expression quantification with containerized software and operating systems (see Methods section). To ease future studies, we make the expression matrix publicly available (file gene_expression_PDTOs_parents.tsv in reference [51]). In addition, we provide all R scripts to analyze the data [51].

Note that the slow passage time of low-grade PDTOs makes them appropriate models to study the biology of neuroendocrine tumors but challenges their use for drug testing. This is particularly true of small intestine NETs, which were only short-term cultures that did not grow past 4 passages. Finally, as noted in most molecular studies of PDTOs [63], one of the main differences between PDTOs and their parental tumors is the absence of a microenvironment. Future work would ideally focus on creating cocultures of PDTOs and immune cells to remedy this shortcoming.

## Conclusion

We have shown that our multi-omic dataset is of high quality and can be easily reused. Given the rarity of neuroendocrine tumors from the lung, pancreas, and small intestine, past genomic studies each only reported data for a handful of samples, limiting the potential discoveries. For example, for lung NETs, 29 WGS and 39 RNA-seq were reported in [61], 3 WGS and 20 RNA-seq in [15], and 30 RNA-seq in [64]; for small intestine NETs, for example, 81 RNA-seq with no WGS were reported in [65] and 7 RNA-seq in [66]. As a result, the primary tumors and metastasis sequencing data we report here (10 samples with WGS, 21 with RNA-seq) alone are very valuable and should be combined with other datasets in future studies to provide enough power to discover informative molecular features for diagnosis, prognosis, and treatment. In addition, we report a unique multi-omic dataset generated from patient-derived tumor organoids, which will allow all researchers working on our biobank to test hypotheses regarding the molecular features associated with drug responses and thus advance research on personalized treatments for these understudied diseases.

## Availability of Source Code and Requirements

- Project name: NEN organoids project, lungNENomics
- Project homepages: https://www.embl.org/groups/dayton/, http://rarecancersgenomics.com/lungnenomics/
- Operating system(s): Platform independent
- Programming language: Nextflow, R
- Other requirements: R packages *caret, randomForest*
- License: GNU GPL

All nextflow command lines for data processing are available at [51] in the readme. All R scripts for the analysis are available in the subfolder Rscripts [51].

## Additional Files

**Supplementary Fig. S1**. Random forest (RF) classification of variants in genes not reported as driver in neuroendocrine neoplasms. (A) ROC curve. (B–D) Confusion matrix for different levels of sensitivity and specificity. Reference: somatic status assessed from whole-genome sequencing data. Prediction: somatic status predicted from RNA-seq data using the RF algorithm.

**Supplementary File 1**. MultiQC report for raw whole-genome-sequencing data.

**Supplementary File 2**. MultiQC report for raw RNA-sequencing data.

**Supplementary File 3**. MultiQC report for whole-genome-sequencing alignments.

**Supplementary File 4**. MultiQC report for RNA-sequencing alignments.

## Supplementary Material

giae008_SupplementaryFileS1

giae008_SupplementaryFileS2

giae008_SupplementaryFileS3

giae008_SupplementaryFileS4

giae008_Authors_ReResponse_to_Reviewer_Comments_Revision_1sponse_To_Reviewer_Comments

giae008_GIGA-D-23-00277_Original_Submission

giae008_GIGA-D-23-00277_Revision_1

giae008_GIGA-D-23-00277_Revision_2

giae008_Response_to_Reviewer_Comments_Original_Submission

giae008_Reviewer_1_Report_Original_Submission Masashi Fujita -- 10/29/2023

giae008_Reviewer_2_Report_Original_Submission Saurabh V Laddha -- 11/9/2023

giae008_Reviewer_3_Report_Original_Submission Qiuyue Yuan -- 11/15/2023

## Data Availability

The dataset supporting the results of this article is available in the European Genome-Phenome archive repository, study EGAS00001005752. The study consists of 7 datasets: EGAD00001009988, with WGS CRAM files for 2 experiments; EGAD00001009989 with WGS CRAM files for 6 experiments; EGAD00001009990, with WGS CRAM files for 2 experiments; EGAD00001009991, with RNA-seq fastq files from 4 experiments; EGAD00001009992, with RNA-seq fastq files for 15 experiments; EGAD00001009993, with RNA-seq fastq files for 2 experiments; and EGAD00001009994, with gene expression in multiple formats (R data, tab-separated text files) and multiple units (raw counts, TPM, FPKM) for 21 samples. Because of the sensitivity of the data and the patient consent, to get access to the data, please contact the data access committee of the Division of Biomedical Genetics from UMC Utrecht at dacdbg@umcutrecht.nl. Once a data access agreement has been signed and access granted, data can be downloaded using the EGA Python client (see detailed instructions [67], and video tutorial [68]). Expression matrices in raw counts format and small variants are also publicly available on the GitHub repository under the data folder [51]. The multiQC report for WGS raw reads is available in Supplementary File S1, the multiQC report for RNA-seq raw reads is available in Supplementary File S2, the multiQC report for WGS alignments is available in Supplementary File S3, and the multiQC report for RNA-seq alignments is available in Supplementary File S4. Snapshots of our code and other data further supporting this work are openly available in the *GigaScience* repository, GigaDB [69]. Organoid lines mentioned in this article can be requested from Hans Clevers (h.clevers@hubrecht.eu) or Talya Dayton (talya.dayton@embl.es). Distribution of organoids to third parties will have to be authorized by the relevant ethical committee, and a complete material transfer agreement will be required to ensure compliance with the Dutch “Medical Research Involving Human Subjects” act. Use of organoids is subjected to patient consent; note that upon consent withdrawal, distributed organoid lines and any derived material will have to be promptly disposed of.

## Abbreviations

AUC: area under the curve; bp: base pairs; LCNEC: large-cell neuroendocrine carcinoma; NEC: neuroendocrine carcinoma; NET: neuroendocrine tumor; PDTO: patient-derived tumor organoid; QC: quality control; RF: random forest; RNA-seq: RNA sequencing; ROC: receiver operating characteristic; VCF: variant calling format; WGS: whole-genome sequencing.
