# Supplementary material for: Multi-omic dataset of patient-derived tumor organoids of neuroendocrine neoplasms
Source: Gigascience. 2024 Mar 7;13:giae008. doi: 10.1093/gigascience/giae008 (PMC10919335; doi:10.1093/gigascience/giae008)
Supplement: giae008_GIGA-D-23-00277_Revision_1 [file giae008_giga-d-23-00277_revision_1.pdf]

GigaScience

Multi-omic dataset of patient-derived tumor organoids of neuroendocrine neoplasms

--Manuscript Draft--

**Manuscript Number:**	GIGA-D-23-00277R1
**Full Title:**	Multi-omic dataset of patient-derived tumor organoids of neuroendocrine neoplasms
**Article Type:**	Data Note

Funding Information:

Neuroendocrine Tumor Research Foundation	Dr Hans Clevers<br>Dr Lynnette Fernandez-Cuesta
Worldwide Cancer Research	Dr Lynnette Fernandez-Cuesta
Institut National Du Cancer	Dr Lynnette Fernandez-Cuesta<br>Dr Matthieu Foll
Ligue Contre le Cancer	Dr Lise Mangiante
European Molecular Biology Organization	Dr Talya L. Dayton
H2020 Marie Skłodowska-Curie Actions (797966)	Dr Talya L. Dayton
KWF Kankerbestrijding	Not applicable

Abstract:

Background: Organoids are three-dimensional experimental models that summarize the anatomical and functional structure of an organ. Although a promising experimental model for precision medicine, patient-derived tumor organoids (PDTOs) have currently been developed only for a fraction of tumor types.
Results: We have generated the first multi-omic dataset (whole-genome sequencing, WGS, and RNA-sequencing, RNA-seq) of PDTOs from the rare and understudied pulmonary neuroendocrine tumors (n=12; 6 grade 1, 6 grade 2), and provide data from other rare neuroendocrine neoplasms: small intestine (ileal) neuroendocrine tumors (n=6; 2 grade 1 and 4 grade 2) and large-cell neuroendocrine carcinoma (n=5; 1 pancreatic and 4 pulmonary). This dataset includes a matched sample from the parental sample (primary tumor or metastasis) for a majority of samples (21/23) and longitudinal sampling of the PDTOs (1 to 2 time-points), for a total of n=47 RNA-seq and n=33 WGS. We here provide quality control for each technique, and provide the raw and processed data as well as all scripts for genomic analyses to ensure an optimal re-use of the data. In addition, we report gene expression data and somatic small variant calls and describe how they were generated, in particular how we used WGS somatic calls to train a random-forest classifier to detect variants in tumor-only RNA-seq. We also report all histopathological images used for medical diagnosis: hematoxylin and eosin-stained slides, brightfield images, and immunohistochemistry images of protein markers of clinical relevance.
Conclusions: This dataset will be critical to future studies relying on this PDTO biobank, such as drug screens for novel therapies and experiments investigating the mechanisms of carcinogenesis in these understudied diseases.

**Corresponding Author:**	Nicolas Alcala<br>International Agency for Research on Cancer<br>Lyon, Rhône-Alpes FRANCE
**Corresponding Author Secondary Information:**	
**Corresponding Author's Institution:**	International Agency for Research on Cancer
**Corresponding Author's Secondary Institution:**	
**First Author:**	Nicolas Alcala
**First Author Secondary Information:**	
**Order of Authors:**	Nicolas Alcala
	Catherine Voegele
	Lise Mangiante

	Alexandra Sexton-Oates
	Hans Clevers
	Lynnette Fernandez-Cuesta
	Talya L. Dayton
	Matthieu Foll
**Order of Authors Secondary Information:**	
**Response to Reviewers:**	Dear Dr Alcala,<br><br>Your manuscript "Multi-omic dataset of patient-derived tumor organoids of neuroendocrine neoplasms" (GIGA-D-23-00277) has been assessed by our reviewers. Based on these reports, and my own assessment as Editor, I am pleased to inform you that it is potentially acceptable for publication in GigaScience, once you have carried out some essential revisions suggested by our reviewers. In particular, Reviewer #3 would like the detailed data processing steps to be added to the GitHub page, for ease of reuse and reproducibility.<br>Answer: Dear Dr Nogoy, thank you very much for your positive feedback and for the possibility to submit a revised version. Please find a detailed answer to all comments below. In particular, we have added the requested details about data processing on the github page, now providing the exact command lines used for each data processing step as well as all the parameters used. Also note that we opted at submission to also submit to GigaDB all H&E section images and Immunohistochemistry section images of markers we generated, so we now also report them in this revised version of the manuscript. In the revised manuscript, we highlight in red important modifications.<br><br><br>Reviewer reports<br>Reviewer #1<br><br>In this manuscript, Alcala et al. have reported on the whole genome sequencing (WGS) and RNA sequencing (RNA-seq) of 23 patient-derived tumor organoids of neuroendocrine neoplasms. This is a detailed report on the quality control of WGS, RNA-seq, and sample swap. The methods are solid and well-described. The raw sequencing data have been deposited in a public repository. This dataset could be a valuable resource for exploring the biology and treatment of this rare type of tumor.<br>Answer: We thank the reviewer for their positive assessment of our work.<br><br><br>Here are my comments to the authors:<br>Comment 1. Could you please clarify whether the organoids described in this manuscript will be distributed? If so, could you provide the contact address and any restrictions, such as a material transfer agreement?<br>Answer: We thank the reviewer for pointing out this oversight. We now mention the contact address and procedure to obtain the organoids p. 10: “Organoid lines mentioned in this manuscript can be requested from Hans Clevers (h.clevers@hubrecht.eu) or Talya Dayton (talya.dayton@embl.es). Distribution of organoids to third parties will have to be authorized by the relevant ethical committee and a complete material transfer agreement will be required to ensure compliance with the Dutch ‘medical research involving human subjects’ act. Use of organoids is subjected to patient consent; note that upon consent withdrawal, distributed organoid lines and any derived material will have to be promptly disposed of.”<br><br>Comment 2. You have deposited the RNA-seq gene expression matrix in the public repository European Genome-phenome Archive (dataset ID: EGAD00001009994). However, the file is under controlled access. This limits the availability of data, especially for scientists who just want a quick glance at the data. Since the gene expression matrix does not contain personally identifiable information, I wonder if you could make the file open access. You have reported how you detected somatic mutations in the organoids. However, you did not share the list of detected mutations. Sharing this list would help scientists who do not have a computational background. Open access is preferable in this case, but controlled access is also acceptable because germline variants could be misclassified as somatic.

Powered by Editorial Manager® and ProduXion Manager® from Aries Systems Corporation

Answer: The reviewer makes a valid point; we now provide the gene expression matrix and the small variant alterations in the public github repository associated with the manuscript (https://github.com/IARCbioinfo/MS_panNEN_organoids) and provide a permanent link to their location p. 10.

Comment 3. The primary site of mLCNEC23 is unknown. Could you infer its primary site based on gene expression patterns or driver mutations?
Answer: We now mention p. 8 that molecular data of mLCNEC23 did support its LCNEC nature---clustering with other LCNEC and presence of mutations characteristic of LCNEC such as in gene TP53---but that since LCNEC from multiple organs clustered together and had similar driver mutations, the organ of origin could not be determined based on molecular data.

Comment 4. I have concerns about the generalizability of your random forest model because it was trained using only 22 somatic mutations. Could you assess your prediction model using publicly available datasets of cancer genomes (e.g., TCGA)?
Answer: We now provide additional elements to support the generalization of our model pp. 7-8. We show in a new Figure S1 that the random forest model trained on these 22 somatic mutations from known neuroendocrine neoplasm cancer genes produces similar accuracy when tested on the 223 somatic and 395 non-somatic mutations from all other recurrently mutated genes from the cohort (AUC=0.90, sensitivity up to 73% with a specificity above 87%). We also mention that we have used a similar approach in the past and validated it on WGS tumor-only samples, which allowed to "classify variants called from tumor-only WGS data as somatic or germline with high performance (accuracy greater than 92%; di Genova et al. GigaScience 2023)".

Reviewer #2

Alcala et al., did an excellent work on rare cancer type by creating PDTOs molecular fingerprint which has a direct impact for researcher working on these rare cancer type. As a data note, this is excellent resource and covering huge gap in this rare cancer field.

These PDTOs holds high impact specially for such cancers which are slow growing and not easy culture in lab. Authors covered details regarding each technique used in this study and figures are clear to understand with exceptional writing.
Answer: We thank the reviewer for their positive assessment of our work.

Minor comments:
Comment 1. Did authors compare the PDTOs to tumor molecular dataset ? This will be the key to understand how closely and qualitatively PTDOs are related to actual tumor datasets molecular profile. It is not clear in the current version and it will be helpful to readers to decide whether PTDOs molecular fingerprint system are valuable to them. This is not required for this manuscript to address but a note will be helpful to make valulabe decision to use such resources and with what limitations.
Answer: We agree with the reviewer that although this is beyond the scope of such a data note paper, the comparison of PDTOs and parental tumors is key. Thus, we have added on p. 8 a summary of the results from the associated research article (Dayton, Alcala et al. In press), where this comparison is thoroughly investigated. In addition, in this data note we report all the scripts used to perform the thorough comparison between PDTOs and parental tumors presented in the associated research article. In particular, we mention the location of the scripts showing that the organoids faithfully represent the gene expression profile and genomic profile of their parental tumors (freely available at https://github.com/IARCbioinfo/MS_panNEN_organoids/tree/main/Rscripts).

Comment 2. Authors covered longitudinal samples in this system for 1 to 2 timepoints. What changes did they observe (molecularly) looking at this data from a longitudinal timepoints view will be helpful for readers. Also, based on author's experience for longitudinal sampling, do authors have key suggestions for researcher ? a brief discussion will be helpful.
Answer: This is also a key aspect covered in the associated research article. We now

Powered by Editorial Manager® and ProduXion Manager® from Aries Systems Corporation

summarize in this data note p. 8 the main results from the associated research article, in particular mentioning that " PDTOs preserve the genetic diversity and clonal architecture of their parents across long periods of time (6 months to more than a year)". We also report all the scripts for the longitudinal evolutionary analyses of the samples, so researchers can use it as inspiration for their own studies.

Comment 3. Authors did comprehensive small variant analysis from WGS and RNAseq. Did you authors find known somatic variations for these samples ? mainly comparing against the known published mutational landscape. A note of this will be helpful.
Answer: We now mention p. 8 that "both variants identified with WGS and variants identified with RNA-seq include driver mutations in key recurrently altered LCNEC driver genes such as TP53 (mutated in 5/5 LCNEC) and STK11 (mutated in 3/5 LCNEC). We also identified mutations or structural variants in known driver genes in all but one neuroendocrine tumors (17/18), but as previously reported, they involve multiple genes instead of recurrently mutated genes (Fernandez-Cuesta et al. 2014). This confirms that PDTOs recapitulate the genomic profile of neuroendocrine neoplasms". We also now mention that we report all the scripts necessary to perform these analyses p. 8.

Comment 4. A comment on limitations of PTDOs and molecular fingerprint created from such PDTOs will be valuable.
Answer: We now mention in the re-use potential section p. 9 the limitations of this PDTO biobank, notably "that the slow passage time of low-grade PDTOs makes them appropriate models to study the biology of neuroendocrine tumors, but challenges their use for drug testing. This is particularly true of small intestine NETs, which were only short term cultures that did not grow past four passage. Finally, as noted in most molecular studies of PDTOs (Lee et al Cell 2018), one of the main differences between PDTOs and their parental tumors is the absence of microenvironment. Future work would ideally focus on creating co-cultures of PDTOs and immune cells to remedy this shortcoming".

Comment 5. Authors briefly comment on using such molecular datasets from PDTOs and combining with other datasets to improve on power statistics to discover informative molecular features of these cancers. This points towards my first point on how similar PDTOs are to tumor molecular profile.
Answer: We now provide in the github repository the expression matrix processed exactly as our past (Gabriel et al. Gigascience 2020) and future studies (Sexton-Oates et al. In prep), and mention its public location in the re-use potential section. We refer the reviewer to the answer to comment 1 for details about similarities with reference tumors.

Reviewer #3

The authors conducted a study where they generated multi-omics datasets, including whole-genome sequencing and RNA sequencing , for rare neuroendocrine tumors in the lungs, small intestine, and large cells. They used patient-derived tumor organoids and performed quality control analysis on the datasets. Additionally, they developed a random forest classifier specifically for detecting mutations in the RNA-seq data. The pipeline used in this study is well-organized, but I have a few queries that I would like to clarify before recommending it for publication.

Major concerns:
Comment 1. The data processing and quality control procedures would be valuable for other researchers working with similar datasets. It would be beneficial to add these procedures to the GitHub repository (https://github.com/IARCbioinfo/MS_panNEN_organoids). Furthermore, it would be helpful to provide insights into what constitutes good quality reads, such as the number of unique reads and the ratio of duplicate reads.
Answer: We thank the reviewer for noting this oversight in our code availability. We have now added all the command lines used for data processing the RNA-seq and WGS in the github repository (see readme https://github.com/IARCbioinfo/MS_panNEN_organoids) and mention them in the code

availability section p. 10. Note that we also provide the processed data per the other reviewers' suggestions (gene expression and small variants, see answer to comment 2 from reviewer 1). Finally, we now also mention the general guidelines from software fastQC regarding the quality of reads p. 4.

Comment 2. Regarding the random forest (RF) model, it is mentioned that there are 10 features. Could you clarify if these features are from the public information, or are all the features extracted solely from the RNA-seq data? Also, does the RF model work for WGS data as well? Was there any specific design implemented to address the issue of imbalanced positive and negative samples?
Answer: We now explicitly mention p. 6 which of the 10 features come from the RNA-seq data (4/10 features) and which are annotations coming from public databases (6/10 features). We also mention p. 7 that indeed a similar model was used in Di Genova et al. on WGS data to classify variants from tumor-only samples. Regarding the issue of imbalanced positive and negative samples, we now mention pp. 6-7 that we preferred to keep this imbalance in the training set to force the algorithm to take into account the fact that most variants are not somatic, and thus having a very good specificity is key to avoid large false discovery rates.

Comment 3. RNA-seq are not used to generate the gene expression here, which would waste important information.
Answer: We now make it explicit that we did generate gene expression data and that it is available on the github and EGA repositories (see revised data availability section p. 9).

Minor concerns:
Comment 4. In Figure 6C, what does "Mean minimum depth" refer to?
Answer: We now describe in the figure legend that "Mean minimum depth" refers to the "tree depth (1: root, value $>>1$: leaves) of the first time the feature is used for classification, averaged across all trees; low values indicate features often used at the root and thus particularly important".

Comment 5. Is the most important feature identified by the RF model a good predictor?
Answer: We now mention p. 8 that while this feature is particularly important, "using the most important feature alone (the REVEL score) led to a much lower accuracy, consistent with the importance of other features such as TLOD and pathogenic annotations (COSMIC, InterVar)".

Additional Information:	
**Question**	**Response**
Are you submitting this manuscript to a special series or article collection?	No
**Experimental design and statistics**<br><br>Full details of the experimental design and statistical methods used should be given in the Methods section, as detailed in our Minimum Standards Reporting Checklist. Information essential to interpreting the data presented should be made available in the figure legends.<br><br>Have you included all the information requested in your manuscript?	Yes
**Resources**	Yes

Powered by Editorial Manager® and ProduXion Manager® from Aries Systems Corporation

A description of all resources used, including antibodies, cell lines, animals and software tools, with enough information to allow them to be uniquely identified, should be included in the Methods section. Authors are strongly encouraged to cite Research Resource Identifiers (RRIDs) for antibodies, model organisms and tools, where possible.<br><br>Have you included the information requested as detailed in our Minimum Standards Reporting Checklist?	
**Availability of data and materials**<br><br>All datasets and code on which the conclusions of the paper rely must be either included in your submission or deposited in publicly available repositories (where available and ethically appropriate), referencing such data using a unique identifier in the references and in the "Availability of Data and Materials" section of your manuscript.<br><br>Have you have met the above requirement as detailed in our Minimum Standards Reporting Checklist?	Yes

Manuscript_with_tracked_changes Click here to access/download;Manuscript;GigaScience_Organoids_R1.pdf

Journal of X, 2023, 1–13

DOI: xx.xxxx/xxxx
Manuscript in Preparation
Data note

Multi-omic dataset of patient-derived tumor organoids of neuroendocrine neoplasms

Nicolas Alcala[1,*,†], Catherine Voegele[1], Lise Mangiante[1,2], Alexandra Sexton-Oates[1], Hans Clevers[3,4,5], Lynnette Fernandez-Cuesta[1], Talya L. Dayton[3,4,6,†] and Matthieu Foll[1,*,†]

[1]Rare Cancers Genomics Team (RCG), Genomic Epidemiology Branch (GEM), International Agency for Research on Cancer/World Health Organisation (IARC/WHO), Lyon, 69008, France
[2]Department of Medicine, Stanford University, Stanford, USA
[3]Hubrecht Institute, Royal Netherlands Academy of Arts and Sciences (KNAW) and UMC Utrecht, 3584 CT Utrecht, the Netherlands
[4]Oncode Institute, Hubrecht Institute, 3584 CT Utrecht, the Netherlands
[5]Current address: Roche Pharmaceutical Research and Early Development, Basel, Switzerland
[6]Current address: European Molecular Biology Laboratory (EMBL) Barcelona, Barcelona, Spain

*Correspondence address. 25 avenue Tony Garnier CS 90627 69366 Lyon Cedex 07, France. E-mail: alcalan@iarc.who.int (N.A.), follm@iarc.who.int (M.F.)
†These authors jointly supervised this work.

Abstract

Background: Organoids are three-dimensional experimental models that summarize the anatomical and functional structure of an organ. Although a promising experimental model for precision medicine, patient-derived tumor organoids (PDTOs) have currently been developed only for a fraction of tumor types.
Results: We have generated the first multi-omic dataset (whole-genome sequencing, WGS, and RNA-sequencing, RNA-seq) of PDTOs from the rare and understudied pulmonary neuroendocrine tumors (n = 12; 6 grade 1, 6 grade 2), and provide data from other rare neuroendocrine neoplasms: small intestine (ileal) neuroendocrine tumors (n = 6; 2 grade 1 and 4 grade 2) and large-cell neuroendocrine carcinoma (n = 5; 1 pancreatic and 4 pulmonary). This dataset includes a matched sample from the parental sample (primary tumor or metastasis) for a majority of samples (21/23) and longitudinal sampling of the PDTOs (1 to 2 time-points), for a total of n = 47 RNA-seq and n = 33 WGS. We here provide quality control for each technique, and provide the raw and processed data as well as all scripts for genomic analyses to ensure an optimal re-use of the data. In addition, we report gene expression data and somatic small variant calls and describe how they were generated, in particular how we used WGS somatic calls to train a random-forest classifier to detect variants in tumor-only RNA-seq. We also report all histopathological images used for medical diagnosis: hematoxylin and eosin-stained slides, brightfield images, and immunohistochemistry images of protein markers of clinical relevance.
Conclusions: This dataset will be critical to future studies relying on this PDTO biobank, such as drug screens for novel therapies and experiments investigating the mechanisms of carcinogenesis in these understudied diseases.

Keywords: organoid, cancer, neuroendocrine neoplasm, genomics, transcriptomics, quality control

Data Description

Context

Organoids are three-dimensional experimental models that summarize the anatomical and functional structure of an organ [1, 2]. Organoids are revolutionizing fundamental and medical research by allowing us to recapitulate human physiology better than animal models, and also allowing to recapitulate developmental biology contrary to traditional cell cultures [2]. Patient-derived tumor organoids (PDTOs) have been successfully derived for tumors, providing the experimental tools to model disease progression and the preclinical models for personalized treatment testing [3, 4, 5]. Although a promising experimental model, PDTOs have currently been developed only for a fraction of tumor types, focusing on the most frequent cancers and those easiest to culture, leaving rare cancers without appropriate experimental models.

We have recently described one of the very first patient-derived organoid biobanks for the rare and understudied neuroendocrine neoplasms [6]. Neuroendocrine neoplasms are rare tumors that can arise in multiple body sites, predominantly in the lung and gastrointestinal tract [7, 8, 9]. Neuroendocrine neoplasms are further classified into neuroendocrine tumors (NETs) and neuroendocrine carcinomas (NECs). NETs are themselves subdivided into grades (ranging from 1 to 2 or 3 depending on the organs), while NECs are subdivided into small cell and large cell (LCNEC). While small cell carcinomas are more common (e.g., 15% of lung tumors), benefited from more studies and have dedicated treatment options [10], the best treatment option for LCNEC is still unclear [11], and although most NETs progress slowly and have a good prognosis,

Compiled on: December 18, 2023.
Draft manuscript prepared by the author.

Key Points

- Tumor-derived organoids are revolutionary experimental resources to test biological hypotheses and treatment options
- We have generated the first multi-omic dataset for neuroendocrine tumor organoids of the lung, and for the rare neuroendocrine tumors of the pancreas, and small intestine (ileum)

a subgroup of tumors metastasize and relapse [12].

We report here the multi-omic dataset (whole-genome sequencing, WGS, and RNA-sequencing, RNA-seq) of the neuroendocrine neoplasm PDTO biobank described in [6] (see Table 1). The dataset contains PDTOs of the lung (n = 12; 6 grade 1, 6 grade 2) and small intestine ileum (n = 6; 2 grade 1 and 4 grade 2), and LCNEC of the lung (n = 4) and pancreas (n = 1). This dataset includes longitudinal sampling of the organoids (2 to 3 time-points), and sequencing of the matched parental tumor for most samples (21/23, either primary tumors or metastases). Along with raw and processed data, we provide quality controls for each technique and scripts to run a complete molecular analysis. We also report hematoxylin and eosin-stained (H&E) slides for parental tumors and organoids, brightfield images of organoids, and immunohistochemistry images of neuroendocrine markers (Chromogranin A, Synaptofisin, CD56, and proliferation marker Ki67) and the EGFR protein. This unique dataset will provide a reference for future research on the understudied neuroendocrine neoplasms.

Methods

Sample collection

PDTO lines of the biobank described in [6] were established from surgical resections or biopsies, put in culture and expanded. PDTOs periodically underwent passaging, a process by which organoids are subcultured to allow future growth [13]; passage time varied from a week to several months depending on the growth rate ([6] Fig. 2). H&E stainings were performed and samples underwent an independent pathological review, and immunohistochemistry of common neuroendocrine markers (Chromogranin A, synaptophysin) were performed to confirm the tumoral neuroendrocrine nature of the parental tumors and PDTOs. See [6] for a detailed description of the protocol, and the GigaDB repository associated with this article for digital versions of H&E stainings and immunohistochemistry.

Extraction

For each tumor or PDTO, DNA and RNA were extracted from the same sample using the QIAGEN All Prep DNA/RNA Mini kit.

Sequencing

Whole-GenomeSequencing (WGS). Whole-genome sequencing was performed by the Utrecht Sequencing Facility. After DNA quality control, genomic DNA (0.5–1 μg) was used to prepare the whole-genome sequencing library, using the Illumina TruSeq DNA Nano Kit. Libraries were then sequenced on a Novaseq 6000 platform, as paired-end 150 bp reads, with a target average coverage of 30X for normal samples and 60X to 90X for tumor tissue and PDTOs.

RNA-Sequencing (RNA-seq). RNA sequencing was performed by the Utrecht Sequencing Facility. After RNA quality control, libraries were prepared using the Illumina TruSeq Stranded mRNA polyA Kit. Libraries were sequenced either on a Nextseq 2000 or an Illumina Novaseq 6000, as paired-end 150bp reads.

Data processing

All data processing was performed using the workflows developed by the rare cancers genomics team of the International Agency for Research on Cancer / World Health Organization (https://github.com/IARCbioinfo/), as detailed in [14] and [15]. The workflows are written in the popular domain-specific language nextflow [16]. All software dependencies are contained in conda environments and containerized with Docker and Singularity (containers available at https://hub.docker.com/ and https://singularity-hub.org/).

WGS. Raw reads were mapped to reference genome GRCh38 using workflow *alignment-nf* (https://github.com/IARCbioinfo/alignment-nf, v1.2). This workflow first maps reads (software bwa-mem2 v2.0 [17, 18]), then marks duplicates (software samblaster, v0.1.26 [19]), and finally sorts reads (software sambamba, v0.7.1 [20]).

RNA-seq. Raw reads were mapped to reference genome GRCh38 with annotation gencode v33 using the workflow *RNAseq-nf* (https://github.com/IARCbioinfo/RNAseq-nf, v2.4). This workflow removes adapter sequences (wrapper Trim Galore v0.6.5 [21] for software cutadapt [22]), maps reads (software STAR v2.7.3a [23]), marks duplicated reads (software samblaster, v0.1.25), and finally sorts reads (software sambamba, v0.7.1).

Alignments were then post-processed using two workflows to improve their quality. Workflow *abra-nf* (https://github.com/IARCbioinfo/abra-nf, v3.0) performs local realignment using software ABRA2 (v2.22 [24]), and *BQSR-nf* (https://github.com/IARCbioinfo/BQSR-nf, v1.1) performs base quality score recalibration using gatk (v4.0.5.1 [25]).

Variant calling from WGS. Single nucleotide variants were called on all WGS samples using software Mutect2 from GATK4 (v4.2.0.0 [26, 27]) with workflow *mutect-nf* (https://github.com/IARCbioinfo/mutect-nf, v2.2b), as described in [6]. Resulting variant calling format (VCF) files were normalized using bcftools v1.10.2 [28] (workflow https://github.com/IARCbioinfo/vcf_normalization-nf, v1.1) and annotated using ANNOVAR v2020Jun08 (workflow https://github.com/IARCbioinfo/table_annovar-nf v1.1.1). Indels and multinucleotide variants were additionally filtered using the intersection of Mutect2 and strelka2 [29] calls (workflow https://github.com/IARCbioinfo/strelka2-nf v1.2a), in order to reduce false positives that are more frequent in indel calls due to the difficulty of detecting such variants with short reads sequencing.

Variant calling from RNA-seq. Variants were called on all RNA-seq samples using software Mutect2 from GATK4 (v4.2.0.0 [26, 27]) with workflow *mutect-nf* (https://github.com/IARCbioinfo/mutect-nf, branch RNAseq), in RNA-seq and tumor-only modes. The RNA-seq mode incorporates a preprocessing step to fix CIGAR strings (removing NDN elements and ensuring that mapping quality 255 is not used as some mappers like STAR can do), and GATK4's SplitNCigarReads method that splits reads with Ns in their CIGAR string, in order to improve variant calling quality.

Table 1. Sample summary

ID	Primary site	Tumor type	WGS	RNA-seq	Normal sample (ID)	Tumor sample (ID)	Organoid passages (IDs)
LCNEC1	pancreas	LCNEC	yes	yes	blood (PANEC1N)	primary (PANEC1T)	4 (PANEC1Tp4), 14 (PANEC1Tp14)
LNET2	lung	NET (G1)	yes	no	normal-derived organoid passage 7 (LNET2Np12)	primary (LNET2T)	12 (LNET2Tp12), normal-derived organoid passage 12 (LNET2Np12)
LCNEC3	lung	LCNEC	yes	yes	tissue (LCNEC3N*)	primary (LCNEC3T)	17 (LCNEC3Tp17.2), 24 (LCNEC3Tp24)
LCNEC4	lung	LCNEC	yes	yes	normal-derived organoid passage 6 (LCNEC4Np6)	primary (LCNEC4T)	7 (LCNEC4Tp7), 24 (LCNEC4Tp24)
LNET5	lung	NET (G1)	yes	yes	blood (LNET5N)	primary (LNET5T)	4 (LNET5Tp4), 7 (LNET5Tp7), 2 (LNET5Tp2.2)†
LNET6	lung	NET (G1)	yes	yes	tissue (LNET6N)	primary (LNET6T)	1 (LNET6Tp1)
mSINET7	small intestine (ileum)	NET (G2)	yes	yes	blood (SINET7N)	mesenteric metastasis (SINET7M)	2 (SINET7Mp2)
mSINET8	small intestine (ileum)	NET (G2)	yes	yes	blood (SINET8N)	ovary metastasis (SINET8M)	2 (SINET8Mp2)
mSINET9	small intestine (ileum)	NET (G2)	yes	no	blood (SINET9N)	mesenteric metastasis (SINET9M)	1 (SINET9Tp1)
LNET10	lung	NET (G2)	yes	yes	blood (LNET10N)	primary (LNET10T)	4 (LNET10Tp4)
mLCNEC11	lung	LCNEC	no	yes	none	brain metastasis (LCNEC11M)	3 (LCNEC11Mp3)
mSINET12	small intestine (ileum)	NET (G2)	no	yes	none	mesenteric metastasis (SINET12M)	1 (SINET12Mp1 and SINET12Mp1.3)‡
LNET13	lung	NET (G1)	no	yes	none	primary (LNET13T)	1 (LNET13Tp1)
LNET14	lung	NET (G1)	no	yes	none	primary (LNET14T)	1 (LNET14Tp1)
mLNET15	lung	NET (G2)	no	yes	none	skin/soft tissue metastasis (LNET15M)	2 (LNET15Mp2)
LNET16	lung	NET (G2)	no	yes	none	primary (LNET16T)	2 (LNET16Tp2)
mLNET16	lung	NET (G2)	no	yes	none	metastasis to the ribcage (LNET16M)	1 (LNET16Mp1)
LNET18	lung	NET (G2)	no	yes	none	none	2 (LNET18Tp2, from primary)
LNET19	lung	NET (G1)	no	yes	none	primary (LNET19T)	2 (LNET19Tp2)
mLNET20	lung	NET (G2)	no	yes	none	paravertebral Th1 mestastasis (LNET20M)	2 (LNET20Mp2)
mSINET21	small intestine (ileum)	NET (G1)	no	yes	none	paravertebral Th1 mestastasis (SINET21M)	2 (SINET21Mp2)
mSINET22	lung	NET (G1)	no	yes	none	paravertebral Th1 mestastasis (SINET22M)	2 (SINET22Mp2)
mLCNEC23	unknown	LCNEC	no	yes	none	none	3 (LCNEC23Mp3, from paravertebral Th1 mestastasis)

for the normal samples, only WGS was performed
* one normal tissue for this experiment was excluded due to discordance with the tumor (see Fig. 4)
† Two lines were derived for LNET5, one sequenced at passages 4 and 7 (samples LNET5Tp4 and LNET5Tp7) and one at passage 2 (LNET5Tp2.2)
‡ Two lines were derived for SINET12, each sequenced at passage 1 (samples SINET12Mp1.1 and SINET12Mp1.3)

Resulting variant calling format (VCF) files were normalized using bcftools v1.10.2 [28] (workflow https://github.com/IARCbioinfo/vcf_normalization-nf, v1.1) and annotated using ANNOVAR v2020Jun08 (workflow https://github.com/IARCbioinfo/table_annovar-nf v1.1.1). For samples which also had WGS data, RNA-seq-detected variants were classified as somatic or germline based on the WGS variant calls described above.

Quality control

For each 'omic technique, quality controls (QC) of the samples were performed at each step.

Raw reads

Software FastQC (v0.11.9 [30]) was used to check raw reads quality, and software MultiQC (v1.9 [31]) was used to aggregate the QC results across samples and generate interactive plots; all plots from Figs. 1 and 2 were generated by multiQC from the FastQC outputs. Original MultiQC reports are available in Supplementary Information (Files S1-S4) to allow a free exploration of the QC statistics.

WGS. Raw reads passed quality control filters in all samples. All samples displayed good sequence quality scores (mode above 30 Phred, indicating an error rate below 0.2%), both on average and across all positions in the read (Fig. 1A and B), with samples sequenced later (lower part of Table 1, from LNET5 to LNET10) displaying better scores (highest mode in Fig. 1A). GC content were slightly skewed toward lower values but proved consistent across samples (Fig. 1C), and adapter content (Fig. 1D, less than 5% of sequences with adapter sequence detected) and duplication levels (Fig. 1D, less than 20% of sequences present twice or more) were adequate and E). The number of reads were consistent between read pairs and consistent with target read depths (Fig. 1F): samples with a target depth of 30X-normal, normal-derived organoids, the primary tumor from experiment LCNEC1, and tumor organoid passage 14 from experiment LCNEC3 (LCNEC3Tp14)-having a lower number of reads ($\sim 4 \times 100$M reads= 400M reads) than the others samples ($\sim 4 \times 250M$ = 1000M reads), which had a target depth of 90X. Note that the metastasis organoid of experiment SINET9 (SINET9Mp1) has been sequenced in eight lanes, with 4 lanes with a low number of reads ($\sim 30M$) and 4 additional ones with a larger number ($\sim 140M$) so the total is comparable with that of the other samples.

RNA-seq. Raw reads passed quality filters after reads trimming for adapter content and quality. All samples displayed good sequence quality scores on average both before and after read trimming (mode above 30 Phred; Fig. 2A and B), with samples sequenced later (lower part of Table 1, from LNET5 to LNET14) displaying better scores (highest mode in Fig. 1A). Six samples displayed lower scores at the end of the reads before trimming (Fig. 2C) but better scores after trimming (Fig. 2D). Indeed, most samples displayed high adapter content before trimming (Fig. 2E), and the trimming step successfully removed them (less than 0.1% in all samples; Supplementary Information File S1). The trimming step mostly removed less than 5 bp from the read, but occasionally could remove up to around 50 bp (Fig. 2F). GC content were consistent across samples (Fig. 2G-H), although the read trimming step resulted in an excess of reads with high GC content, presumably due to some reads being strongly shortened by the trimming step. Hopefully, in general the trimming step did not increase much the proportion of short reads (Fig. 2I-J). The number of reads were consistent between read pairs and across sequencing runs both before and after trimming (Fig. 2K-L), and total read numbers for each sample were consistent with the target number of 50M (25M pairs): the smallest number, 60.8M corresponded to sample PANEC1Tp14.

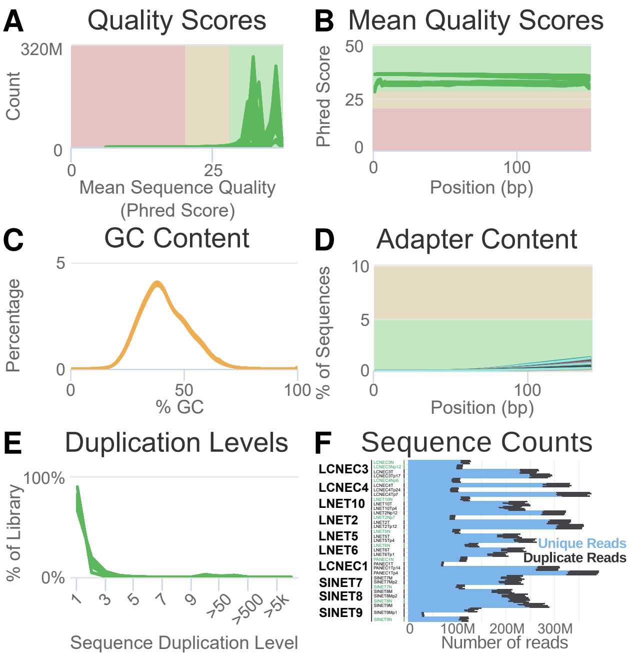

Figure 1. Quality control of the raw Whole-Genome Sequencing (WGS) data. (A) Distribution of the mean sequence quality of the reads in Phred score. (B) Mean sequence quality score as a function of the position in the read in base pairs (bp). (C) Distribution of the GC content in percent. (D) Percentage of reads containing a sequence corresponding to the Illumina adapter sequence as a function of the position in the read in bp. (E) Percentage of the library with a given level of duplication. (F) Number of unique and duplicated reads per file. In panels (A)-(E), each line corresponds to a fastq file, with each of the 34 samples from Table 1 subdivided into four sequencing lanes (except SINET9Mp1, subdivided into 8 lanes), and additionally subdivided into two read pair files, for a total of $4 \times 2 \times 33 + 8 \times 1 = 280$ files; in panel (F), each horizontal bar corresponds to a file. In (A)-(E), green lines correspond to files that passed the most stringent QC filters of software FastQC; orange lines correspond to files that passed a less stringent filter.

Alignments

WGS. The software qualimap (v2.2.2b [32]) was called by our workflow *alignment-nf* to generate QC statistics for the WGS alignments in parallel to the data processing (Table 2). All normal and normal tissue-derived organoids displayed a mean coverage $\geq$30X, and all tumor and tumor-derived organoids except passage 24 from the organoid of experiment LCNEC4 (sample LCNEC4Tp24) and passage 1 of the organoid of experiment SINET9 (sample SINET9Mp1) had a coverage $\geq$ 60X; all samples displayed at least 65% of the genome with a coverage larger than or equal to 30X except LCNEC4Tp24 and (57.4%). Percentages of aligned reads exceeded 99.8% for all samples. Interestingly, some tumor and tumor-derived organoid samples displayed bimodal coverage distributions compatible with variations in copy number state (Supplementary Information File S3).

RNA-seq. Software RSeQC (v3.0.1 [33]) was called to check alignment quality in parallel to the data processing by workflow *RNAseq-nf*. For all samples, the number of known junctions (i.e., junctions annotated in the gencode v33 annotation file) was stable when resampling subsets of 75% to a 100% of the reads (all lines plateau in Fig. 3A), indicating a good saturation and suggesting that the sequencing depth was sufficient to detect known junctions. In contrast, the number of novel junc-

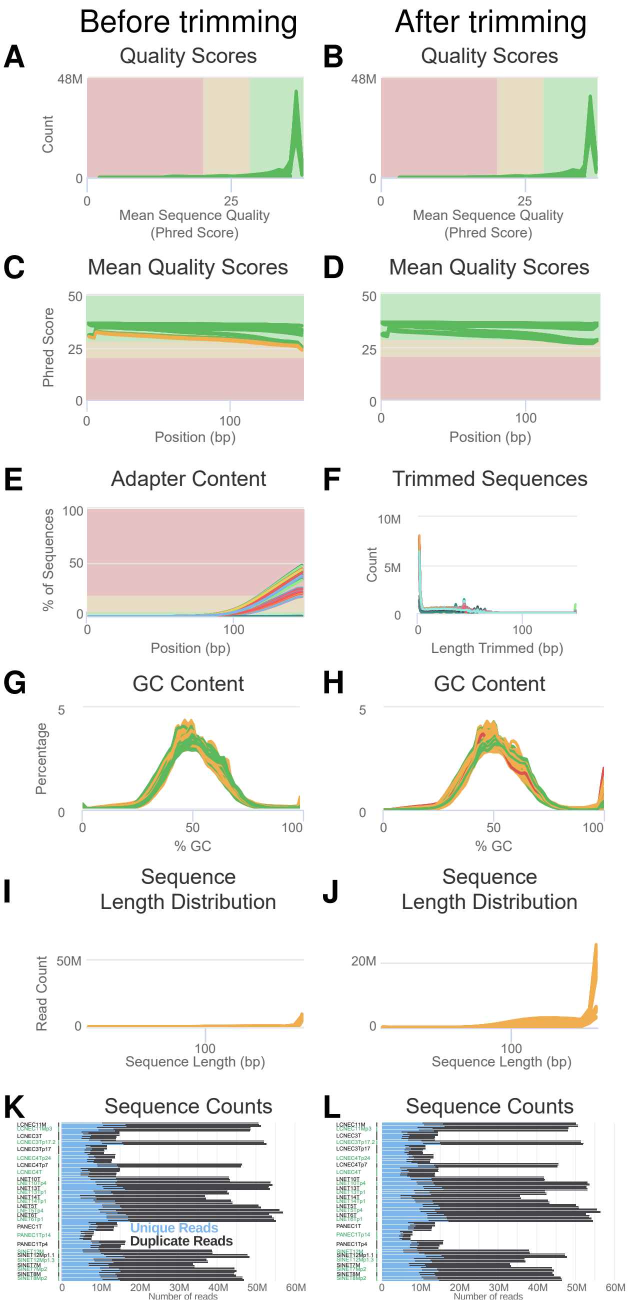

Figure 2. Quality control of the raw RNA-seq data. Panels (A), (C), (E), (G), (I), (K) correspond to controls before read trimming for quality and adapter content by wrapper Trim Galore for software cutadapt; panels (B), (D), (F), (H), (J), (L) correspond to controls after read trimming. Figure legends for panels (A)–(E) and (G)–(L) follow that of Fig. 1. (F) Distribution of the length of the reads trimmed by software cutadapt, for each file (colored lines). In panels (A)–(J), each line corresponds to a fastq file, with each of the 10 non-normal samples from Table 1 divided into two or four sequencing lanes, and further subdivided into two read pair files, for a total of $2 \times 2 \times 21 + 4 \times 2 \times 7 = 140$ files; in panels (K)–(L), each horizontal bar corresponds to a file.

tions (i.e., junctions not in the annotation file) was increasing slowly as a function of the percentage of reads resampled, but did not completely saturate (no complete plateau in Fig. 3B). This indicates that we probably detected the most abundant novel junctions but that some low abundance novel junctions were probably not detected.

Alignment scores were good, with more than 25M mapped read pairs (50M reads) for all samples, and from 4M to 7M unmapped reads, mainly due to reads being too short or having too many mismatches (Fig. 3C). The distribution of the alignments within annotated regions matched our expectations, with most reads (≥80%) either aligning to exons (≥50%), 3' UTR (~3%), and 5' UTR (~25%) (Fig. 3D).

Table 2. Quality control of the WGS alignments

Sample Name	% GC	≥30X	≥50X	Coverage	% Aligned
PANEC1N	42%	84.6%	16.2%	41.0X	99.9%
PANEC1T	42%	93.8%	91.7%	104.0X	99.8%
PANEC1Tp4	42%	93.9%	93.3%	127.0X	99.9%
PANEC1Tp14	42%	93.6%	90.9%	89.0X	99.8%
LNET2Np7	41%	67.2%	1.9%	33.0X	99.9%
LNET2Np12	41%	93.4%	92.9%	104.0X	99.9%
LNET2T	41%	93.3%	92.9%	109.0X	99.9%
LNET2Tp12	42%	93.4%	93.1%	115.0X	99.8%
LCNEC3N	41%	82.5%	11.1%	39.0X	99.9%
LCNEC3Np12	42%	82.5%	11.7%	38.0X	99.9%
LCNEC3T	41%	93.9%	90.0%	89.0X	99.9%
LCNEC3Tp17	42%	93.5%	90.9%	90.0X	99.8%
LCNEC4Np6	42%	69.7%	2.7%	34.0X	99.9%
LCNEC4T	41%	93.0%	88.1%	102.0X	99.8%
LCNEC4Tp7	42%	91.7%	87.9%	102.0X	99.9%
LCNEC4Tp24	42%	51.9%	11.3%	30.0X	99.9%
LNET5N	41%	68.9%	2.5%	33.0X	99.9%
LNET5T	42%	91.9%	77.6%	68.0X	99.9%
LNET5Tp4	42%	93.3%	87.6%	75.0X	99.9%
LNET6N	42%	86.6%	22.8%	43.0X	99.9%
LNET6T	42%	93.0%	83.1%	72.0X	99.9%
LNET6Tp1	42%	90.2%	76.8%	61.0X	99.9%
SINET7N	42%	77.2%	4.9%	36.0X	99.9%
SINET7M	41%	92.8%	83.5%	73.0X	99.9%
SINET7Mp2	42%	92.8%	85.7%	69.0X	99.9%
SINET8N	42%	93.1%	91.7%	75.0X	99.9%
SINET8M	41%	92.6%	81.2%	64.0X	99.9%
SINET8Mp2	42%	93.0%	85.5%	70.0X	99.9%
SINET9N	42%	84.4%	7.2%	38.0X	99.9%
SINET9M	41%	93.0%	90.0%	81.0X	99.9%
SINET9Mp1	42%	90.2%	49.1%	49.0X	99.9%
LNET10N	42%	86.4%	9.5%	39.0X	99.9%
LNET10T	42%	93.0%	90.1%	71.0X	99.9%
LNET10Tp4	42%	93.0%	87.1%	66.0X	99.9%

Data Validation

Sample matching

We used software NGSCheckMate (cloned from the github repository https://github.com/parklab/NGSCheckMate revision 10799087bdfe4b990add5b5e536f87c47bbdb688) to check that samples from the same experiment indeed came from the same individual, in both WGS and RNA-seq simultaneously, using our workflow *NGSCheckMate-nf* (https://github.com/IARCbioinfo/NGSCheckMate-nf, v1.1). The sample matching algorithm correctly identified all experiments except one (Fig. 4). The WGS normal-derived organoid sample from experiment LCNEC3 (LCNEC3Np12_WGS in Fig. 4) was found not to match other LCNEC3 samples, suggesting a possible sample swap and was thus excluded from further analyses. Also, the RNA-seq tumor sample for the late-passage organoid of experiment LCNEC3 (sample LCNEC3Tp17_RNA in Fig. 4) was found to better match experiment LNET2, and was thus excluded from the subsequent analyses. Finally, two samples were found to par-

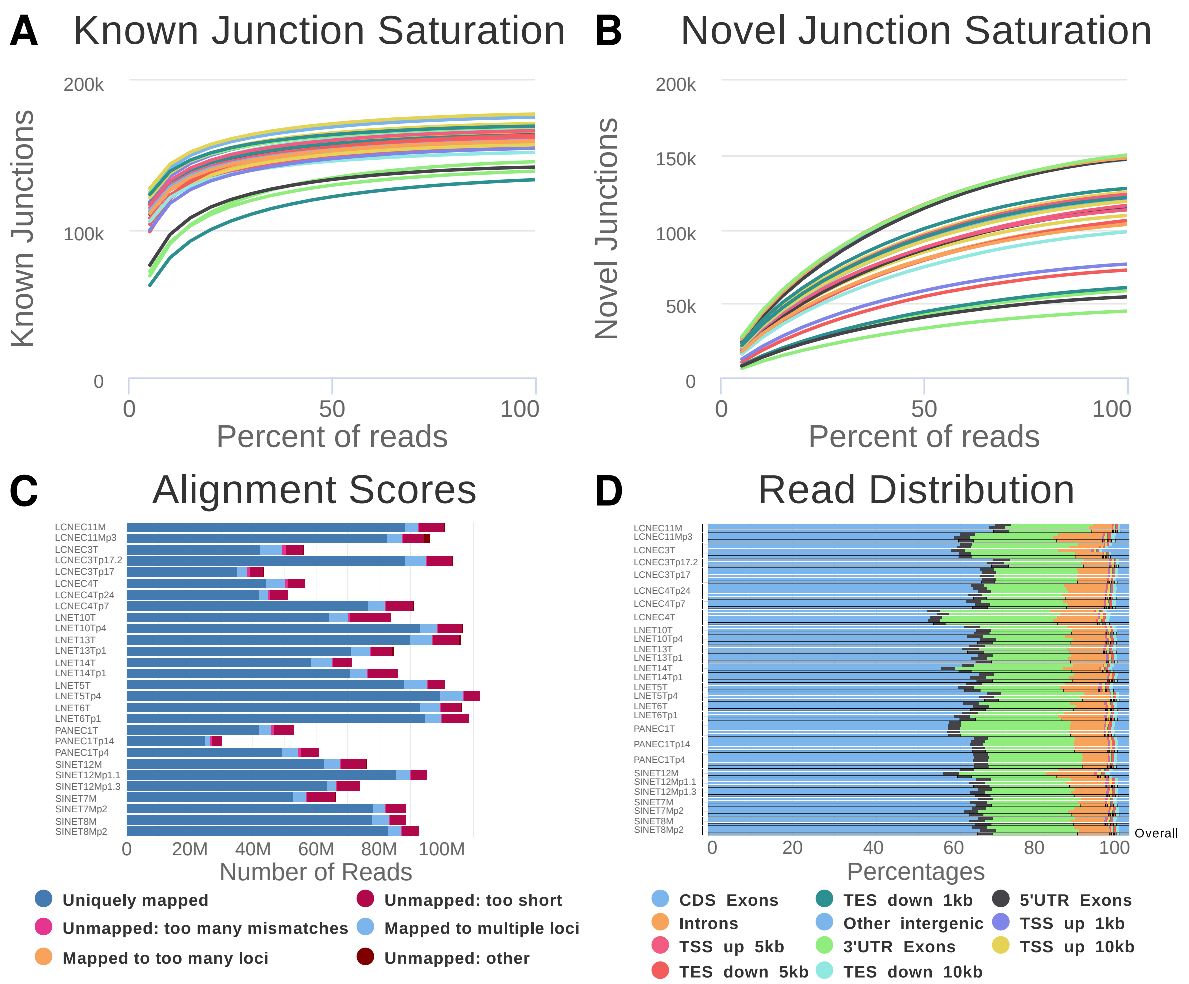

Figure 3. Quality control of the RNA-seq alignments. (A) Number of known junctions identified by software STAR in a subsample as a function of the percentage of reads in the subsample. (B) Number of novel junctions identified by STAR in a subsample as a function of the percentage of reads in the subsample. (C) Number of sequence tags with each alignment score. (D) Distribution of reads among annotated regions.

tially match LNET15 and LNET16, suggesting contamination and were also excluded (UNKN00 and UNKN01).

Sex validation

We validated the sex reported in the clinical data using the multi-omic data. For the WGS data, we used the proportion of reads aligned to the sex chromosomes to assess whether samples clustered by sex (Fig. 5A). We found that all samples clustered by sex except for the normal of experiment LCNEC3 (sample LCNEC3Np12) which clustered with females despite other samples from the experiment clearly clustering with males. This further supports the sample matching reports that suggest that this sample does not match the rest of the experiment. For the RNA-seq data, we compared the total expression level on the sex chromosomes, using the variance-stabilised read counts as a quantification of gene expression (vst function from R package DESeq2 v1.26.0 [34]) (Fig. 5B). We find that samples from the same sex cluster together for all experiments, suggesting concordance with the clinical data.

Small variant calls from RNA-seq

We classified small variants called from RNA-seq in 241 known neuroendocrine neoplasm driver genes (from Table S4 in [6]) as somatic or germline, using a random forest (RF) algorithm [35] (R package randomForest v4.7-1.1 [36]; Fig. 6), using a similar approach as we recently did to classify mutations in tumor-only WGS [37]. After filtering out non-exonic, synonymous, and nonsynonymous mutations with a REVEL score [38] below 0.5, and mutations not in the list of 241 drivers, we were left with 2430 variants. Among them, 1174 variants were in samples with WGS data available and their somatic status was thus known.

We used 10 features in the RF model. One feature was directly informative about the potential germline status and came from a public database: the frequency of the allele in human populations from the ExAc database excluding cancers from the TCGA (feature *ExAC_nontcga_ALL*). Four features were informative about the alignment and came from the sequencing data itself: the median distance from the end of the read (feature *MPOS*), the likelihood ratio score of variant existence (feature *TLOD*), the coverage at the position (feature *DP*), and the allelic fraction of the alternative allele (RNA.AF). Finally, the other features were informative about the pathogenicity of the variant and came from public databases: the REVEL score of pathogenicity (feature *REVEL*), the presence in the COSMIC 92 database (feature *cosmic92_coding_nonnull*), the presence in the COSMIC 92 database in a lung tumor (feature *cosmic92_coding_lung*), and the InterVar annotation (feature *InterVar_automated*; with levels ".", "Uncertain_significance", "likely_pathogenic", and "Pathogenic"), and the exonic function of the variant (missense, nonsense, inframe or frameshift insertion, etc).

The RF algorithm was trained and tested on the 1174 variants with known status (1148 germline, 26 somatic) called in 22 samples from 8 experiments (Fig. 6A). Note that although the data is imbalanced, we chose to keep this imbalance in

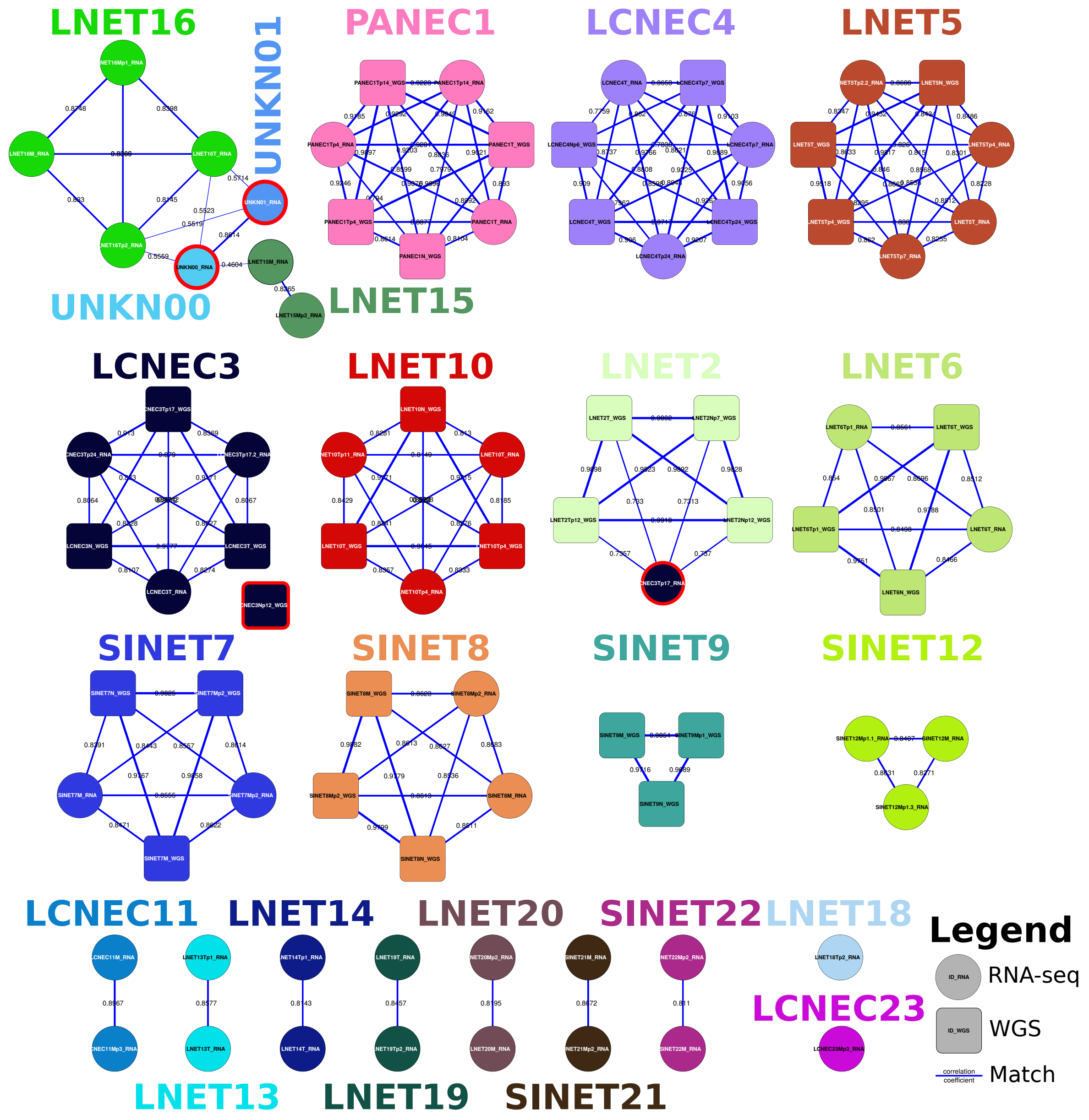

Figure 4. Network of matches between WGS and RNA-seq samples, computed with software NGSCheckmate. Numbers on the edges and edge thickness correspond to the Pearson correlation coefficient *r* between allelic fractions for the germline SNP panel; colors: experiments (see Table 1); squares: WGS, circles: RNA-seq, red contour: mismatches.

the training set to force the algorithm to take into account the fact that most variants are not somatic, and thus having a very good specificity is key to avoid large false discovery rates. We used leave-one-out cross-validation at the experiment level (8 folds), excluding all samples from one same experiment from the model fit at each iteration in order to avoid over-fitting due to the inclusion of variants from the same individual but different samples (e.g., LCNEC3T and LCNEC3Tp17) in the training and test sets. We used 5000 trees, and 3 features per split (the square root of the total number of features as recommended by default), and a minimal node size of 1. We estimated the performance of the model using the receiver operating characteristic (ROC) curve and its area under the curve (AUC, computed using the trapezoid rule), showing the sensitivity as a function of 1-specificity across different thresholds for the proportion of votes for the somatic class. We also computed the false discovery rate to get a sense of the proportion of variants classified as somatic that would actually be false positives. Once the RF model performance was assessed, we trained a RF model on the full 1174 variants and predicted the status of the remaining 1256 variants. See github repository `https://github.com/IARCbioinfo/MS_panNEN_organoids` for the complete R script. Note that the same approach allowed to classify variants called from tumor-only WGS data as somatic or germline with high performance (accuracy greater than 92%; Di Genova et al. 37).

We find that we can classify variants as somatic or germline with a balanced accuracy of 86%, with both specificity greater than 98% and sensitivity greater than 73% (AUC=0.965). Interestingly, although somatic variants are just a fraction of the calls (2%), the high sensitivities and specificities of our RF algorithm allowed to classify variants with false discovery rates below 50% while still preserving sensitivities above 60% (see Fig. 6B, E-G). We also tested the predictive accuracy of the model fitted on this set of 1174 variants from known neuroendocrine neoplasm genes on the set of somatic variants from other recurrently mutated genes in our cohort (Fig. S1). We find that the predictive power of the RF model was similar

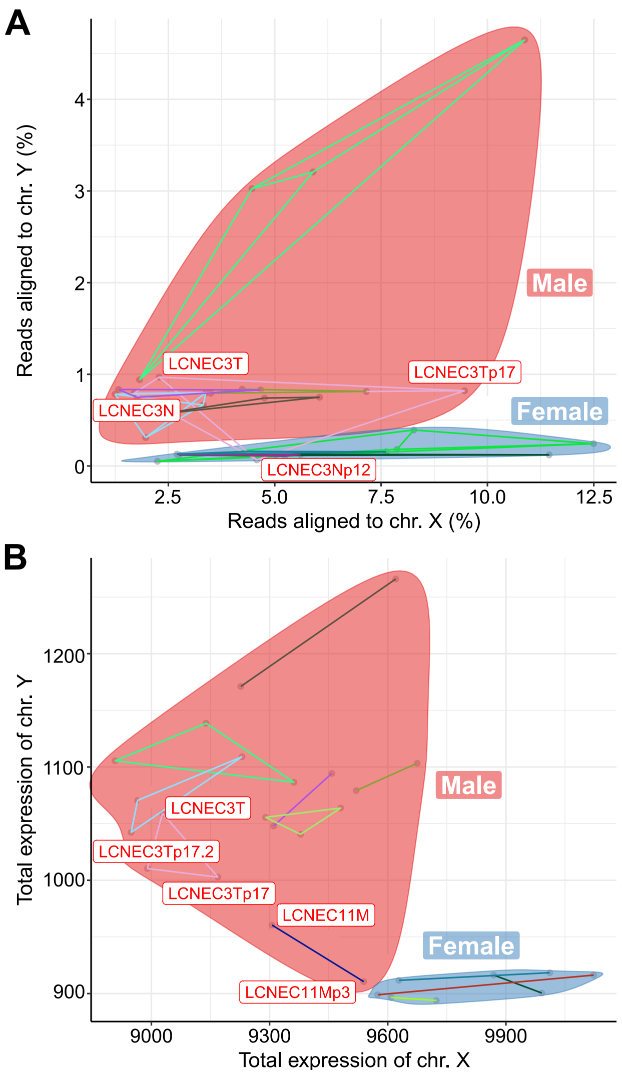

Figure 5. Validation of reported sex. (A) Percentage of reads aligned to chromosome X and Y in the whole-genome sequencing data. (B) Total gene expression in X and Y chromosome, in units of variance-stabilized read counts, computed from RNA-seq data. In all panels, samples from each sex are encircled (red:male, blue:female), excluding LCNEC3Np12, which we report as not matching the other samples from the LCNEC3 experiment.

(AUC=0.90, sensitivity up to 73% with a specificity above 87%).

We evaluated the importance of features for the classification both using the mean decrease in accuracy, which captures how much the model loses accuracy when the feature is excluded, and using the mean tree depth at which the feature was observed, with a low value meaning that the feature is used early in the decision trees and thus separates many variants[35, 39] (R package randomForestExplainer v0.10.1). The most important features for the classification were the REVEL score, the TLOD, and the cosmic annotation, while the frequency in the ExAC database was the least important, presumably because all these variants were very rare (Fig. 6C). Indeed, the most representative tree from the RF, computed using the reprtree R package v0.6 using the d2 distance metric between tree predictions[40], relied on these three variables, with all alterations present in a lung tumor from the COSMIC 92 database automatically classified as somatic (root of the tree), and TLOD and REVEL score being the most common features used for splitting (Fig. 6D). Of note, using the most important feature alone (the REVEL score) led to a much lower accuracy, consistent with the importance of other features such as TLOD and pathogenic annotations (COSMIC, InterVar).

Comparing molecular profiles of PDTOs and parental tumors

We report here all the R scripts used in Dayton et al. [6] to validate that PDTOs faithfully represent their parental tumors (available at https://github.com/IARCbioinfo/MS_panNEN_organoids). In particular, we provide the code that we used to compare the expression profiles of PDTOs and reference lung and SI NETs and LCNECs with that of PDTOs and their parental tumors (https://github.com/IARCbioinfo/MS_panNEN_organoids/blob/main/Rscripts/Fig3/Fig3B_S3BCE.md). This analysis confirmed the neuroendocrine nature of the PDTOs by showing that they express neuroendocrine markers routinely used in the clinic (>1 TPM in at least one of 6 markers). We also provide the code (https://github.com/IARCbioinfo/MS_panNEN_organoids/blob/main/Rscripts/Fig3/Fig3CD_S3FGHI.md) used in Dayton et al. [6] to demonstrate that pure PDTOs preserve the expression profiles of their parental tumor using dimensionality reduction techniques (UMAP). In addition, we provide the code (https://github.com/IARCbioinfo/MS_panNEN_organoids/blob/main/Rscripts/Fig4/Fig4BC_S4BC.md and https://github.com/IARCbioinfo/MS_panNEN_organoids/blob/main/Rscripts/Fig4/Fig4D_S4D.md) used to show that PDTOs preserve the genomic profile (small variants, copy number variants, and structural variants) of their parental tumor. To do so, we focused on mutations known to be drivers of neuroendocrine neoplasms [41, 42, 43, 44, 45, 46, 14, 47]. Both variants identified with WGS and variants identified with RNA-seq include driver mutations in key recurrently altered LCNEC driver genes such as *TP53* (mutated in 5/5 LCNEC) and *STK11* (mutated in 3/5 LCNEC). We also identified mutations or structural variants in known driver genes in all but one neuroendocrine tumors (17/18), but as previously reported, they involve multiple genes instead of recurrently mutated genes [48, 14]. This confirms that PDTOs recapitulate the genomic profile of neuroendocrine neoplasms.

We also report the R scripts used in Dayton et al. [6] to analyze the temporal evolution of the PDTOs (https://github.com/IARCbioinfo/MS_panNEN_organoids/blob/9e6005ac1e2bab6777b52ede585ec1b74e244acf/Rscripts/Fig5/Fig5_S5.md). These analyses showed that PDTOs preserve the genetic diversity and clonal architecture of their parents across long periods of time (6 months to more than a year). In particular, the analysis of two samples with multiple time points (LCNEC1 and LCNEC4) highlighted that the genetic makeup of the parental tumor is preserved across PDTO passages.

Of note, one sample, LCNEC23 was a paravertebral metastasis of an LCNEC of unknown primary. As mentioned in Dayton et al. [6] (Figure 3), the transcriptome of this sample did cluster with other LCNEC from the lung and pancreas; in addition, we detected from the RNA-seq two high-confidence somatic mutations characteristic of LCNEC: a nonsynonymous *TP53* and a nonsense *PIK3CA* mutation. These molecular results comfort the LCNEC nature of the PDTO, but the overlap between known lung and pancreas LCNEC profiles does not allow to infer the site of origin of the tumor.

Re-use potential

We describe here some of the very first multi-omic datasets for patient-derived tumor organoids of pancreatic, small intestine (ileum), and pulmonary neuroendocrine neoplasms, in particular including the first lung neuroendocrine tumor organoids. Because such low grade tumors are difficult to cultivate in vitro, there is currently a lack of adequate experimental sys-

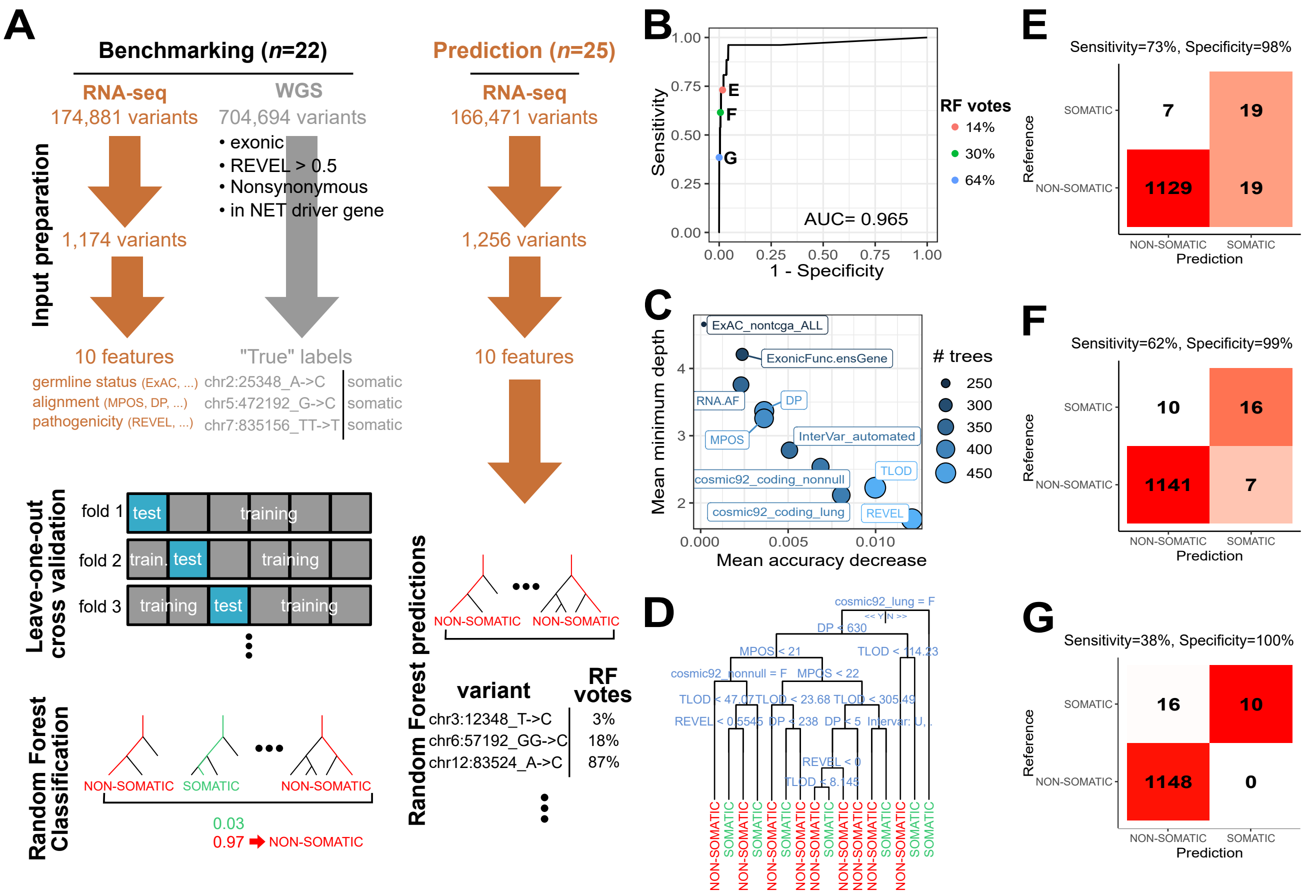

Figure 6. Random forest (RF) classification of variants as somatic or germline from RNA-seq data. (A) Schematic of the RF training, test, and prediction. B) Receiver operating characteristic (ROC) curve. C) Feature importance for classification accuracy. Mean accuracy decrease: mean difference in accuracy between trees with the feature and trees without the feature; high values indicate important features. Mean minimum depth: tree depth (1: root, value >> 1: leaves) of the first time the feature is used for classification, averaged across all trees; low values indicate features often used at the root and thus particularly important. D) Representative tree of the RF. At each split, the split condition is written above, the left branch corresponds to a Yes and the right branch to a No. Final decision (SOMATIC or NON-SOMATIC) is represented by the leaves. (E)-(G) Confusion matrix for different levels of sensitivity and specificity. Reference: somatic status assessed from whole-genome sequencing data. Prediction: somatic status predicted from RNA-seq data using the RF algorithm.

tems for these tumors, and we expect the biobank associated with the data presented here to be the basis for future experimental studies–either fundamental or treatment oriented–on neuroendocrine neoplasms across body sites. The multi-omic dataset we provide here constitutes the molecular fingerprints of these experimental models, and will be key to investigate oncogenic processes responsible for tumor initiation and progression, and to link drug responses to molecular features to design future personalized treatments.

To facilitate future studies, we used the exact same data processing as in our previous studies of neuroendocrine neoplasms [14, 15] and other rare cancers [49], in particular using rigorous RNA-seq expression quantification with containerized software and operating systems (see methods section). To ease future studies, we make the expression matrix publicly available at https://github.com/IARCbioinfo/MS_panNEN_organoids/blob/9e6005ac1e2bab6777b52ede585ec1b74e244acf/data/gene_expression_PDTOs_parents.tsv. In addition, we provide all R scripts to analyze the data at https://github.com/IARCbioinfo/MS_panNEN_organoids.

Note that the slow passage time of low-grade PDTOs makes them appropriate models to study the biology of neuroendocrine tumors, but challenges their use for drug testing. This is particularly true of small intestine NETs, which were only short term cultures that did not grow past four passage. Finally, as noted in most molecular studies of PDTOs [50], one of the main differences between PDTOs and their parental tumors is the absence of microenvironment. Future work would ideally focus on creating co-cultures of PDTOs and immune cells to remedy this shortcoming.

Conclusion

We have shown that our multi-omic dataset is of high quality and can be easily re-used. Given the rarity of neuroendocrine tumors from the lung, pancreas, and small intestine, past genomic studies each only reported data for a handful of samples, limiting the potential discoveries. For example, for lung NETs, 29 WGS and 39 RNAseq were reported in [48], 3 WGS and 20 RNA-seq in [14], and 30 RNA-seq in [51]; for small intestine NETs, for example, 81 RNA-seq with no WGS were reported in [52] and 7 RNA-seq in [53]). As a result, the primary tumors and metastasis sequencing data we report here (10 samples with WGS, 21 with RNA-seq) alone are very valuable, and should be combined with other datasets in future studies to provide enough power to discover informative molecular features for diagnosis, prognosis, and treatment. In addition, we report a unique multi-omic dataset generated from patient-derived tumor organoids, which will allow all researchers working on our biobank to test hypotheses regarding the molecular features associated with drug responses and thus advance research on personalized treatments for these understudied diseases.

Availability of source code and requirements

- Project name: NEN organoids project, lungNENomics
- Project home pages: https://www.embl.org/groups/dayton/, http://rarecancersgenomics.com/lungnenomics/
- Operating system(s): Platform independent
- Programming language: Nextflow, R
- Other requirements: R packages *caret*, *randomForest*
- License: GNU GPL

All nextflow command lines for data processing are available at https://github.com/IARCbioinfo/MS_panNEN_organoids in the readme. All R scripts for the analysis are available in the subfolder https://github.com/IARCbioinfo/MS_panNEN_organoids/Rscripts.

Availability of supporting data and materials

The data set supporting the results of this article is available in the European Genome-Phenome archive repository, study EGAS00001005752. The study consists of seven datasets: EGAD00001009988, with WGS CRAM files for 2 experiments, EGAD00001009989 with WGS CRAM files for 6 experiments, EGAD00001009990, with WGS CRAM files for 2 experiments, EGAD00001009991 with RNA-seq fastq files from 4 experiments, EGAD00001009992, with RNAseq fastq files for 15 experiments, EGAD00001009993, with RNA-seq fastq files for 2 experiments, and EGAD00001009994, with gene expression in multiple formats (R data, tab-separated text files) and multiple units (raw counts, TPM, FPKM) for 21 samples. Because of the sensitivity of the data and the patient consent, to get access to the data, please contact the data access committee of the Division of Biomedical Genetics from UMC Utrecht at dacdbg@umcutrecht.nl. Once a data access agreement has been signed and access granted, data can be downloaded using the EGA python client (see detailed instructions at https://github.com/EGA-archive/ega-download-client, and video tutorial at https://embl-ebi.cloud.panopto.eu/Panopto/Pages/Viewer.aspx?id=be79bb93-1737-4f95-b80f-ab4300aa6f5a). Expression matrices in raw counts format and small variants are also publicly available on the github repository under the data folder (permalink https://github.com/IARCbioinfo/MS_panNEN_organoids/tree/9e6005ac1e2bab6777b52ede585ec1b74e244acf/data).

The multiQC report for WGS raw reads is available in Supplementary File S1; the multiQC report for RNA-seq raw reads is available in Supplementary File S2; the multiQC report for WGS alignments is available in Supplementary File S3; the multiQC report for RNA-seq alignments is available in Supplementary File S4.

Organoid lines mentioned in this manuscript can be requested from Hans Clevers (h.clevers@hubrecht.eu) or Talya Dayton (talya.dayton@embl.es). Distribution of organoids to third parties will have to be authorized by the relevant ethical committee and a complete material transfer agreement will be required to ensure compliance with the Dutch 'medical research involving human subjects' act. Use of organoids is subjected to patient consent; note that upon consent withdrawal, distributed organoid lines and any derived material will have to be promptly disposed of.

Abbreviations

bp: base pairs. LCNEC: large-cell neuroendocrine carcinoma. NEC: neuroendocrine carcinoma. NEN: neuroendocrine neoplasms. NET: neuroendocrine tumors. RNA-seq: RNA-sequencing. WGS: whole-genome sequencing. RF: random forest.

Ethical Approval

This study was approved by the medical ethical committee of of each respective hospital of the patients: Verenigde Commissies Mensgebonden Onderzoek of the St. Antonius Hospital Nieuwegein, Z-12.55; UMC Utrecht, METC 12-093 HUB-Cancer; NKI Institutional Review Board (IRB), M18ORG/CFMPB582; Maastricht University Medical Center, METC 2019-1061, and 2019-1039.

Consent for publication

All patients signed informed consent forms for molecular analyses and to the publishing of the data.

Competing Interests

Where authors are identified as personnel of the International Agency for Research on Cancer/World Health Organisation, the authors alone are responsible for the views expressed in this article and they do not necessarily represent the decisions, policy or views of the International Agency for Research on Cancer/World Health Organisation.

H.C.'s full disclosure is given at https://www.uu.nl/staff/JCClevers/. H.C. is inventor of several patents related to organoid technology, cofounder of Xilis Inc. and currently an employee of Roche, Basel.

Funding

The study was funded by the NET Research Foundation (2017 Petersen Accelerator Award to H.C.), Worldwide Cancer Research (2020 Grant Round to L.F-C), NET Research Foundation (2019 Investigator Award to L.F-C), French National Cancer Institute (INCa, PRT-K 2017 to L.F-C. and M.F.), and Ligue Nationale contre le Cancer (fellowship to L.Ma.). T.L.D. was supported by an EMBO long-term fellowship (ALTF-21-2017) and a Marie Skłodowska-Curie IF grant 797966 – PNECtumor. The Oncode Institute is supported by the Dutch Cancer Society.

Author's Contributions

TD designed the study and conducted the experiments. NA and MF designed the bioinformatic workflows. NA performed the data processing. NA and LM performed the analyses. CV formatted and deposited the data on EGA. MF, LFC, and TD supervised the analyses. NA, TD, AS-O, MF, and LFC wrote the manuscript. All authors reviewed and commented the paper.

Acknowledgements

We thank the patients for participating to the study. We thank Utrecht Sequencing for RNA-sequencing services. The results shown here are in part based upon data generated by the Rare Cancers Genomics initiative (www.rarecancersgenomics.com).

References

1. Clevers H. Modeling development and disease with organoids. Cell 2016;165(7):1586–1597.

2. Kim J, Koo BK, Knoblich JA. Human organoids: model systems for human biology and medicine. Nature Reviews Molecular Cell Biology 2020;21(10):571–584.
3. Drost J, Clevers H. Organoids in cancer research. Nature Reviews Cancer 2018;18(7):407.
4. Tuveson D, Clevers H. Cancer modeling meets human organoid technology. Science 2019;364(6444):952–955.
5. LeSavage BL, Suhar RA, Broguiere N, Lutolf MP, Heilshorn SC. Next-generation cancer organoids. Nature materials 2022;21(2):143–159.
6. Dayton T, Alcala N, Moonen L, den Hartig L, Mangiante L, Lap L, et al. Cancer Cell Under review;.
7. Rindi G, Klimstra DS, Abedi-Ardekani B, Asa SL, Bosman FT, Brambilla E, et al. A common classification framework for neuroendocrine neoplasms: an International Agency for Research on Cancer (IARC) and World Health Organization (WHO) expert consensus proposal. Modern Pathology 2018;31(12):1770–1786.
8. Travis W, Beasley M, Cree I, Papotti M, Rekhtman N, et al. Lung neuroendocrine neoplasms. In: WHO Classification of Tumours Editorial Board IARC Press Lyon; 2022.p. 109–111.
9. Klimstra D, Klöppel G, La Rosa S, Rindi G. Classification of neuroendocrine neoplasms of the digestive system. WHO Classification of tumours, 5th Edition Digestive system tumours 2019;p. 16–19.
10. Rudin CM, Poirier JT, Byers LA, Dive C, Dowlati A, George J, et al. Molecular subtypes of small cell lung cancer: a synthesis of human and mouse model data. Nature Reviews Cancer 2019;19(5):289–297.
11. Derks JL, Leblay N, Lantuejoul S, Dingemans AMC, Speel EJM, Fernandez-Cuesta L. New insights into the molecular characteristics of pulmonary carcinoids and large cell neuroendocrine carcinomas, and the impact on their clinical management. Journal of Thoracic Oncology 2018;13(6):752–766.
12. Fernandez-Cuesta L, Foll M. Molecular studies of lung neuroendocrine neoplasms uncover new concepts and entities. Translational Lung Cancer Research 2019;8(Suppl 4):S430.
13. Zhao Z, Chen X, Dowbaj AM, Sljukic A, Bratlie K, Lin L, et al. Organoids. Nature Reviews Methods Primers 2022;2(1):94.
14. Alcala N, Leblay N, Gabriel A, Mangiante L, Hervas D, Giffon T, et al. Integrative and comparative genomic analyses identify clinically relevant pulmonary carcinoid groups and unveil the supra-carcinoids. Nature communications 2019;10.
15. Gabriel AA, Mathian E, Mangiante L, Voegele C, Cahais V, Ghantous A, et al. A molecular map of lung neuroendocrine neoplasms. GigaScience 2020;9(11):giaa112.
16. Di Tommaso P, Chatzou M, Floden EW, Barja PP, Palumbo E, Notredame C. Nextflow enables reproducible computational workflows. Nature biotechnology 2017;35(4):316.
17. Li H, Durbin R. Fast and accurate long-read alignment with Burrows–Wheeler transform. Bioinformatics 2010;26(5):589–595.
18. Vasimuddin M, Misra S, Li H, Aluru S. Efficient architecture-aware acceleration of bwa-mem for multicore systems. In: 2019 IEEE International Parallel and Distributed Processing Symposium (IPDPS) IEEE; 2019. p. 314–324.
19. Faust GG, Hall IM. SAMBLASTER: fast duplicate marking and structural variant read extraction. Bioinformatics 2014;30(17):2503–2505.
20. Tarasov A, Vilella AJ, Cuppen E, Nijman IJ, Prins P. Sambamba: fast processing of NGS alignment formats. Bioinformatics 2015;31(12):2032–2034.
21. Krueger F. Trim Galore: a wrapper tool around Cutadapt and FastQC to consistently apply quality and adapter trimming to FastQ files, with some extra functionality for MspI-digested RRBS-type (Reduced Representation Bisufite-Seq) libraries. URL http://www bioinformatics babraham ac uk/projects/trim_galore/(Date of access: 28/06/2019) 2012;.
22. Martin M. Cutadapt removes adapter sequences from high-throughput sequencing reads. EMBnet journal 2011;17(1):10–12.
23. Dobin A, Davis CA, Schlesinger F, Drenkow J, Zaleski C, Jha S, et al. STAR: ultrafast universal RNA-seq aligner. Bioinformatics 2013;29(1):15–21.
24. Mose LE, Wilkerson MD, Hayes DN, Perou CM, Parker JS. ABRA: improved coding indel detection via assembly-based realignment. Bioinformatics 2014;30(19):2813–2815.
25. Van der Auwera GA, Carneiro MO, Hartl C, Poplin R, Del Angel G, Levy-Moonshine A, et al. From FastQ data to high-confidence variant calls: the genome analysis toolkit best practices pipeline. Current protocols in bioinformatics 2013;43(1):11–10.
26. Benjamin D, Sato T, Cibulskis K, Getz G, Stewart C, Lichtenstein L. Calling somatic SNVs and indels with Mutect2. BioRxiv 2019;p. 861054.
27. Van der Auwera GA, O'Connor BD. Genomics in the cloud: using Docker, GATK, and WDL in Terra. O'Reilly Media; 2020.
28. Danecek P, Bonfield JK, Liddle J, Marshall J, Ohan V, Pollard MO, et al. Twelve years of SAMtools and BCFtools. Gigascience 2021;10(2):giab008.
29. Kim S, Scheffler K, Halpern AL, Bekritsky MA, Noh E, Källberg M, et al. Strelka2: fast and accurate calling of germline and somatic variants. Nature methods 2018;15(8):591–594.
30. Andrews S, Krueger F, Segonds-Pichon A, Biggins L, Krueger C, Wingett S, FastQC. Babraham, UK; 2012. Babraham Institute.
31. Ewels P, Magnusson M, Lundin S, Käller M. MultiQC: summarize analysis results for multiple tools and samples in a single report. Bioinformatics 2016;32(19):3047. +http://dx.doi.org/10.1093/bioinformatics/btw354.
32. Okonechnikov K, Conesa A, García-Alcalde F. Qualimap 2: advanced multi-sample quality control for high-throughput sequencing data. Bioinformatics 2015;32(2):292–294.
33. Wang L, Wang S, Li W. RSeQC: quality control of RNA-seq experiments. Bioinformatics 2012;28(16):2184–2185.
34. Love MI, Huber W, Anders S. Moderated estimation of fold change and dispersion for RNA-seq data with DESeq2. Genome biology 2014;15(12):550.
35. Breiman L. Random forests. Machine learning 2001;45:5–32.
36. Liaw A, Wiener M, et al. Classification and regression by randomForest. R news 2002;2(3):18–22.
37. Di Genova A, Mangiante L, Sexton-Oates A, Voegele C, Fernandez-Cuesta L, Alcala N, et al. A molecular phenotypic map of malignant pleural mesothelioma. GigaScience 2023;12:giac128.
38. Ioannidis NM, Rothstein JH, Pejaver V, Middha S, McDonnell SK, Baheti S, et al. REVEL: an ensemble method for predicting the pathogenicity of rare missense variants. The American Journal of Human Genetics 2016;99(4):877–885.
39. Ishwaran H, Kogalur UB, Gorodeski EZ, Minn AJ, Lauer MS. High-dimensional variable selection for survival data. Journal of the American Statistical Association 2010;105(489):205–217.
40. Banerjee M, Ding Y, Noone AM. Identifying representative trees from ensembles. Statistics in medicine 2012;31(15):1601–1616.
41. Banck MS, Kanwar R, Kulkarni AA, Boora GK, Metge F, Kipp

BR, et al. The genomic landscape of small intestine neuroendocrine tumors. The Journal of clinical investigation 2013;123(6):2502–2508.
42. Sei Y, Zhao X, Forbes J, Szymczak S, Li Q, Trivedi A, et al. A hereditary form of small intestinal carcinoid associated with a germline mutation in inositol polyphosphate multikinase. Gastroenterology 2015;149(1):67–78.
43. Miyoshi T, Umemura S, Matsumura Y, Mimaki S, Tada S, Makinoshima H, et al. Genomic profiling of large-cell neuroendocrine carcinoma of the lung. Clinical Cancer Research 2017;23(3):757–765.
44. Pelosi G, Bianchi F, Dama E, Simbolo M, Mafficini A, Sonzogni A, et al. Most high-grade neuroendocrine tumours of the lung are likely to secondarily develop from pre-existing carcinoids: innovative findings skipping the current pathogenesis paradigm. Virchows Archiv 2018;472:567–577.
45. Simbolo M, Vicentini C, Mafficini A, Fassan M, Pedron S, Corbo V, et al. Mutational and copy number asset of primary sporadic neuroendocrine tumors of the small intestine. Virchows Archiv 2018;473:709–717.
46. Walter D, Harter PN, Battke F, Winkelmann R, Schneider M, Holzer K, et al. Genetic heterogeneity of primary lesion and metastasis in small intestine neuroendocrine tumors. Scientific reports 2018;8(1):3811.
47. Samsom KG, Levy S, van Veenendaal LM, Roepman P, Kodach LL, Steeghs N, et al. Driver mutations occur frequently in metastases of well-differentiated small intestine neuroendocrine tumours. Histopathology 2021;78(4):556–566.
48. Fernandez-Cuesta L, Peifer M, Lu X, Sun R, Ozretić L, Seidel D, et al. Frequent mutations in chromatin-remodelling genes in pulmonary carcinoids. Nature communications 2014;5(1):3518.
49. Mangiante L, Alcala N, Sexton-Oates A, Di Genova A, Gonzalez-Perez A, Khandekar A, et al. Multiomic analysis of malignant pleural mesothelioma identifies molecular axes and specialized tumor profiles driving intertumor heterogeneity. Nature Genetics 2023;55(4):607–618.
50. Lee SH, Hu W, Matulay JT, Silva MV, Owczarek TB, Kim K, et al. Tumor evolution and drug response in patient-derived organoid models of bladder cancer. Cell 2018;173(2):515–528.
51. Laddha SV, Da Silva EM, Robzyk K, Untch BR, Ke H, Rekhtman N, et al. Integrative Genomic Characterization Identifies Molecular Subtypes of Lung CarcinoidsGenomic Analysis Identifies Subtypes of Lung Carcinoids. Cancer research 2019;79(17):4339–4347.
52. Alvarez MJ, Subramaniam PS, Tang LH, Grunn A, Aburi M, Rieckhof G, et al. A precision oncology approach to the pharmacological targeting of mechanistic dependencies in neuroendocrine tumors. Nature genetics 2018;50(7):979–989.
53. Hofving T, Liang F, Karlsson J, Yrlid U, Nilsson JA, Nilsson O, et al. The Microenvironment of Small Intestinal Neuroendocrine Tumours Contains Lymphocytes Capable of Recognition and Activation after Expansion. Cancers 2021;13(17):4305.

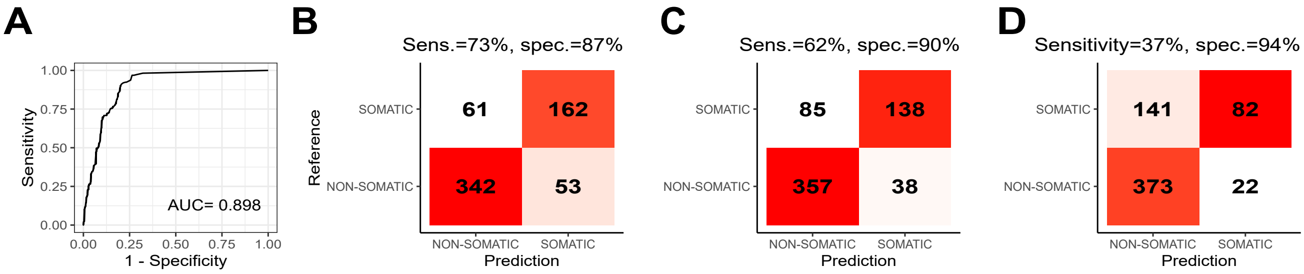

Figure S1. Random forest (RF) classification of variants in genes not reported as driver in neuroendocrine neoplasms. (A) Receiver operating characteristic (ROC) curve. (E)-(G) Confusion matrix for different levels of sensitivity and specificity. Reference: somatic status assessed from whole-genome sequencing data. Prediction: somatic status predicted from RNA-seq data using the RF algorithm.

Click here to access/download
Supplementary Material
supp-1.zip

Click here to access/download
Supplementary Material
response_to_reviews.docx
