# Supplementary material for: Multi-omic dataset of patient-derived tumor organoids of neuroendocrine neoplasms
Source: Gigascience. 2024 Mar 7;13:giae008. doi: 10.1093/gigascience/giae008 (PMC10919335; doi:10.1093/gigascience/giae008)
Supplement: giae008_GIGA-D-23-00277_Revision_2 [file giae008_giga-d-23-00277_revision_2.pdf]

GigaScience

Multi-omic dataset of patient-derived tumor organoids of neuroendocrine neoplasms

--Manuscript Draft--

**Manuscript Number:**	GIGA-D-23-00277R2
**Full Title:**	Multi-omic dataset of patient-derived tumor organoids of neuroendocrine neoplasms
**Article Type:**	Data Note
**Funding Information:**	Neuroendocrine Tumor Research Foundation — Dr Hans Clevers<br>Dr Lynnette Fernandez-Cuesta
	Worldwide Cancer Research — Dr Lynnette Fernandez-Cuesta
	Institut National Du Cancer — Dr Lynnette Fernandez-Cuesta<br>Dr Matthieu Foll
	Ligue Contre le Cancer — Dr Lise Mangiante
	European Molecular Biology Organization — Dr Talya L. Dayton
	H2020 Marie Skłodowska-Curie Actions (797966) — Dr Talya L. Dayton
	KWF Kankerbestrijding — Not applicable
**Abstract:**	Background: Organoids are three-dimensional experimental models that summarize the anatomical and functional structure of an organ. Although a promising experimental model for precision medicine, patient-derived tumor organoids (PDTOs) have currently been developed only for a fraction of tumor types.<br>Results: We have generated the first multi-omic dataset (whole-genome sequencing, WGS, and RNA-sequencing, RNA-seq) of PDTOs from the rare and understudied pulmonary neuroendocrine tumors (n=12; 6 grade 1, 6 grade 2), and provide data from other rare neuroendocrine neoplasms: small intestine (ileal) neuroendocrine tumors (n=6; 2 grade 1 and 4 grade 2) and large-cell neuroendocrine carcinoma (n=5; 1 pancreatic and 4 pulmonary). This dataset includes a matched sample from the parental sample (primary tumor or metastasis) for a majority of samples (21/23) and longitudinal sampling of the PDTOs (1 to 2 time-points), for a total of n=47 RNA-seq and n=33 WGS. We here provide quality control for each technique, and provide the raw and processed data as well as all scripts for genomic analyses to ensure an optimal re-use of the data. In addition, we report gene expression data and somatic small variant calls and describe how they were generated, in particular how we used WGS somatic calls to train a random-forest classifier to detect variants in tumor-only RNA-seq. We also report all histopathological images used for medical diagnosis: hematoxylin and eosin-stained slides, brightfield images, and immunohistochemistry images of protein markers of clinical relevance.<br>Conclusions: This dataset will be critical to future studies relying on this PDTO biobank, such as drug screens for novel therapies and experiments investigating the mechanisms of carcinogenesis in these understudied diseases.
**Corresponding Author:**	Nicolas Alcala<br>International Agency for Research on Cancer<br>Lyon, Rhône-Alpes FRANCE
**Corresponding Author Secondary Information:**	
**Corresponding Author's Institution:**	International Agency for Research on Cancer
**Corresponding Author's Secondary Institution:**	
**First Author:**	Nicolas Alcala
**First Author Secondary Information:**	
**Order of Authors:**	Nicolas Alcala
	Catherine Voegele
	Lise Mangiante

Powered by Editorial Manager® and ProduXion Manager® from Aries Systems Corporation

	Alexandra Sexton-Oates
	Hans Clevers
	Lynnette Fernandez-Cuesta
	Talya L. Dayton
	Matthieu Foll
**Order of Authors Secondary Information:**	
**Response to Reviewers:**	Dear Editor,<br><br>Thank you for the provisional acceptance of our manuscript. Please find attached a revision that follows the journal formatting guidelines.<br><br>Best,<br><br>Nicolas
**Additional Information:**	
**Question**	**Response**
Are you submitting this manuscript to a special series or article collection?	No
**Experimental design and statistics**<br><br>Full details of the experimental design and statistical methods used should be given in the Methods section, as detailed in our Minimum Standards Reporting Checklist. Information essential to interpreting the data presented should be made available in the figure legends.<br><br>Have you included all the information requested in your manuscript?	Yes
**Resources**<br><br>A description of all resources used, including antibodies, cell lines, animals and software tools, with enough information to allow them to be uniquely identified, should be included in the Methods section. Authors are strongly encouraged to cite Research Resource Identifiers (RRIDs) for antibodies, model organisms and tools, where possible.<br><br>Have you included the information requested as detailed in our Minimum Standards Reporting Checklist?	Yes

Powered by Editorial Manager® and ProduXion Manager® from Aries Systems Corporation

**Availability of data and materials**<br><br>All datasets and code on which the conclusions of the paper rely must be either included in your submission or deposited in publicly available repositories (where available and ethically appropriate), referencing such data using a unique identifier in the references and in the “Availability of Data and Materials” section of your manuscript.<br><br>Have you have met the above requirement as detailed in our Minimum Standards Reporting Checklist?	Yes

LaTeX - Main Document (TeX file)
Click here to access/download;LaTeX - Main Document (TeX file);main.tex

```
This is pdfTeX, Version 3.141592653-2.6-1.40.24 (TeX Live 2022)
(preloaded format=pdflatex 2023.3.8)  9 FEB 2024 06:42
entering extended mode
 restricted \write18 enabled.
 %&-line parsing enabled.
**main.tex
(./main.tex
LaTeX2e <2022-11-01> patch level 1
L3 programming layer <2023-02-22> (./oup-contemporary.cls
Document Class: oup-contemporary 2017/06/28, v1.1
(c:/TeXLive/2022/texmf-dist/tex/latex/base/article.cls
Document Class: article 2022/07/02 v1.4n Standard LaTeX document class
(c:/TeXLive/2022/texmf-dist/tex/latex/base/size10.clo
File: size10.clo 2022/07/02 v1.4n Standard LaTeX file (size option)
)
\c@part=\count185
\c@section=\count186
\c@subsection=\count187
\c@subsubsection=\count188
\c@paragraph=\count189
\c@subparagraph=\count190
\c@figure=\count191
\c@table=\count192
\abovecaptionskip=\skip48
\belowcaptionskip=\skip49
\bibindent=\dimen140
) (c:/TeXLive/2022/texmf-dist/tex/latex/base/inputenc.sty
Package: inputenc 2021/02/14 v1.3d Input encoding file
\inpenc@prehook=\toks16
\inpenc@posthook=\toks17
) (c:/TeXLive/2022/texmf-dist/tex/latex/base/fontenc.sty
Package: fontenc 2021/04/29 v2.0v Standard LaTeX package
) (c:/TeXLive/2022/texmf-dist/tex/generic/iftex/ifpdf.sty
Package: ifpdf 2019/10/25 v3.4 ifpdf legacy package. Use iftex instead.
(c:/TeXLive/2022/texmf-dist/tex/generic/iftex/iftex.sty
Package: iftex 2022/02/03 v1.0f TeX engine tests
)) (c:/TeXLive/2022/texmf-dist/tex/latex/microtype/microtype.sty
Package: microtype 2023/03/13 v3.1a Micro-typographical refinements (RS)
(c:/TeXLive/2022/texmf-dist/tex/latex/graphics/keyval.sty
Package: keyval 2022/05/29 v1.15 key=value parser (DPC)
\KV@toks@=\toks18
) (c:/TeXLive/2022/texmf-dist/tex/latex/etoolbox/etoolbox.sty
Package: etoolbox 2020/10/05 v2.5k e-TeX tools for LaTeX (JAW)
\etb@tempcnta=\count193
)
\MT@toks=\toks19
\MT@tempbox=\box51
\MT@count=\count194
LaTeX Info: Redefining \noprotrusionifhmode on input line 1059.
LaTeX Info: Redefining \leftprotrusion on input line 1060.
\MT@prot@toks=\toks20
LaTeX Info: Redefining \rightprotrusion on input line 1078.
LaTeX Info: Redefining \textls on input line 1368.
\MT@outer@kern=\dimen141
```

```
LaTeX Info: Redefining \textmicrotypecontext on input line 1988.
\MT@listname@count=\count195
(c:/TeXLive/2022/texmf-dist/tex/latex/microtype/microtype-pdftex.def
File: microtype-pdftex.def 2023/03/13 v3.1a Definitions specific to
pdftex (RS)

LaTeX Info: Redefining \lsstyle on input line 902.
LaTeX Info: Redefining \lslig on input line 902.
\MT@outer@space=\skip50
)
Package microtype Info: Loading configuration file microtype.cfg.
(c:/TeXLive/2022/texmf-dist/tex/latex/microtype/microtype.cfg
File: microtype.cfg 2023/03/13 v3.1a microtype main configuration file
(RS)
)) (c:/TeXLive/2022/texmf-dist/tex/latex/euler/euler.sty
Package: euler 1995/03/05 v2.5
Package: `euler' v2.5 <1995/03/05> (FJ and FMi)
LaTeX Font Info:    Redeclaring symbol font `letters' on input line 35.
LaTeX Font Info:    Encoding `OML' has changed to `U' for symbol font
(Font)              `letters' in the math version `normal' on input line
35.
LaTeX Font Info:    Overwriting symbol font `letters' in version `normal'
(Font)                  OML/cmm/m/it --> U/eur/m/n on input line 35.
LaTeX Font Info:    Encoding `OML' has changed to `U' for symbol font
(Font)              `letters' in the math version `bold' on input line
35.
LaTeX Font Info:    Overwriting symbol font `letters' in version `bold'
(Font)                  OML/cmm/b/it --> U/eur/m/n on input line 35.
LaTeX Font Info:    Overwriting symbol font `letters' in version `bold'
(Font)                  U/eur/m/n --> U/eur/b/n on input line 36.
LaTeX Font Info:    Redeclaring math symbol \Gamma on input line 47.
LaTeX Font Info:    Redeclaring math symbol \Delta on input line 48.
LaTeX Font Info:    Redeclaring math symbol \Theta on input line 49.
LaTeX Font Info:    Redeclaring math symbol \Lambda on input line 50.
LaTeX Font Info:    Redeclaring math symbol \Xi on input line 51.
LaTeX Font Info:    Redeclaring math symbol \Pi on input line 52.
LaTeX Font Info:    Redeclaring math symbol \Sigma on input line 53.
LaTeX Font Info:    Redeclaring math symbol \Upsilon on input line 54.
LaTeX Font Info:    Redeclaring math symbol \Phi on input line 55.
LaTeX Font Info:    Redeclaring math symbol \Psi on input line 56.
LaTeX Font Info:    Redeclaring math symbol \Omega on input line 57.
\symEulerFraktur=\mathgroup4
LaTeX Font Info:    Overwriting symbol font `EulerFraktur' in version
`bold'
(Font)                  U/euf/m/n --> U/euf/b/n on input line 63.
LaTeX Info: Redefining \oldstylenums on input line 85.
\symEulerScript=\mathgroup5
LaTeX Font Info:    Overwriting symbol font `EulerScript' in version
`bold'
(Font)                  U/eus/m/n --> U/eus/b/n on input line 93.
LaTeX Font Info:    Redeclaring math symbol \aleph on input line 97.
LaTeX Font Info:    Redeclaring math symbol \Re on input line 98.
LaTeX Font Info:    Redeclaring math symbol \Im on input line 99.
LaTeX Font Info:    Redeclaring math delimiter \vert on input line 101.
```

```
LaTeX Font Info:    Redeclaring math delimiter \backslash on input line
103.
LaTeX Font Info:    Redeclaring math symbol \neg on input line 106.
LaTeX Font Info:    Redeclaring math symbol \wedge on input line 108.
LaTeX Font Info:    Redeclaring math symbol \vee on input line 110.
LaTeX Font Info:    Redeclaring math symbol \setminus on input line 112.
LaTeX Font Info:    Redeclaring math symbol \sim on input line 113.
LaTeX Font Info:    Redeclaring math symbol \mid on input line 114.
LaTeX Font Info:    Redeclaring math delimiter \arrowvert on input line
116.
LaTeX Font Info:    Redeclaring math symbol \mathsection on input line
117.
\symEulerExtension=\mathgroup6
LaTeX Font Info:    Redeclaring math symbol \coprod on input line 125.
LaTeX Font Info:    Redeclaring math symbol \prod on input line 125.
LaTeX Font Info:    Redeclaring math symbol \sum on input line 125.
LaTeX Font Info:    Redeclaring math symbol \intop on input line 130.
LaTeX Font Info:    Redeclaring math symbol \ointop on input line 131.
LaTeX Font Info:    Redeclaring math symbol \braceld on input line 132.
LaTeX Font Info:    Redeclaring math symbol \bracerd on input line 133.
LaTeX Font Info:    Redeclaring math symbol \bracelu on input line 134.
LaTeX Font Info:    Redeclaring math symbol \braceru on input line 135.
LaTeX Font Info:    Redeclaring math symbol \infty on input line 136.
LaTeX Font Info:    Redeclaring math symbol \nearrow on input line 153.
LaTeX Font Info:    Redeclaring math symbol \searrow on input line 154.
LaTeX Font Info:    Redeclaring math symbol \nwarrow on input line 155.
LaTeX Font Info:    Redeclaring math symbol \swarrow on input line 156.
LaTeX Font Info:    Redeclaring math symbol \Leftrightarrow on input line
157.
LaTeX Font Info:    Redeclaring math symbol \Leftarrow on input line 158.
LaTeX Font Info:    Redeclaring math symbol \Rightarrow on input line
159.
LaTeX Font Info:    Redeclaring math symbol \leftrightarrow on input line
160.
LaTeX Font Info:    Redeclaring math symbol \leftarrow on input line 161.
LaTeX Font Info:    Redeclaring math symbol \rightarrow on input line
163.
LaTeX Font Info:    Redeclaring math delimiter \uparrow on input line
166.
LaTeX Font Info:    Redeclaring math delimiter \downarrow on input line
168.
LaTeX Font Info:    Redeclaring math delimiter \updownarrow on input line
170.
LaTeX Font Info:    Redeclaring math delimiter \Uparrow on input line
172.
LaTeX Font Info:    Redeclaring math delimiter \Downarrow on input line
174.
LaTeX Font Info:    Redeclaring math delimiter \Updownarrow on input line
176.
LaTeX Font Info:    Redeclaring math symbol \leftharpoonup on input line
177.
LaTeX Font Info:    Redeclaring math symbol \leftharpoondown on input
line 178.
```

```
LaTeX Font Info:    Redeclaring math symbol \rightharpoonup on input line
179.
LaTeX Font Info:    Redeclaring math symbol \rightharpoondown on input
line 180
.
LaTeX Font Info:    Redeclaring math delimiter \lbrace on input line 182.
LaTeX Font Info:    Redeclaring math delimiter \rbrace on input line 184.
\symcmmigroup=\mathgroup7
LaTeX Font Info:    Overwriting symbol font `cmmigroup' in version `bold'
(Font)                  OML/cmm/m/it --> OML/cmm/b/it on input line 200.
LaTeX Font Info:    Redeclaring math accent \vec on input line 201.
LaTeX Font Info:    Redeclaring math symbol \triangleleft on input line
202.
LaTeX Font Info:    Redeclaring math symbol \triangleright on input line
203.
LaTeX Font Info:    Redeclaring math symbol \star on input line 204.
LaTeX Font Info:    Redeclaring math symbol \lhook on input line 205.
LaTeX Font Info:    Redeclaring math symbol \rhook on input line 206.
LaTeX Font Info:    Redeclaring math symbol \flat on input line 207.
LaTeX Font Info:    Redeclaring math symbol \natural on input line 208.
LaTeX Font Info:    Redeclaring math symbol \sharp on input line 209.
LaTeX Font Info:    Redeclaring math symbol \smile on input line 210.
LaTeX Font Info:    Redeclaring math symbol \frown on input line 211.
LaTeX Font Info:    Redeclaring math accent \grave on input line 245.
LaTeX Font Info:    Redeclaring math accent \acute on input line 246.
LaTeX Font Info:    Redeclaring math accent \tilde on input line 247.
LaTeX Font Info:    Redeclaring math accent \ddot on input line 248.
LaTeX Font Info:    Redeclaring math accent \check on input line 249.
LaTeX Font Info:    Redeclaring math accent \breve on input line 250.
LaTeX Font Info:    Redeclaring math accent \bar on input line 251.
LaTeX Font Info:    Redeclaring math accent \dot on input line 252.
LaTeX Font Info:    Redeclaring math accent \hat on input line 254.
) (c:/TeXLive/2022/texmf-dist/tex/latex/merriweather/merriweather.sty
Package: merriweather 2022/09/20 (Bob Tennent) Supports
Merriweather(Sans) font
s for all LaTeX engines.
(c:/TeXLive/2022/texmf-dist/tex/generic/iftex/ifxetex.sty
Package: ifxetex 2019/10/25 v0.7 ifxetex legacy package. Use iftex
instead.
) (c:/TeXLive/2022/texmf-dist/tex/generic/iftex/ifluatex.sty
Package: ifluatex 2019/10/25 v1.5 ifluatex legacy package. Use iftex
instead.
) (c:/TeXLive/2022/texmf-dist/tex/latex/base/textcomp.sty
Package: textcomp 2020/02/02 v2.0n Standard LaTeX package
) (c:/TeXLive/2022/texmf-dist/tex/latex/xkeyval/xkeyval.sty
Package: xkeyval 2022/06/16 v2.9 package option processing (HA)
(c:/TeXLive/2022/texmf-dist/tex/generic/xkeyval/xkeyval.tex
(c:/TeXLive/2022/te
xmf-dist/tex/generic/xkeyval/xkvutils.tex
\XKV@toks=\toks21
\XKV@tempa@toks=\toks22
)
\XKV@depth=\count196
File: xkeyval.tex 2014/12/03 v2.7a key=value parser (HA)
```

```
)) (c:/TeXLive/2022/texmf-dist/tex/latex/base/fontenc.sty
Package: fontenc 2021/04/29 v2.0v Standard LaTeX package
) (c:/TeXLive/2022/texmf-dist/tex/latex/fontaxes/fontaxes.sty
Package: fontaxes 2020/07/21 v1.0e Font selection axes
LaTeX Info: Redefining \upshape on input line 29.
LaTeX Info: Redefining \itshape on input line 31.
LaTeX Info: Redefining \slshape on input line 33.
LaTeX Info: Redefining \swshape on input line 35.
LaTeX Info: Redefining \scshape on input line 37.
LaTeX Info: Redefining \sscshape on input line 39.
LaTeX Info: Redefining \ulcshape on input line 41.
LaTeX Info: Redefining \textsw on input line 47.
LaTeX Info: Redefining \textssc on input line 48.
LaTeX Info: Redefining \textulc on input line 49.
)) (c:/TeXLive/2022/texmf-dist/tex/latex/mathastext/mathastext.sty
Package: mathastext 2022/11/04 v1.3y Use the text font in math mode (JFB)
\mst@exists@muskip=\muskip16
\mst@forall@muskip=\muskip17
\mst@prime@muskip=\muskip18
\mst@do@nonletters=\toks23
\mst@do@easynonletters=\toks24
\mst@do@az=\toks25
\mst@do@AZ=\toks26
\symmtoperatorfont=\mathgroup8
\symmtletterfont=\mathgroup9
** ! and ?
** punctuation: , . : ; and \colon
LaTeX Info: Redefining \relbar on input line 844.
LaTeX Info: Redefining \rightarrowfill on input line 847.
LaTeX Info: Redefining \leftarrowfill on input line 852.
** + and =
LaTeX Info: Redefining \Relbar on input line 943.
** adding = ; and + to \nfss@catcodes
** parentheses ( ) [ ] and slash /
** alldelims: < > \backslash \setminus | \vert \mid \{ and \}
LaTeX Font Info:    Redeclaring math delimiter \backslash on input line
989.
LaTeX Font Info:    Redeclaring math symbol \setminus on input line 1001.
LaTeX Info: Redefining \models on input line 1010.
** \# \mathdollar \% \&
** \imath and \jmath
LaTeX Font Info:    Overwriting math alphabet `\mathnormalbold' in
version `nor
mal'
(Font)                  T1/Merriwthr-OsF/b/it --> T1/Merriwthr-OsF/b/it
on inpu
t line 2370.
LaTeX Font Info:    Overwriting math alphabet `\mathnormalbold' in
version `bol
d'
(Font)                  T1/Merriwthr-OsF/b/it --> T1/Merriwthr-OsF/b/it
on inpu
t line 2370.
```

```
LaTeX Font Info:    Overwriting symbol font `mtletterfont' in version
`normal'
(Font)                  T1/Merriwthr-OsF/m/it --> T1/Merriwthr-OsF/m/it
on inpu
t line 2370.
LaTeX Font Info:    Overwriting symbol font `mtletterfont' in version
`bold'
(Font)                  T1/Merriwthr-OsF/m/it --> T1/Merriwthr-OsF/b/it
on inpu
t line 2370.
LaTeX Font Info:    Overwriting symbol font `mtoperatorfont' in version
`normal
'
(Font)                  T1/Merriwthr-OsF/m/n --> T1/Merriwthr-OsF/m/n on
input
line 2370.
LaTeX Font Info:    Overwriting symbol font `mtoperatorfont' in version
`bold'
(Font)                  T1/Merriwthr-OsF/m/n --> T1/Merriwthr-OsF/b/n on
input
line 2370.
LaTeX Font Info:    Overwriting math alphabet `\Mathbf' in version
`normal'
(Font)                  T1/Merriwthr-OsF/b/n --> T1/Merriwthr-OsF/b/n on
input
line 2370.
LaTeX Font Info:    Overwriting math alphabet `\Mathbf' in version `bold'
(Font)                  T1/Merriwthr-OsF/b/n --> T1/Merriwthr-OsF/b/n on
input
line 2370.
LaTeX Font Info:    Overwriting math alphabet `\Mathit' in version
`normal'
(Font)                  T1/Merriwthr-OsF/m/it --> T1/Merriwthr-OsF/m/it
on inpu
t line 2370.
LaTeX Font Info:    Overwriting math alphabet `\Mathit' in version `bold'
(Font)                  T1/Merriwthr-OsF/m/it --> T1/Merriwthr-OsF/b/it
on inpu
t line 2370.
LaTeX Font Info:    Overwriting math alphabet `\Mathsf' in version
`normal'
(Font)                  T1/MerriwthrSans-OsF/m/n --> T1/MerriwthrSans-
OsF/m/n o
n input line 2370.
LaTeX Font Info:    Overwriting math alphabet `\Mathsf' in version `bold'
(Font)                  T1/MerriwthrSans-OsF/m/n --> T1/MerriwthrSans-
OsF/b/n o
n input line 2370.
LaTeX Font Info:    Overwriting math alphabet `\Mathtt' in version
`normal'
(Font)                  T1/lmtt/m/n --> T1/lmtt/m/n on input line 2370.
LaTeX Font Info:    Overwriting math alphabet `\Mathtt' in version `bold'
(Font)                  T1/lmtt/m/n --> T1/lmtt/b/n on input line 2370.
** Latin letters in the `normal' (resp. `bold') math versions are now
```

```
** set up to use the fonts T1/Merriwthr-OsF/m(b)/it
** Other characters (digits, ...) and \log-like names will be
** typeset with the n shape.
** \hbar
** minus as endash
** \HUGE has been (re)-defined.
** mathastext has declared larger sizes for subscripts.
** To keep LaTeX defaults, use option `defaultmathsizes'.
) (c:/TeXLive/2022/texmf-dist/tex/latex/relsize/relsize.sty
Package: relsize 2013/03/29 ver 4.1
) (c:/TeXLive/2022/texmf-dist/tex/latex/ragged2e/ragged2e.sty
Package: ragged2e 2023/02/25 v3.4 ragged2e Package
\CenteringLeftskip=\skip51
\RaggedLeftLeftskip=\skip52
\RaggedRightLeftskip=\skip53
\CenteringRightskip=\skip54
\RaggedLeftRightskip=\skip55
\RaggedRightRightskip=\skip56
\CenteringParfillskip=\skip57
\RaggedLeftParfillskip=\skip58
\RaggedRightParfillskip=\skip59
\JustifyingParfillskip=\skip60
\CenteringParindent=\skip61
\RaggedLeftParindent=\skip62
\RaggedRightParindent=\skip63
\JustifyingParindent=\skip64
) (c:/TeXLive/2022/texmf-dist/tex/latex/xcolor/xcolor.sty
Package: xcolor 2022/06/12 v2.14 LaTeX color extensions (UK)
(c:/TeXLive/2022/texmf-dist/tex/latex/graphics-cfg/color.cfg
File: color.cfg 2016/01/02 v1.6 sample color configuration
)
Package xcolor Info: Driver file: pdftex.def on input line 227.
(c:/TeXLive/2022/texmf-dist/tex/latex/graphics-def/pdftex.def
File: pdftex.def 2022/09/22 v1.2b Graphics/color driver for pdftex
) (c:/TeXLive/2022/texmf-dist/tex/latex/graphics/mathcolor.ltx)
Package xcolor Info: Model `cmy' substituted by `cmy0' on input line
1353.
Package xcolor Info: Model `hsb' substituted by `rgb' on input line 1357.
Package xcolor Info: Model `RGB' extended on input line 1369.
Package xcolor Info: Model `HTML' substituted by `rgb' on input line
1371.
Package xcolor Info: Model `Hsb' substituted by `hsb' on input line 1372.
Package xcolor Info: Model `tHsb' substituted by `hsb' on input line
1373.
Package xcolor Info: Model `HSB' substituted by `hsb' on input line 1374.
Package xcolor Info: Model `Gray' substituted by `gray' on input line
1375.
Package xcolor Info: Model `wave' substituted by `hsb' on input line
1376.
) (c:/TeXLive/2022/texmf-dist/tex/latex/colortbl/colortbl.sty
Package: colortbl 2022/06/20 v1.0f Color table columns (DPC)
(c:/TeXLive/2022/texmf-dist/tex/latex/tools/array.sty
Package: array 2022/09/04 v2.5g Tabular extension package (FMi)
\col@sep=\dimen142
```

```
\ar@mcellbox=\box52
\extrarowheight=\dimen143
\NC@list=\toks27
\extratabsurround=\skip65
\backup@length=\skip66
\ar@cellbox=\box53
)
\everycr=\toks28
\minrowclearance=\skip67
\rownum=\count197
) (c:/TeXLive/2022/texmf-dist/tex/latex/graphics/graphicx.sty
Package: graphicx 2021/09/16 v1.2d Enhanced LaTeX Graphics (DPC,SPQR)
(c:/TeXLive/2022/texmf-dist/tex/latex/graphics/graphics.sty
Package: graphics 2022/03/10 v1.4e Standard LaTeX Graphics (DPC,SPQR)
(c:/TeXLive/2022/texmf-dist/tex/latex/graphics/trig.sty
Package: trig 2021/08/11 v1.11 sin cos tan (DPC)
) (c:/TeXLive/2022/texmf-dist/tex/latex/graphics-cfg/graphics.cfg
File: graphics.cfg 2016/06/04 v1.11 sample graphics configuration
)
Package graphics Info: Driver file: pdftex.def on input line 107.
)
\Gin@req@height=\dimen144
\Gin@req@width=\dimen145
) (c:/TeXLive/2022/texmf-dist/tex/latex/xpatch/xpatch.sty
(c:/TeXLive/2022/texm
f-dist/tex/latex/l3kernel/expl3.sty
Package: expl3 2023-02-22 L3 programming layer (loader)
(c:/TeXLive/2022/texmf-dist/tex/latex/l3backend/l3backend-pdftex.def
File: l3backend-pdftex.def 2023-01-16 L3 backend support: PDF output
(pdfTeX)
\l__color_backend_stack_int=\count198
\l__pdf_internal_box=\box54
))
Package: xpatch 2020/03/25 v0.3a Extending etoolbox patching commands
(c:/TeXLive/2022/texmf-dist/tex/latex/l3packages/xparse/xparse.sty
Package: xparse 2023-02-02 L3 Experimental document command parser
)) (c:/TeXLive/2022/texmf-dist/tex/latex/environ/environ.sty
Package: environ 2014/05/04 v0.3 A new way to define environments
(c:/TeXLive/2022/texmf-dist/tex/latex/trimspaces/trimspaces.sty
Package: trimspaces 2009/09/17 v1.1 Trim spaces around a token list
)
\@envbody=\toks29
) (c:/TeXLive/2022/texmf-dist/tex/latex/lastpage/lastpage.sty
Package: lastpage 2023/03/07 v2.0a lastpage: 2.09 or 2e? (HMM)
(c:/TeXLive/2022/texmf-dist/tex/latex/lastpage/lastpage2e.sty
Package: lastpage2e 2023/03/07 v2.0a Decide which 2e lastpage version to
use (H
MM)
(c:/TeXLive/2022/texmf-dist/tex/latex/lastpage/lastpagemodern.sty
Package: lastpagemodern 2023-03-07 v2.0a Refers to last page's name (HMM;
JPG)
)
)) (c:/TeXLive/2022/texmf-dist/tex/latex/graphics/rotating.sty
Package: rotating 2016/08/11 v2.16d rotated objects in LaTeX
```

```
(c:/TeXLive/2022/texmf-dist/tex/latex/base/ifthen.sty
Package: ifthen 2022/04/13 v1.1d Standard LaTeX ifthen package (DPC)
)
\c@r@tfl@t=\count199
\rotFPtop=\skip68
\rotFPbot=\skip69
\rot@float@box=\box55
\rot@mess@toks=\toks30
) (c:/TeXLive/2022/texmf-dist/tex/latex/graphics/lscape.sty
Package: lscape 2020/05/28 v3.02 Landscape Pages (DPC)
) (c:/TeXLive/2022/texmf-dist/tex/latex/tools/afterpage.sty
Package: afterpage 2014/10/28 v1.08 After-Page Package (DPC)
\AP@output=\toks31
\AP@partial=\box56
\AP@footins=\box57
) (c:/TeXLive/2022/texmf-dist/tex/latex/textpos/textpos.sty
Package: textpos 2022/07/23 v1.10.1
Package textpos Info: choosing support for LaTeX3 on input line 60.
\TP@textbox=\box58
\TP@holdbox=\box59
\TPHorizModule=\dimen146
\TPVertModule=\dimen147
\TP@margin=\dimen148
\TP@absmargin=\dimen149
Grid set 16 x 16 = 37.34424pt x 52.81541pt
\TPboxrulesize=\dimen150
\TP@ox=\dimen151
\TP@oy=\dimen152
\TP@tbargs=\toks32
TextBlockOrigin set to 0pt x 0pt
) (c:/TeXLive/2022/texmf-dist/tex/latex/url/url.sty
\Urlmuskip=\muskip19
Package: url 2013/09/16  ver 3.4  Verb mode for urls, etc.
) (c:/TeXLive/2022/texmf-dist/tex/latex/newfloat/newfloat.sty
Package: newfloat 2019/09/02 v1.1l Defining new floating environments
(AR)
Package newfloat Info: `rotating' package detected.
) (c:/TeXLive/2022/texmf-dist/tex/latex/mdframed/mdframed.sty
Package: mdframed 2013/07/01 1.9b: mdframed
(c:/TeXLive/2022/texmf-dist/tex/latex/kvoptions/kvoptions.sty
Package: kvoptions 2022-06-15 v3.15 Key value format for package options
(HO)
(c:/TeXLive/2022/texmf-dist/tex/generic/ltxcmds/ltxcmds.sty
Package: ltxcmds 2020-05-10 v1.25 LaTeX kernel commands for general use
(HO)
) (c:/TeXLive/2022/texmf-dist/tex/latex/kvsetkeys/kvsetkeys.sty
Package: kvsetkeys 2022-10-05 v1.19 Key value parser (HO)
)) (c:/TeXLive/2022/texmf-dist/tex/latex/zref/zref-abspage.sty
Package: zref-abspage 2022-04-07 v2.34 Module abspage for zref (HO)
(c:/TeXLive/2022/texmf-dist/tex/latex/zref/zref-base.sty
Package: zref-base 2022-04-07 v2.34 Module base for zref (HO)
(c:/TeXLive/2022/texmf-dist/tex/generic/infwarerr/infwarerr.sty
Package: infwarerr 2019/12/03 v1.5 Providing info/warning/error messages
(HO)
```

```
) (c:/TeXLive/2022/texmf-dist/tex/generic/kvdefinekeys/kvdefinekeys.sty
Package: kvdefinekeys 2019-12-19 v1.6 Define keys (HO)
) (c:/TeXLive/2022/texmf-dist/tex/generic/pdftexcmds/pdftexcmds.sty
Package: pdftexcmds 2020-06-27 v0.33 Utility functions of pdfTeX for
LuaTeX (HO
)
Package pdftexcmds Info: \pdf@primitive is available.
Package pdftexcmds Info: \pdf@ifprimitive is available.
Package pdftexcmds Info: \pdfdraftmode found.
) (c:/TeXLive/2022/texmf-dist/tex/generic/etexcmds/etexcmds.sty
Package: etexcmds 2019/12/15 v1.7 Avoid name clashes with e-TeX commands
(HO)
) (c:/TeXLive/2022/texmf-dist/tex/latex/auxhook/auxhook.sty
Package: auxhook 2019-12-17 v1.6 Hooks for auxiliary files (HO)
)
Package zref Info: New property list: main on input line 767.
Package zref Info: New property: default on input line 768.
Package zref Info: New property: page on input line 769.
) (c:/TeXLive/2022/texmf-dist/tex/latex/base/atbegshi-ltx.sty
Package: atbegshi-ltx 2021/01/10 v1.0c Emulation of the original atbegshi
package with kernel methods
)
\c@abspage=\count266
Package zref Info: New property: abspage on input line 65.
) (c:/TeXLive/2022/texmf-dist/tex/latex/needspace/needspace.sty
Package: needspace 2010/09/12 v1.3d reserve vertical space
)
\mdf@templength=\skip70
\c@mdf@globalstyle@cnt=\count267
\mdf@skipabove@length=\skip71
\mdf@skipbelow@length=\skip72
\mdf@leftmargin@length=\skip73
\mdf@rightmargin@length=\skip74
\mdf@innerleftmargin@length=\skip75
\mdf@innerrightmargin@length=\skip76
\mdf@innertopmargin@length=\skip77
\mdf@innerbottommargin@length=\skip78
\mdf@splittopskip@length=\skip79
\mdf@splitbottomskip@length=\skip80
\mdf@outermargin@length=\skip81
\mdf@innermargin@length=\skip82
\mdf@linewidth@length=\skip83
\mdf@innerlinewidth@length=\skip84
\mdf@middlelinewidth@length=\skip85
\mdf@outerlinewidth@length=\skip86
\mdf@roundcorner@length=\skip87
\mdf@footenotedistance@length=\skip88
\mdf@userdefinedwidth@length=\skip89
\mdf@needspace@length=\skip90
\mdf@frametitleaboveskip@length=\skip91
\mdf@frametitlebelowskip@length=\skip92
\mdf@frametitlerulewidth@length=\skip93
\mdf@frametitleleftmargin@length=\skip94
\mdf@frametitlerightmargin@length=\skip95
```

```
\mdf@shadowsize@length=\skip96
\mdf@extratopheight@length=\skip97
\mdf@subtitleabovelinewidth@length=\skip98
\mdf@subtitlebelowlinewidth@length=\skip99
\mdf@subtitleaboveskip@length=\skip100
\mdf@subtitlebelowskip@length=\skip101
\mdf@subtitleinneraboveskip@length=\skip102
\mdf@subtitleinnerbelowskip@length=\skip103
\mdf@subsubtitleabovelinewidth@length=\skip104
\mdf@subsubtitlebelowlinewidth@length=\skip105
\mdf@subsubtitleaboveskip@length=\skip106
\mdf@subsubtitlebelowskip@length=\skip107
\mdf@subsubtitleinneraboveskip@length=\skip108
\mdf@subsubtitleinnerbelowskip@length=\skip109
(c:/TeXLive/2022/texmf-dist/tex/latex/mdframed/md-frame-0.mdf
File: md-frame-0.mdf 2013/07/01\ 1.9b: md-frame-0
)
\mdf@frametitlebox=\box60
\mdf@footnotebox=\box61
\mdf@splitbox@one=\box62
\mdf@splitbox@two=\box63
\mdf@splitbox@save=\box64
\mdfsplitboxwidth=\skip110
\mdfsplitboxtotalwidth=\skip111
\mdfsplitboxheight=\skip112
\mdfsplitboxdepth=\skip113
\mdfsplitboxtotalheight=\skip114
\mdfframetitleboxwidth=\skip115
\mdfframetitleboxtotalwidth=\skip116
\mdfframetitleboxheight=\skip117
\mdfframetitleboxdepth=\skip118
\mdfframetitleboxtotalheight=\skip119
\mdffootnoteboxwidth=\skip120
\mdffootnoteboxtotalwidth=\skip121
\mdffootnoteboxheight=\skip122
\mdffootnoteboxdepth=\skip123
\mdffootnoteboxtotalheight=\skip124
\mdftotallinewidth=\skip125
\mdfboundingboxwidth=\skip126
\mdfboundingboxtotalwidth=\skip127
\mdfboundingboxheight=\skip128
\mdfboundingboxdepth=\skip129
\mdfboundingboxtotalheight=\skip130
\mdf@freevspace@length=\skip131
\mdf@horizontalwidthofbox@length=\skip132
\mdf@verticalmarginwhole@length=\skip133
\mdf@horizontalspaceofbox=\skip134
\mdfsubtitleheight=\skip135
\mdfsubsubtitleheight=\skip136
\c@mdfcountframes=\count268

****** mdframed patching \endmdf@trivlist

****** -- success******
```

```
\mdf@envdepth=\count269
\c@mdf@env@i=\count270
\c@mdf@env@ii=\count271
\c@mdf@zref@counter=\count272
Package zref Info: New property: mdf@pagevalue on input line 895.
) (c:/TeXLive/2022/texmf-dist/tex/latex/titlesec/titlesec.sty
Package: titlesec 2021/07/05 v2.14 Sectioning titles
\ttl@box=\box65
\beforetitleunit=\skip137
\aftertitleunit=\skip138
\ttl@plus=\dimen153
\ttl@minus=\dimen154
\ttl@toksa=\toks33
\titlewidth=\dimen155
\titlewidthlast=\dimen156
\titlewidthfirst=\dimen157
) (c:/TeXLive/2022/texmf-dist/tex/latex/koma-script/scrextend.sty
Package: scrextend 2022/10/12 v3.38 KOMA-Script package (extend other
classes w
ith features of KOMA-Script classes)
(c:/TeXLive/2022/texmf-dist/tex/latex/koma-script/scrkbase.sty
Package: scrkbase 2022/10/12 v3.38 KOMA-Script package (KOMA-Script-
dependent b
asics and keyval usage)
(c:/TeXLive/2022/texmf-dist/tex/latex/koma-script/scrbase.sty
Package: scrbase 2022/10/12 v3.38 KOMA-Script package (KOMA-Script-
independent
basics and keyval usage)
(c:/TeXLive/2022/texmf-dist/tex/latex/koma-script/scrlfile.sty
Package: scrlfile 2022/10/12 v3.38 KOMA-Script package (file load hooks)
(c:/TeXLive/2022/texmf-dist/tex/latex/koma-script/scrlfile-hook.sty
Package: scrlfile-hook 2022/10/12 v3.38 KOMA-Script package (using LaTeX
hooks)

(c:/TeXLive/2022/texmf-dist/tex/latex/koma-script/scrlogo.sty
Package: scrlogo 2022/10/12 v3.38 KOMA-Script package (logo)
)))
Applying: [2021/05/01] Usage of raw or classic option list on input line
252.
Already applied: [0000/00/00] Usage of raw or classic option list on
input line
 368.
))
Package scrextend Info: unexpected definition of `\@makefnmark'.
(scrextend)             Trying to patch it on input line 1709.
Package scrextend Info: patch seems to be successfull on input line 1709.
)

LaTeX Font Warning: Font shape `T1/cmr/m/n' in size <7.5> not available
(Font)              size <7> substituted on input line 65.

(c:/TeXLive/2022/texmf-dist/tex/latex/tools/calc.sty
Package: calc 2017/05/25 v4.3 Infix arithmetic (KKT,FJ)
```

```
\calc@Acount=\count273
\calc@Bcount=\count274
\calc@Adimen=\dimen158
\calc@Bdimen=\dimen159
\calc@Askip=\skip139
\calc@Bskip=\skip140
LaTeX Info: Redefining \setlength on input line 80.
LaTeX Info: Redefining \addtolength on input line 81.
\calc@Ccount=\count275
\calc@Cskip=\skip141
) (c:/TeXLive/2022/texmf-dist/tex/latex/geometry/geometry.sty
Package: geometry 2020/01/02 v5.9 Page Geometry
(c:/TeXLive/2022/texmf-dist/tex/generic/iftex/ifvtex.sty
Package: ifvtex 2019/10/25 v1.7 ifvtex legacy package. Use iftex instead.
)
\Gm@cnth=\count276
\Gm@cntv=\count277
\c@Gm@tempcnt=\count278
\Gm@bindingoffset=\dimen160
\Gm@wd@mp=\dimen161
\Gm@odd@mp=\dimen162
\Gm@even@mp=\dimen163
\Gm@layoutwidth=\dimen164
\Gm@layoutheight=\dimen165
\Gm@layouthoffset=\dimen166
\Gm@layoutvoffset=\dimen167
\Gm@dimlist=\toks34
) (c:/TeXLive/2022/texmf-dist/tex/latex/hyperref/hyperref.sty
Package: hyperref 2023-02-07 v7.00v Hypertext links for LaTeX
(c:/TeXLive/2022/texmf-dist/tex/generic/pdfescape/pdfescape.sty
Package: pdfescape 2019/12/09 v1.15 Implements pdfTeX's escape features
(HO)
) (c:/TeXLive/2022/texmf-dist/tex/latex/hycolor/hycolor.sty
Package: hycolor 2020-01-27 v1.10 Color options for hyperref/bookmark
(HO)
) (c:/TeXLive/2022/texmf-dist/tex/latex/letltxmacro/letltxmacro.sty
Package: letltxmacro 2019/12/03 v1.6 Let assignment for LaTeX macros (HO)
) (c:/TeXLive/2022/texmf-dist/tex/latex/hyperref/nameref.sty
Package: nameref 2022-05-17 v2.50 Cross-referencing by name of section
(c:/TeXLive/2022/texmf-dist/tex/latex/refcount/refcount.sty
Package: refcount 2019/12/15 v3.6 Data extraction from label references
(HO)
) (c:/TeXLive/2022/texmf-
dist/tex/generic/gettitlestring/gettitlestring.sty
Package: gettitlestring 2019/12/15 v1.6 Cleanup title references (HO)
)
\c@section@level=\count279
)
\@linkdim=\dimen168
\Hy@linkcounter=\count280
\Hy@pagecounter=\count281
(c:/TeXLive/2022/texmf-dist/tex/latex/hyperref/pd1enc.def
File: pd1enc.def 2023-02-07 v7.00v Hyperref: PDFDocEncoding definition
(HO)
```

```
Now handling font encoding PD1 ...
... no UTF-8 mapping file for font encoding PD1
) (c:/TeXLive/2022/texmf-dist/tex/generic/intcalc/intcalc.sty
Package: intcalc 2019/12/15 v1.3 Expandable calculations with integers
(HO)
)
\Hy@SavedSpaceFactor=\count282
(c:/TeXLive/2022/texmf-dist/tex/latex/hyperref/puenc.def
File: puenc.def 2023-02-07 v7.00v Hyperref: PDF Unicode definition (HO)
Now handling font encoding PU ...
... no UTF-8 mapping file for font encoding PU
)
Package hyperref Info: Option `colorlinks' set `true' on input line 4060.
Package hyperref Info: Hyper figures OFF on input line 4177.
Package hyperref Info: Link nesting OFF on input line 4182.
Package hyperref Info: Hyper index ON on input line 4185.
Package hyperref Info: Plain pages OFF on input line 4192.
Package hyperref Info: Backreferencing OFF on input line 4197.
Package hyperref Info: Implicit mode ON; LaTeX internals redefined.
Package hyperref Info: Bookmarks ON on input line 4425.
\c@Hy@tempcnt=\count283
LaTeX Info: Redefining \url on input line 4763.
\XeTeXLinkMargin=\dimen169
(c:/TeXLive/2022/texmf-dist/tex/generic/bitset/bitset.sty
Package: bitset 2019/12/09 v1.3 Handle bit-vector datatype (HO)
(c:/TeXLive/2022/texmf-dist/tex/generic/bigintcalc/bigintcalc.sty
Package: bigintcalc 2019/12/15 v1.5 Expandable calculations on big
integers (HO
)
))
\Fld@menulength=\count284
\Field@Width=\dimen170
\Fld@charsize=\dimen171
Package hyperref Info: Hyper figures OFF on input line 6042.
Package hyperref Info: Link nesting OFF on input line 6047.
Package hyperref Info: Hyper index ON on input line 6050.
Package hyperref Info: backreferencing OFF on input line 6057.
Package hyperref Info: Link coloring ON on input line 6060.
Package hyperref Info: Link coloring with OCG OFF on input line 6067.
Package hyperref Info: PDF/A mode OFF on input line 6072.
\Hy@abspage=\count285
\c@Item=\count286
\c@Hfootnote=\count287
)
Package hyperref Info: Driver (autodetected): hpdftex.
(c:/TeXLive/2022/texmf-dist/tex/latex/hyperref/hpdftex.def
File: hpdftex.def 2023-02-07 v7.00v Hyperref driver for pdfTeX
(c:/TeXLive/2022/texmf-dist/tex/latex/base/atveryend-ltx.sty
Package: atveryend-ltx 2020/08/19 v1.0a Emulation of the original
atveryend pac
kage
with kernel methods
)
\HyAnn@Count=\count288
```

```
\Fld@listcount=\count289
\c@bookmark@seq@number=\count290
(c:/TeXLive/2022/texmf-dist/tex/latex/rerunfilecheck/rerunfilecheck.sty
Package: rerunfilecheck 2022-07-10 v1.10 Rerun checks for auxiliary files
(HO)
(c:/TeXLive/2022/texmf-dist/tex/generic/uniquecounter/uniquecounter.sty
Package: uniquecounter 2019/12/15 v1.4 Provide unlimited unique counter
(HO)
)
Package uniquecounter Info: New unique counter `rerunfilecheck' on input
line 2
85.
)
\Hy@SectionHShift=\skip142
) (c:/TeXLive/2022/texmf-dist/tex/latex/preprint/authblk.sty
Package: authblk 2001/02/27 1.3 (PWD)
\affilsep=\skip143
\@affilsep=\skip144
\c@Maxaffil=\count291
\c@authors=\count292
\c@affil=\count293
) (c:/TeXLive/2022/texmf-dist/tex/latex/footmisc/footmisc.sty
Package: footmisc 2022/03/08 v6.0d a miscellany of footnote facilities
\FN@temptoken=\toks35
\footnotemargin=\dimen172
\@outputbox@depth=\dimen173
Package footmisc Info: Declaring symbol style bringhurst on input line
695.
Package footmisc Info: Declaring symbol style chicago on input line 703.
Package footmisc Info: Declaring symbol style wiley on input line 712.
Package footmisc Info: Declaring symbol style lamport-robust on input
line 723.

Package footmisc Info: Declaring symbol style lamport* on input line 743.
Package footmisc Info: Declaring symbol style lamport*-robust on input
line 764
.
) (c:/TeXLive/2022/texmf-dist/tex/latex/fancyhdr/fancyhdr.sty
Package: fancyhdr 2022/11/09 v4.1 Extensive control of page headers and
footers

\f@nch@headwidth=\skip145
\f@nch@O@elh=\skip146
\f@nch@O@erh=\skip147
\f@nch@O@olh=\skip148
\f@nch@O@orh=\skip149
\f@nch@O@elf=\skip150
\f@nch@O@erf=\skip151
\f@nch@O@olf=\skip152
\f@nch@O@orf=\skip153
) (c:/TeXLive/2022/texmf-dist/tex/generic/alphalph/alphalph.sty
Package: alphalph 2019/12/09 v2.6 Convert numbers to letters (HO)
)
\c@authorfn=\count294
```

```
(c:/TeXLive/2022/texmf-dist/tex/latex/abstract/abstract.sty
Package: abstract 2009/06/08 v1.2a configurable abstracts
\abstitleskip=\skip154
\absleftindent=\skip155
\absrightindent=\skip156
\absparindent=\skip157
\absparsep=\skip158
)
Package newfloat Info: New float `keypoints' with options
`placement=t!,name=kp
t' on input line 296.
\c@keypoints=\count295
\newfloat@ftype=\count296
Package newfloat Info: float type `keypoints'=8 on input line 296.
(c:/TeXLive/2022/texmf-dist/tex/latex/enumitem/enumitem.sty
Package: enumitem 2019/06/20 v3.9 Customized lists
\labelindent=\skip159
\enit@outerparindent=\dimen174
\enit@toks=\toks36
\enit@inbox=\box66
\enit@count@id=\count297
\enitdp@description=\count298
) (c:/TeXLive/2022/texmf-dist/tex/latex/quoting/quoting.sty
Package: quoting 2014/01/28 v0.1c Consolidated environment for displayed
text
\quo@toppartop=\skip160
) (c:/TeXLive/2022/texmf-dist/tex/latex/sttools/stfloats.sty
Package: stfloats 2017/03/27 v3.3 Improve float mechanism and
baselineskip sett
ings
\@dblbotnum=\count299
\c@dblbotnumber=\count300
) (c:/TeXLive/2022/texmf-dist/tex/latex/booktabs/booktabs.sty
Package: booktabs 2020/01/12 v1.61803398 Publication quality tables
\heavyrulewidth=\dimen175
\lightrulewidth=\dimen176
\cmidrulewidth=\dimen177
\belowrulesep=\dimen178
\belowbottomsep=\dimen179
\aboverulesep=\dimen180
\abovetopsep=\dimen181
\cmidrulesep=\dimen182
\cmidrulekern=\dimen183
\defaultaddspace=\dimen184
\@cmidla=\count301
\@cmidlb=\count302
\@aboverulesep=\dimen185
\@belowrulesep=\dimen186
\@thisruleclass=\count303
\@lastruleclass=\count304
\@thisrulewidth=\dimen187
) (c:/TeXLive/2022/texmf-dist/tex/latex/tools/tabularx.sty
Package: tabularx 2020/01/15 v2.11c `tabularx' package (DPC)
\TX@col@width=\dimen188
```

```
\TX@old@table=\dimen189
\TX@old@col=\dimen190
\TX@target=\dimen191
\TX@delta=\dimen192
\TX@cols=\count305
\TX@ftn=\toks37
)
\enitdp@tablenotes=\count306
(c:/TeXLive/2022/texmf-dist/tex/latex/caption/caption.sty
Package: caption 2022/03/01 v3.6b Customizing captions (AR)
(c:/TeXLive/2022/texmf-dist/tex/latex/caption/caption3.sty
Package: caption3 2022/03/17 v2.3b caption3 kernel (AR)
\caption@tempdima=\dimen193
\captionmargin=\dimen194
\caption@leftmargin=\dimen195
\caption@rightmargin=\dimen196
\caption@width=\dimen197
\caption@indent=\dimen198
\caption@parindent=\dimen199
\caption@hangindent=\dimen256
Package caption Info: Standard document class detected.
)
\c@caption@flags=\count307
\c@continuedfloat=\count308
Package caption Info: hyperref package is loaded.
Package caption Info: rotating package is loaded.
) (c:/TeXLive/2022/texmf-dist/tex/latex/natbib/natbib.sty
Package: natbib 2010/09/13 8.31b (PWD, AO)
\bibhang=\skip161
\bibsep=\skip162
LaTeX Info: Redefining \cite on input line 694.
\c@NAT@ctr=\count309
)) (c:/TeXLive/2022/texmf-dist/tex/latex/siunitx/siunitx.sty
Package: siunitx 2023-03-04 v3.2.2 A comprehensive (SI) units package
\l__siunitx_angle_tmp_dim=\dimen257
\l__siunitx_angle_marker_box=\box67
\l__siunitx_angle_unit_box=\box68
\l__siunitx_compound_count_int=\count310
(c:/TeXLive/2022/texmf-dist/tex/latex/translations/translations.sty
Package: translations 2022/02/05 v1.12 internationalization of LaTeX2e
packages
 (CN)
)
\l__siunitx_number_exponent_fixed_int=\count311
\l__siunitx_number_min_decimal_int=\count312
\l__siunitx_number_min_integer_int=\count313
\l__siunitx_number_round_precision_int=\count314
\l__siunitx_number_lower_threshold_int=\count315
\l__siunitx_number_upper_threshold_int=\count316
\l__siunitx_number_group_first_int=\count317
\l__siunitx_number_group_size_int=\count318
\l__siunitx_number_group_minimum_int=\count319
(c:/TeXLive/2022/texmf-dist/tex/latex/amsmath/amstext.sty
Package: amstext 2021/08/26 v2.01 AMS text
```

```
(c:/TeXLive/2022/texmf-dist/tex/latex/amsmath/amsgen.sty
File: amsgen.sty 1999/11/30 v2.0 generic functions
\@emptytoks=\toks38
\ex@=\dimen258
))
\l__siunitx_table_tmp_box=\box69
\l__siunitx_table_tmp_dim=\dimen259
\l__siunitx_table_column_width_dim=\dimen260
\l__siunitx_table_integer_box=\box70
\l__siunitx_table_decimal_box=\box71
\l__siunitx_table_uncert_box=\box72
\l__siunitx_table_before_box=\box73
\l__siunitx_table_after_box=\box74
\l__siunitx_table_before_dim=\dimen261
\l__siunitx_table_carry_dim=\dimen262
\l__siunitx_unit_tmp_int=\count320
\l__siunitx_unit_position_int=\count321
\l__siunitx_unit_total_int=\count322
)

Package array Warning: Redefining primitive column b on input line 19.

Package translations Info: No language package found. I am going to use
`englis
h' as default language. on input line 58.
LaTeX Font Info:    Trying to load font information for T1+Merriwthr-OsF
on inp
ut line 58.
(c:/TeXLive/2022/texmf-dist/tex/latex/merriweather/T1Merriwthr-OsF.fd
File: T1Merriwthr-OsF.fd 2020/08/30 (autoinst) Font definitions for
T1/Merriwth
r-OsF.
)
LaTeX Font Info:    Font shape `T1/Merriwthr-OsF/m/n' will be
(Font)              scaled to size 7.5pt on input line 58.
(./main.aux)
\openout1 = `main.aux'.

LaTeX Font Info:    Checking defaults for OML/cmm/m/it on input line 58.
LaTeX Font Info:    ... okay on input line 58.
LaTeX Font Info:    Checking defaults for OMS/cmsy/m/n on input line 58.
LaTeX Font Info:    ... okay on input line 58.
LaTeX Font Info:    Checking defaults for OT1/cmr/m/n on input line 58.
LaTeX Font Info:    ... okay on input line 58.
LaTeX Font Info:    Checking defaults for T1/cmr/m/n on input line 58.
LaTeX Font Info:    ... okay on input line 58.
LaTeX Font Info:    Checking defaults for TS1/cmr/m/n on input line 58.
LaTeX Font Info:    ... okay on input line 58.
LaTeX Font Info:    Checking defaults for OMX/cmex/m/n on input line 58.
LaTeX Font Info:    ... okay on input line 58.
LaTeX Font Info:    Checking defaults for U/cmr/m/n on input line 58.
LaTeX Font Info:    ... okay on input line 58.
LaTeX Font Info:    Checking defaults for PD1/pdf/m/n on input line 58.
LaTeX Font Info:    ... okay on input line 58.
```

```
LaTeX Font Info:    Checking defaults for PU/pdf/m/n on input line 58.
LaTeX Font Info:    ... okay on input line 58.
LaTeX Info: Redefining \microtypecontext on input line 58.
Package microtype Info: Applying patch `item' on input line 58.
Package microtype Info: Applying patch `toc' on input line 58.
Package microtype Info: Applying patch `eqnum' on input line 58.

Package microtype Warning: Unable to apply patch `footnote' on input line
58.

Package microtype Info: Applying patch `verbatim' on input line 58.
Package microtype Info: Generating PDF output.
Package microtype Info: Character protrusion enabled (level 2).
Package microtype Info: Using default protrusion set `alltext'.
Package microtype Info: Automatic font expansion enabled (level 2),
(microtype)             stretch: 20, shrink: 20, step: 1, non-selected.
Package microtype Info: Using default expansion set `alltext-nott'.
LaTeX Info: Redefining \showhyphens on input line 58.
Package microtype Info: No adjustment of tracking.
Package microtype Info: No adjustment of interword spacing.
Package microtype Info: No adjustment of character kerning.
Package microtype Info: Loading generic protrusion settings for font
family
(microtype)             `Merriwthr-OsF' (encoding: T1).
(microtype)             For optimal results, create family-specific
settings.
(microtype)             See the microtype manual for details.
LaTeX Font Info:    Redeclaring symbol font `operators' on input line 58.
LaTeX Font Info:    Encoding `OT1' has changed to `T1' for symbol font
(Font)              `operators' in the math version `normal' on input
line 58.
LaTeX Font Info:    Overwriting symbol font `operators' in version
`normal'
(Font)                  OT1/cmr/m/n --> T1/Merriwthr-OsF/m/up on input
line 58.

LaTeX Font Info:    Encoding `OT1' has changed to `T1' for symbol font
(Font)              `operators' in the math version `bold' on input line
58.
LaTeX Font Info:    Overwriting symbol font `operators' in version `bold'
(Font)                  OT1/cmr/bx/n --> T1/Merriwthr-OsF/m/up on input
line 58
.
LaTeX Font Info:    Overwriting symbol font `operators' in version `bold'
(Font)                  T1/Merriwthr-OsF/m/up --> T1/Merriwthr-OsF/b/up
on inpu
t line 58.
LaTeX Font Info:    Redeclaring math alphabet \mathbf on input line 58.
LaTeX Font Info:    Overwriting math alphabet `\mathbf' in version
`normal'
(Font)                  OT1/cmr/bx/n --> T1/Merriwthr-OsF/b/up on input
line 58
.
LaTeX Font Info:    Overwriting math alphabet `\mathbf' in version `bold'
```

```
(Font)              OT1/cmr/bx/n --> T1/Merriwthr-OsF/b/up on input
line 58
.
LaTeX Font Info:    Redeclaring math alphabet \mathsf on input line 58.
LaTeX Font Info:    Overwriting math alphabet `\mathsf' in version
`normal'
(Font)              OT1/cmss/m/n --> T1/MerriwthrSans-OsF/m/up on
input lin
e 58.
LaTeX Font Info:    Overwriting math alphabet `\mathsf' in version `bold'
(Font)              OT1/cmss/bx/n --> T1/MerriwthrSans-OsF/m/up on
input li
ne 58.
LaTeX Font Info:    Redeclaring math alphabet \mathit on input line 58.
LaTeX Font Info:    Overwriting math alphabet `\mathit' in version
`normal'
(Font)              OT1/cmr/m/it --> T1/Merriwthr-OsF/m/it on input
line 58
.
LaTeX Font Info:    Overwriting math alphabet `\mathit' in version `bold'
(Font)              OT1/cmr/bx/it --> T1/Merriwthr-OsF/m/it on input
line 5
8.
LaTeX Font Info:    Redeclaring math alphabet \mathtt on input line 58.
LaTeX Font Info:    Overwriting math alphabet `\mathtt' in version
`normal'
(Font)              OT1/cmtt/m/n --> T1/lmtt/m/up on input line 58.
LaTeX Font Info:    Overwriting math alphabet `\mathtt' in version `bold'
(Font)              OT1/cmtt/m/n --> T1/lmtt/m/up on input line 58.
LaTeX Font Info:    Overwriting math alphabet `\mathsf' in version `bold'
(Font)              T1/MerriwthrSans-OsF/m/up --> T1/MerriwthrSans-
OsF/b/up
 on input line 58.
LaTeX Font Info:    Overwriting math alphabet `\mathit' in version `bold'
(Font)              T1/Merriwthr-OsF/m/it --> T1/Merriwthr-OsF/b/it
on inpu
t line 58.
\c@mv@tabular=\count323
\c@mv@boldtabular=\count324
(c:/TeXLive/2022/texmf-dist/tex/context/base/mkii/supp-pdf.mkii
[Loading MPS to PDF converter (version 2006.09.02).]
\scratchcounter=\count325
\scratchdimen=\dimen263
\scratchbox=\box75
\nofMPsegments=\count326
\nofMParguments=\count327
\everyMPshowfont=\toks39
\MPscratchCnt=\count328
\MPscratchDim=\dimen264
\MPnumerator=\count329
\makeMPintoPDFobject=\count330
\everyMPtoPDFconversion=\toks40
) (c:/TeXLive/2022/texmf-dist/tex/latex/epstopdf-pkg/epstopdf-base.sty
Package: epstopdf-base 2020-01-24 v2.11 Base part for package epstopdf
```

```
Package epstopdf-base Info: Redefining graphics rule for `.eps' on input line 4
85.
(c:/TeXLive/2022/texmf-dist/tex/latex/latexconfig/epstopdf-sys.cfg
File: epstopdf-sys.cfg 2010/07/13 v1.3 Configuration of (r)epstopdf for TeX Liv
e
))
*geometry* driver: auto-detecting
*geometry* detected driver: pdftex
*geometry* verbose mode - [ preamble ] result:
* driver: pdftex
* paper: a4paper
* layout: <same size as paper>
* layoutoffset:(h,v)=(0.0pt,0.0pt)
* modes: includefoot twoside
* h-part:(L,W,R)=(54.64pt, 488.22787pt, 54.64pt)
* v-part:(T,H,B)=(66.0pt, 745.04684pt, 34.0pt)
* \paperwidth=597.50787pt
* \paperheight=845.04684pt
* \textwidth=488.22787pt
* \textheight=715.04684pt
* \oddsidemargin=-17.62999pt
* \evensidemargin=-17.62999pt
* \topmargin=-47.76999pt
* \headheight=17.5pt
* \headsep=24.0pt
* \topskip=10.0pt
* \footskip=30.0pt
* \marginparwidth=48.0pt
* \marginparsep=10.0pt
* \columnsep=18.0pt
* \skip\footins=22.0pt plus 2.0pt
* \hoffset=0.0pt
* \voffset=0.0pt
* \mag=1000
* \@twocolumntrue
* \@twosidetrue
* \@mparswitchtrue
* \@reversemarginfalse
* (1in=72.27pt=25.4mm, 1cm=28.453pt)

Package hyperref Info: Link coloring ON on input line 58.
(./main.out) (./main.out)
\@outlinefile=\write3
\openout3 = `main.out'.

\@gscitedetails=\box76
\@gscitedetailsheight=\skip163
\@gsheadbox=\box77
\@gsheadboxheight=\skip164
LaTeX Font Info:    Font shape `T1/Merriwthr-OsF/b/n' will be
(Font)              scaled to size 6.5pt on input line 58.
```

```
LaTeX Font Info:    Calculating math sizes for size <7.5> on input line
58.

LaTeX Font Warning: Font shape `T1/Merriwthr-OsF/m/up' undefined
(Font)              using `T1/Merriwthr-OsF/m/n' instead on input line
58.

LaTeX Font Info:    Font shape `T1/Merriwthr-OsF/m/up' will be
(Font)              scaled to size 6.24973pt on input line 58.
LaTeX Font Info:    Font shape `T1/Merriwthr-OsF/m/up' will be
(Font)              scaled to size 5.24997pt on input line 58.
LaTeX Font Info:    Trying to load font information for U+eur on input
line 58.

(c:/TeXLive/2022/texmf-dist/tex/latex/amsfonts/ueur.fd
File: ueur.fd 2013/01/14 v3.01 Euler Roman
) (c:/TeXLive/2022/texmf-dist/tex/latex/microtype/mt-eur.cfg
File: mt-eur.cfg 2006/07/31 v1.1 microtype config. file: AMS Euler Roman
(RS)
)

LaTeX Font Warning: Font shape `OMS/cmsy/m/n' in size <7.5> not available
(Font)              size <7> substituted on input line 58.

LaTeX Font Info:    External font `cmex10' loaded for size
(Font)              <7.5> on input line 58.
LaTeX Font Info:    External font `cmex10' loaded for size
(Font)              <6.24973> on input line 58.
LaTeX Font Info:    External font `cmex10' loaded for size
(Font)              <5.24997> on input line 58.
LaTeX Font Info:    Trying to load font information for U+euf on input
line 58.

(c:/TeXLive/2022/texmf-dist/tex/latex/amsfonts/ueuf.fd
File: ueuf.fd 2013/01/14 v3.01 Euler Fraktur
) (c:/TeXLive/2022/texmf-dist/tex/latex/microtype/mt-euf.cfg
File: mt-euf.cfg 2006/07/03 v1.1 microtype config. file: AMS Euler
Fraktur (RS)

)
LaTeX Font Info:    Trying to load font information for U+eus on input
line 58.

(c:/TeXLive/2022/texmf-dist/tex/latex/amsfonts/ueus.fd
File: ueus.fd 2013/01/14 v3.01 Euler Script
) (c:/TeXLive/2022/texmf-dist/tex/latex/microtype/mt-eus.cfg
File: mt-eus.cfg 2006/07/28 v1.2 microtype config. file: AMS Euler Script
(RS)
)
LaTeX Font Info:    Trying to load font information for U+euex on input
line 58
.
(c:/TeXLive/2022/texmf-dist/tex/latex/amsfonts/ueuex.fd
File: ueuex.fd 2013/01/14 v3.01 Euler extra symbols
```

```
)

LaTeX Font Warning: Font shape `OML/cmm/m/it' in size <7.5> not available
(Font)              size <7> substituted on input line 58.

LaTeX Font Info:    Font shape `T1/Merriwthr-OsF/m/n' will be
(Font)              scaled to size 6.24973pt on input line 58.
LaTeX Font Info:    Font shape `T1/Merriwthr-OsF/m/n' will be
(Font)              scaled to size 5.24997pt on input line 58.
LaTeX Font Info:    Font shape `T1/Merriwthr-OsF/m/it' will be
(Font)              scaled to size 7.5pt on input line 58.
LaTeX Font Info:    Font shape `T1/Merriwthr-OsF/m/it' will be
(Font)              scaled to size 6.24973pt on input line 58.
LaTeX Font Info:    Font shape `T1/Merriwthr-OsF/m/it' will be
(Font)              scaled to size 5.24997pt on input line 58.
LaTeX Font Info:    Font shape `T1/Merriwthr-OsF/m/n' will be
(Font)              scaled to size 8.0pt on input line 58.
LaTeX Font Info:    Font shape `T1/Merriwthr-OsF/m/it' will be
(Font)              scaled to size 8.0pt on input line 58.
Package caption Info: Begin \AtBeginDocument code.
Package caption Info: End \AtBeginDocument code.

(c:/TeXLive/2022/texmf-dist/tex/latex/translations/translations-basic-
dictionar
y-english.trsl
File: translations-basic-dictionary-english.trsl (english translation
file `tra
nslations-basic-dictionary')
)
Package translations Info: loading dictionary `translations-basic-
dictionary' f
or `english'. on input line 58.
TextBlockOrigin set to 4pc+6.64pt x 4pc+6pt

Overfull \hbox (9.64pt too wide) in paragraph at lines 72--72
[][]
 []

LaTeX Font Info:    Font shape `T1/Merriwthr-OsF/b/n' will be
(Font)              scaled to size 18.0pt on input line 72.
LaTeX Font Info:    Font shape `T1/Merriwthr-OsF/m/n' will be
(Font)              scaled to size 9.0pt on input line 72.
LaTeX Font Info:    Font shape `T1/Merriwthr-OsF/m/up' will be
(Font)              scaled to size 9.0pt on input line 72.
LaTeX Font Info:    Font shape `T1/Merriwthr-OsF/m/up' will be
(Font)              scaled to size 7.0pt on input line 72.
LaTeX Font Info:    Font shape `T1/Merriwthr-OsF/m/up' will be
(Font)              scaled to size 5.0pt on input line 72.
LaTeX Font Info:    External font `cmex10' loaded for size
(Font)              <9> on input line 72.
LaTeX Font Info:    External font `cmex10' loaded for size
(Font)              <7> on input line 72.
LaTeX Font Info:    External font `cmex10' loaded for size
(Font)              <5> on input line 72.
```

```
LaTeX Font Info:    Font shape `T1/Merriwthr-OsF/m/n' will be
(Font)              scaled to size 7.0pt on input line 72.
LaTeX Font Info:    Font shape `T1/Merriwthr-OsF/m/n' will be
(Font)              scaled to size 5.0pt on input line 72.
LaTeX Font Info:    Font shape `T1/Merriwthr-OsF/m/it' will be
(Font)              scaled to size 9.0pt on input line 72.
LaTeX Font Info:    Font shape `T1/Merriwthr-OsF/m/it' will be
(Font)              scaled to size 7.0pt on input line 72.
LaTeX Font Info:    Font shape `T1/Merriwthr-OsF/m/it' will be
(Font)              scaled to size 5.0pt on input line 72.
LaTeX Font Info:    Trying to load font information for TS1+Merriwthr-OsF
on in
put line 72.
(c:/TeXLive/2022/texmf-dist/tex/latex/merriweather/TS1Merriwthr-OsF.fd
File: TS1Merriwthr-OsF.fd 2020/08/30 (autoinst) Font definitions for
TS1/Merriw
thr-OsF.
)
LaTeX Font Info:    Font shape `TS1/Merriwthr-OsF/m/n' will be
(Font)              scaled to size 7.0pt on input line 72.
Package microtype Info: Loading generic protrusion settings for font
family
(microtype)             `Merriwthr-OsF' (encoding: TS1).
(microtype)             For optimal results, create family-specific
settings.
(microtype)             See the microtype manual for details.
LaTeX Font Info:    Font shape `TS1/Merriwthr-OsF/m/n' will be
(Font)              scaled to size 5.0pt on input line 72.
LaTeX Font Info:    Font shape `T1/Merriwthr-OsF/b/n' will be
(Font)              scaled to size 7.0pt on input line 72.
LaTeX Font Info:    Font shape `T1/Merriwthr-OsF/b/n' will be
(Font)              scaled to size 10.0pt on input line 72.
LaTeX Font Info:    Font shape `T1/Merriwthr-OsF/b/n' will be
(Font)              scaled to size 8.0pt on input line 72.
LaTeX Font Info:    Font shape `T1/Merriwthr-OsF/m/up' will be
(Font)              scaled to size 8.0pt on input line 72.
LaTeX Font Info:    Font shape `T1/Merriwthr-OsF/m/up' will be
(Font)              scaled to size 6.0pt on input line 72.
LaTeX Font Info:    External font `cmex10' loaded for size
(Font)              <8> on input line 72.
LaTeX Font Info:    External font `cmex10' loaded for size
(Font)              <6> on input line 72.
LaTeX Font Info:    Font shape `T1/Merriwthr-OsF/m/n' will be
(Font)              scaled to size 6.0pt on input line 72.
LaTeX Font Info:    Font shape `T1/Merriwthr-OsF/m/it' will be
(Font)              scaled to size 6.0pt on input line 72.
LaTeX Font Info:    Font shape `T1/Merriwthr-OsF/m/n' will be
(Font)              scaled to size 6.5pt on input line 72.
Package mdframed Info: mdframed works in twoside mode on input line 75.
LaTeX Font Info:    Font shape `T1/Merriwthr-OsF/b/n' will be
(Font)              scaled to size 8.2pt on input line 75.
LaTeX Font Info:    Font shape `TS1/Merriwthr-OsF/m/n' will be
(Font)              scaled to size 7.5pt on input line 77.
Package mdframed Info: mdframed inside float
```

```
 mdframed uses option nobreak mdframed on input line 80.
Package mdframed Info: mdframed inside a box
 mdframed uses option nobreak mdframed on input line 80.
LaTeX Font Info:    Font shape `T1/Merriwthr-OsF/b/n' will be
(Font)              scaled to size 8.5pt on input line 103.
LaTeX Font Info:    Font shape `T1/Merriwthr-OsF/b/n' will be
(Font)              scaled to size 7.5pt on input line 106.

Package natbib Warning: Citation `clevers2016' on page 1 undefined on
input lin
e 106.


Package natbib Warning: Citation `kim2020human' on page 1 undefined on
input li
ne 106.


Package natbib Warning: Citation `kim2020human' on page 1 undefined on
input li
ne 106.


Package natbib Warning: Citation `drost2018organoids' on page 1 undefined
on in
put line 106.


Package natbib Warning: Citation `tuveson2019' on page 1 undefined on
input lin
e 106.


Package natbib Warning: Citation `lesavage2022next' on page 1 undefined
on inpu
t line 106.


Underfull \vbox (badness 8000) has occurred while \output is active []


Package natbib Warning: Citation `dayton2023druggable' on page 1
undefined on i
nput line 108.


Package natbib Warning: Citation `rindi2018' on page 1 undefined on input
line
108.


Package natbib Warning: Citation `travis2022lung' on page 1 undefined on
input
line 108.
```

```
Package natbib Warning: Citation `klimstra2019classification' on page 1
undefin
ed on input line 108.


Package natbib Warning: Citation `rudin2019' on page 1 undefined on input
line
108.


Package natbib Warning: Citation `derks2018' on page 1 undefined on input
line
108.


Package natbib Warning: Citation `fernandez2019molecular' on page 1
undefined o
n input line 108.


Package natbib Warning: Citation `dayton2023druggable' on page 1
undefined on i
nput line 110.

LaTeX Font Info:    Font shape `T1/Merriwthr-OsF/m/up' will be
(Font)              scaled to size 7.5pt on input line 110.

Underfull \vbox (badness 5105) has occurred while \output is active []

LaTeX Font Info:    Font shape `T1/Merriwthr-OsF/m/n' will be
(Font)              scaled to size 7.8pt on input line 111.
LaTeX Font Info:    Font shape `T1/Merriwthr-OsF/b/n' will be
(Font)              scaled to size 7.8pt on input line 111.
[1{c:/TeXLive/2022/texmf-var/fonts/map/pdftex/updmap/pdftex.map}


]
LaTeX Font Info:    Font shape `T1/Merriwthr-OsF/b/sl' in size <7.5> not
availa
ble
(Font)              Font shape `T1/Merriwthr-OsF/b/it' tried instead on
input l
ine 116.
LaTeX Font Info:    Font shape `T1/Merriwthr-OsF/b/it' will be
(Font)              scaled to size 7.5pt on input line 116.

Package natbib Warning: Citation `dayton2023druggable' on page 2
undefined on i
nput line 117.
```

```
Package natbib Warning: Citation `zhao2022organoids' on page 2 undefined
on inp
ut line 117.


Package natbib Warning: Citation `dayton2023druggable' on page 2
undefined on i
nput line 117.


Package natbib Warning: Citation `dayton2023druggable' on page 2
undefined on i
nput line 117.


Underfull \hbox (badness 10000) in paragraph at lines 151--151
[]|\T1/Merriwthr-OsF/m/n/6.8438 (+20) small in-tes-tine
 []


Underfull \hbox (badness 10000) in paragraph at lines 151--151
[]|\T1/Merriwthr-OsF/m/n/6.8438 (+20) small in-tes-tine
 []


Underfull \hbox (badness 10000) in paragraph at lines 151--151
[]|\T1/Merriwthr-OsF/m/n/6.8438 (+20) small in-tes-tine
 []


Underfull \hbox (badness 10000) in paragraph at lines 151--151
[]|\T1/Merriwthr-OsF/m/n/6.8438 (+20) small in-tes-tine
 []


Underfull \hbox (badness 10000) in paragraph at lines 151--151
[]|\T1/Merriwthr-OsF/m/n/6.8438 (+20) small in-tes-tine
 []


Package natbib Warning: Citation `iarcbioinfo' on page 2 undefined on
input lin
e 170.


Package natbib Warning: Citation `alcala2019integrative' on page 2
undefined on
 input line 170.


Package natbib Warning: Citation `gabriel2020' on page 2 undefined on
input lin
e 170.
```

```
Package natbib Warning: Citation `ditommaso2017nextflow' on page 2
undefined on
 input line 170.


Package natbib Warning: Citation `dockerhub' on page 2 undefined on input
line
170.


Package natbib Warning: Citation `shub' on page 2 undefined on input line
170.


Package natbib Warning: Citation `alignment-nf' on page 2 undefined on
input li
ne 172.


Package natbib Warning: Citation `li2010fast' on page 2 undefined on
input line
 172.


Package natbib Warning: Citation `vasimuddin2019efficient' on page 2
undefined
on input line 172.


Package natbib Warning: Citation `faust2014samblaster' on page 2
undefined on i
nput line 172.


Package natbib Warning: Citation `tarasov2015sambamba' on page 2
undefined on i
nput line 172.


Package natbib Warning: Citation `RNAseq-nf' on page 2 undefined on input
line
174.


Package natbib Warning: Citation `krueger2012trim' on page 2 undefined on
input
 line 174.


Package natbib Warning: Citation `martin2011cutadapt' on page 2 undefined
on in
put line 174.
```

Package natbib Warning: Citation `dobin2013star' on page 2 undefined on input l
ine 174.

Package natbib Warning: Citation `abra-nf' on page 2 undefined on input line 17
6.

Package natbib Warning: Citation `mose2014abra' on page 2 undefined on input li
ne 176.

Package natbib Warning: Citation `BQSR-nf' on page 2 undefined on input line 17
6.

Package natbib Warning: Citation `van2013fastq' on page 2 undefined on input li
ne 176.

Package natbib Warning: Citation `benjamin2019calling' on page 2 undefined on i
nput line 179.

Package natbib Warning: Citation `van2020genomics' on page 2 undefined on input
 line 179.

Package natbib Warning: Citation `mutect-nf' on page 2 undefined on input line
179.

Package natbib Warning: Citation `dayton2023druggable' on page 2 undefined on i
nput line 179.

Package natbib Warning: Citation `danecek2021twelve' on page 2 undefined on inp
ut line 179.

Package natbib Warning: Citation `vcf_normalization-nf' on page 2 undefined on
input line 179.

Package natbib Warning: Citation `table_annovar-nf' on page 2 undefined on inpu
t line 179.

Package natbib Warning: Citation `kim2018strelka2' on page 2 undefined on input
 line 179.

Package natbib Warning: Citation `strelka-nf' on page 2 undefined on input line
 179.

Package natbib Warning: Citation `benjamin2019calling' on page 2 undefined on i
nput line 183.

Package natbib Warning: Citation `van2020genomics' on page 2 undefined on input
 line 183.

Package natbib Warning: Citation `mutect-nf' on page 2 undefined on input line
183.

Package natbib Warning: Citation `danecek2021twelve' on page 2 undefined on inp
ut line 183.

Package natbib Warning: Citation `vcf_normalization-nf' on page 2 undefined on
input line 183.

Package natbib Warning: Citation `table_annovar-nf' on page 2 undefined on inpu
t line 183.

Package natbib Warning: Citation `andrews2012' on page 2 undefined on input lin
e 189.

Package natbib Warning: Citation `ewels2016' on page 2 undefined on input line

```
189.


Underfull \hbox (badness 2221) in paragraph at lines 189--190
\T1/Merriwthr-OsF/m/up/7.5 (+20) Software FastQC (v0.11.9 [\T1/Merriwthr-
OsF/b/
n/7.5 (+20) ? \T1/Merriwthr-OsF/m/up/7.5 (+20) ]; RRID:SCR_014583) was
used
 []

LaTeX Font Info:    Font shape `T1/Merriwthr-OsF/m/it' will be
(Font)              scaled to size 7.8pt on input line 190.
[2]
Underfull \vbox (badness 10000) has occurred while \output is active []

[3]
<Fig1.pdf, id=155, 664.73944pt x 683.96747pt>
File: Fig1.pdf Graphic file (type pdf)
<use Fig1.pdf>
Package pdftex.def Info: Fig1.pdf  used on input line 195.
(pdftex.def)             Requested size: 235.11394pt x 241.9074pt.
LaTeX Font Info:    Font shape `T1/Merriwthr-OsF/b/n' will be
(Font)              scaled to size 6.0pt on input line 196.

Underfull \vbox (badness 10000) has occurred while \output is active []

<Fig2.pdf, id=167, 647.54097pt x 1337.53302pt>
File: Fig2.pdf Graphic file (type pdf)
<use Fig2.pdf>
Package pdftex.def Info: Fig2.pdf  used on input line 203.
(pdftex.def)             Requested size: 235.11394pt x 485.63416pt.

Package natbib Warning: Citation `okonechnikov2015qualimap' on page 4
undefined
 on input line 210.


Underfull \vbox (badness 10000) has occurred while \output is active []

[4 <./Fig1.pdf> <./Fig2.pdf

pdfTeX warning: pdflatex.exe (file ./Fig2.pdf): PDF inclusion: multiple
pdfs wi
th page group included in a single page
>]
Overfull \hbox (2.90384pt too wide) in paragraph at lines 265--265
[]|\T1/Merriwthr-OsF/m/n/7 (-20) Coverage|
 []


Overfull \hbox (3.98692pt too wide) in paragraph at lines 265--265
[]|\T1/Merriwthr-OsF/m/n/7 (-20) PANEC1N|
 []
```

```
Overfull \hbox (3.2594pt too wide) in paragraph at lines 265--265
[]|\T1/Merriwthr-OsF/m/n/7 (-20) PANEC1T|
 []


Overfull \hbox (11.79015pt too wide) in paragraph at lines 265--265
[]|\T1/Merriwthr-OsF/m/n/7 (-20) PANEC1Tp4|
 []


Overfull \hbox (14.80511pt too wide) in paragraph at lines 265--265
[]|\T1/Merriwthr-OsF/m/n/7 (-20) PANEC1Tp14|
 []


Overfull \hbox (8.48412pt too wide) in paragraph at lines 265--265
[]|\T1/Merriwthr-OsF/m/n/7 (-20) LNET2Np7|
 []


Overfull \hbox (11.77692pt too wide) in paragraph at lines 265--265
[]|\T1/Merriwthr-OsF/m/n/7 (-20) LNET2Np12|
 []


Overfull \hbox (11.34341pt too wide) in paragraph at lines 265--265
[]|\T1/Merriwthr-OsF/m/n/7 (-20) LNET2Tp12|
 []


Overfull \hbox (4.50949pt too wide) in paragraph at lines 265--265
[]|\T1/Merriwthr-OsF/m/n/7 (-20) LCNEC3N|
 []


Overfull \hbox (15.54509pt too wide) in paragraph at lines 265--265
[]|\T1/Merriwthr-OsF/m/n/7 (-20) LCNEC3Np12|
 []


Overfull \hbox (3.78197pt too wide) in paragraph at lines 265--265
[]|\T1/Merriwthr-OsF/m/n/7 (-20) LCNEC3T|
 []


Overfull \hbox (14.83376pt too wide) in paragraph at lines 265--265
[]|\T1/Merriwthr-OsF/m/n/7 (-20) LCNEC3Tp17|
 []


Overfull \hbox (13.5111pt too wide) in paragraph at lines 265--265
[]|\T1/Merriwthr-OsF/m/n/7 (-20) LCNEC4Np6|
 []
```

```
Overfull \hbox (4.37192pt too wide) in paragraph at lines 265--265
[]|\T1/Merriwthr-OsF/m/n/7 (-20) LCNEC4T|
 []


Overfull \hbox (12.40874pt too wide) in paragraph at lines 265--265
[]|\T1/Merriwthr-OsF/m/n/7 (-20) LCNEC4Tp7|
 []


Overfull \hbox (16.83344pt too wide) in paragraph at lines 265--265
[]|\T1/Merriwthr-OsF/m/n/7 (-20) LCNEC4Tp24|
 []


Overfull \hbox (0.58696pt too wide) in paragraph at lines 265--265
[]|\T1/Merriwthr-OsF/m/n/7 (-20) LNET5N|
 []


Overfull \hbox (8.39018pt too wide) in paragraph at lines 265--265
[]|\T1/Merriwthr-OsF/m/n/7 (-20) LNET5Tp4|
 []


Overfull \hbox (1.13234pt too wide) in paragraph at lines 265--265
[]|\T1/Merriwthr-OsF/m/n/7 (-20) LNET6N|
 []


Overfull \hbox (7.80367pt too wide) in paragraph at lines 265--265
[]|\T1/Merriwthr-OsF/m/n/7 (-20) LNET6Tp1|
 []


Overfull \hbox (2.83017pt too wide) in paragraph at lines 265--265
[]|\T1/Merriwthr-OsF/m/n/7 (-20) SINET7N|
 []


Overfull \hbox (4.31537pt too wide) in paragraph at lines 265--265
[]|\T1/Merriwthr-OsF/m/n/7 (-20) SINET7M|
 []


Overfull \hbox (12.392pt too wide) in paragraph at lines 265--265
[]|\T1/Merriwthr-OsF/m/n/7 (-20) SINET7Mp2|
 []


Overfull \hbox (3.32751pt too wide) in paragraph at lines 265--265
[]|\T1/Merriwthr-OsF/m/n/7 (-20) SINET8N|
```

```
 []


Overfull \hbox (4.81271pt too wide) in paragraph at lines 265--265
[]|\T1/Merriwthr-OsF/m/n/7 (-20) SINET8M|
 []


Overfull \hbox (12.88934pt too wide) in paragraph at lines 265--265
[]|\T1/Merriwthr-OsF/m/n/7 (-20) SINET8Mp2|
 []


Overfull \hbox (3.49902pt too wide) in paragraph at lines 265--265
[]|\T1/Merriwthr-OsF/m/n/7 (-20) SINET9N|
 []


Overfull \hbox (4.98422pt too wide) in paragraph at lines 265--265
[]|\T1/Merriwthr-OsF/m/n/7 (-20) SINET9M|
 []


Overfull \hbox (12.14505pt too wide) in paragraph at lines 265--265
[]|\T1/Merriwthr-OsF/m/n/7 (-20) SINET9Mp1|
 []


Overfull \hbox (4.14388pt too wide) in paragraph at lines 265--265
[]|\T1/Merriwthr-OsF/m/n/7 (-20) LNET10N|
 []


Overfull \hbox (3.41635pt too wide) in paragraph at lines 265--265
[]|\T1/Merriwthr-OsF/m/n/7 (-20) LNET10T|
 []


Overfull \hbox (11.9471pt too wide) in paragraph at lines 265--265
[]|\T1/Merriwthr-OsF/m/n/7 (-20) LNET10Tp4|
 []


Package natbib Warning: Citation `wang2012rseqc' on page 5 undefined on
input l
ine 273.


Underfull \vbox (badness 10000) has occurred while \output is active []

<Fig3.pdf, id=9296, 664.8398pt x 549.073pt>
File: Fig3.pdf Graphic file (type pdf)
<use Fig3.pdf>
Package pdftex.def Info: Fig3.pdf  used on input line 279.
```

```
(pdftex.def)             Requested size: 439.4021pt x 362.90036pt.

LaTeX Warning: `h' float specifier changed to `ht'.


Package natbib Warning: Citation `NGSCheckmate' on page 5 undefined on
input li
ne 287.


Package natbib Warning: Citation `NGSCheckmate' on page 5 undefined on
input li
ne 287.

<Fig4.pdf, id=9300, 491.38109pt x 505.99197pt>
File: Fig4.pdf Graphic file (type pdf)
<use Fig4.pdf>
Package pdftex.def Info: Fig4.pdf  used on input line 293.
(pdftex.def)             Requested size: 439.4021pt x 452.4701pt.

LaTeX Warning: `h' float specifier changed to `ht'.


Package natbib Warning: Citation `love2014moderated' on page 5 undefined
on inp
ut line 300.

<Fig5.pdf, id=9304, 414.36925pt x 718.6407pt>
File: Fig5.pdf Graphic file (type pdf)
<use Fig5.pdf>
Package pdftex.def Info: Fig5.pdf  used on input line 304.
(pdftex.def)             Requested size: 230.41064pt x 399.60693pt.

LaTeX Warning: `h' float specifier changed to `ht'.


Package natbib Warning: Citation `dayton2023druggable' on page 5
undefined on i
nput line 310.


Package natbib Warning: Citation `breiman2001random' on page 5 undefined
on inp
ut line 310.


Package natbib Warning: Citation `liaw2002classification' on page 5
undefined o
n input line 310.


Package natbib Warning: Citation `di2023molecular' on page 5 undefined on
input
 line 310.
```

```
Package natbib Warning: Citation `ioannidis2016revel' on page 5 undefined
on in
put line 310.

[5]

Package natbib Warning: Citation `MS_panNEN_organoids' on page 6
undefined on i
nput line 314.


Package natbib Warning: Citation `di2023molecular' on page 6 undefined on
input
 line 314.


Underfull \vbox (badness 7116) has occurred while \output is active []

<Fig6.pdf, id=9317, 844.77417pt x 573.28117pt>
File: Fig6.pdf Graphic file (type pdf)
<use Fig6.pdf>
Package pdftex.def Info: Fig6.pdf  used on input line 318.
(pdftex.def)             Requested size: 488.22787pt x 331.32239pt.

LaTeX Warning: `h' float specifier changed to `ht'.


Package natbib Warning: Citation `breiman2001random' on page 6 undefined
on inp
ut line 325.


Package natbib Warning: Citation `ishwaran2010high' on page 6 undefined
on inpu
t line 325.


Package natbib Warning: Citation `banerjee2012identifying' on page 6
undefined
on input line 325.

[6 <./Fig3.pdf>]

Package natbib Warning: Citation `dayton2023druggable' on page 7
undefined on i
nput line 333.


Package natbib Warning: Citation `MS_panNEN_organoids' on page 7
undefined on i
nput line 333.
```

Package natbib Warning: Citation `MS_panNEN_organoids' on page 7
undefined on i
nput line 333.

Package natbib Warning: Citation `MS_panNEN_organoids' on page 7
undefined on i
nput line 333.

Package natbib Warning: Citation `dayton2023druggable' on page 7
undefined on i
nput line 333.

Package natbib Warning: Citation `MS_panNEN_organoids' on page 7
undefined on i
nput line 333.

Package natbib Warning: Citation `banck2013genomic' on page 7 undefined
on inpu
t line 333.

Package natbib Warning: Citation `sei2015hereditary' on page 7 undefined
on inp
ut line 333.

Package natbib Warning: Citation `miyoshi2017genomic' on page 7 undefined
on in
put line 333.

Package natbib Warning: Citation `pelosi2018most' on page 7 undefined on
input
line 333.

Package natbib Warning: Citation `simbolo2018mutational' on page 7
undefined on
input line 333.

Package natbib Warning: Citation `walter2018genetic' on page 7 undefined
on inp
ut line 333.

Package natbib Warning: Citation `alcala2019integrative' on page 7
undefined on
input line 333.

```
Package natbib Warning: Citation `samsom2021driver' on page 7 undefined
on inpu
t line 333.


Package natbib Warning: Citation `fernandez2014frequent' on page 7
undefined on
 input line 333.


Package natbib Warning: Citation `alcala2019integrative' on page 7
undefined on
 input line 333.


Underfull \vbox (badness 10000) has occurred while \output is active []


Underfull \vbox (badness 10000) has occurred while \output is active []

[7 <./Fig4.pdf>]

Package natbib Warning: Citation `dayton2023druggable' on page 8
undefined on i
nput line 335.


Package natbib Warning: Citation `MS_panNEN_organoids' on page 8
undefined on i
nput line 335.


Package natbib Warning: Citation `dayton2023druggable' on page 8
undefined on i
nput line 337.


Package natbib Warning: Citation `alcala2019integrative' on page 8
undefined on
 input line 342.


Package natbib Warning: Citation `gabriel2020' on page 8 undefined on
input lin
e 342.


Package natbib Warning: Citation `mangiante2023' on page 8 undefined on
input l
ine 342.
```

```
Package natbib Warning: Citation `MS_panNEN_organoids' on page 8
undefined on i
nput line 342.


Package natbib Warning: Citation `MS_panNEN_organoids' on page 8
undefined on i
nput line 342.


Package natbib Warning: Citation `lee2018tumor' on page 8 undefined on
input li
ne 344.


Package natbib Warning: Citation `fernandez2014frequent' on page 8
undefined on
 input line 347.


Package natbib Warning: Citation `alcala2019integrative' on page 8
undefined on
 input line 347.


Package natbib Warning: Citation `laddha2019integrative' on page 8
undefined on
 input line 347.


Package natbib Warning: Citation `alvarez2018precision' on page 8
undefined on
input line 347.


Package natbib Warning: Citation `hofving2021microenvironment' on page 8
undefi
ned on input line 347.

LaTeX Font Info:    Trying to load font information for T1+lmtt on input
line 3
53.
(c:/TeXLive/2022/texmf-dist/tex/latex/lm/t1lmtt.fd
File: t1lmtt.fd 2015/05/01 v1.6.1 Font defs for Latin Modern
)
Package microtype Info: Loading generic protrusion settings for font
family
(microtype)             `lmtt' (encoding: T1).
(microtype)             For optimal results, create family-specific
settings.
(microtype)             See the microtype manual for details.
[8 <./Fig5.pdf>]
```

```
Package natbib Warning: Citation `MS_panNEN_organoids' on page 9
undefined on i
nput line 360.


Package natbib Warning: Citation `MS_panNEN_organoids' on page 9
undefined on i
nput line 360.


Package natbib Warning: Citation `egaclient' on page 9 undefined on input
line
365.


Package natbib Warning: Citation `egaclientvideo' on page 9 undefined on
input
line 365.


Package natbib Warning: Citation `MS_panNEN_organoids' on page 9
undefined on i
nput line 365.


Underfull \hbox (badness 3849) in paragraph at lines 365--366
\T1/Merriwthr-OsF/m/up/7.5 (+20) The data set sup-port-ing the re-sults
of this
 ar-ti-cle is avail-
 []


Underfull \hbox (badness 5374) in paragraph at lines 365--366
\T1/Merriwthr-OsF/m/up/7.5 (+20) able in the Eu-ro-pean Genome-Phenome
archive
repos-i-tory,
 []


Underfull \hbox (badness 1127) in paragraph at lines 365--366
\T1/Merriwthr-OsF/m/up/7.5 (+20) EGAD00001009989 with WGS CRAM files for
6 ex-p
er-i-ments,
 []


Package natbib Warning: Citation `gigaDB' on page 9 undefined on input
line 369
.


Underfull \hbox (badness 2435) in paragraph at lines 371--372
\T1/Merriwthr-OsF/m/up/7.5 (+20) from Hans Clevers
(h.clevers@hubrecht.eu) or T
```

```
alya Day-ton
 []

[9 <./Fig6.pdf>]

Package natbib Warning: Citation `JCClevers' on page 10 undefined on
input line
 391.

No file main.bbl.
[10

]

LaTeX Warning: File `SI/FigS_RF_othergenes_ROC.pdf' not found on input
line 429
.


! Package pdftex.def Error: File `SI/FigS_RF_othergenes_ROC.pdf' not
found: usi
ng draft setting.

See the pdftex.def package documentation for explanation.
Type  H <return>  for immediate help.
 ...

l.429 ...textwidth]{SI/FigS_RF_othergenes_ROC.pdf}

Try typing  <return>  to proceed.
If that doesn't work, type  X <return>  to quit.


LaTeX Warning: `h' float specifier changed to `ht'.


Package natbib Warning: There were undefined citations.

[11]
enddocument/afterlastpage: lastpage setting LastPage.
(./main.aux)

LaTeX Font Warning: Size substitutions with differences
(Font)              up to 0.5pt have occurred.


LaTeX Font Warning: Some font shapes were not available, defaults
substituted.

Package rerunfilecheck Info: File `main.out' has not changed.
(rerunfilecheck)             Checksum:
58A4A285BA412AF15B34240B4A56E12F;4031.
 )
Here is how much of TeX's memory you used:
```

```
 23822 strings out of 476024
 464375 string characters out of 5794017
 1933382 words of memory out of 5000000
 43189 multiletter control sequences out of 15000+600000
 1321656 words of font info for 504 fonts, out of 8000000 for 9000
 1141 hyphenation exceptions out of 8191
 123i,12n,131p,2010b,1070s stack positions out of
10000i,1000n,20000p,200000b,200000s
{c:/TeXLive/2022/texmf-dist/fonts/enc/dvips/lm/lm-
ec.enc}{c:/TeXLive/2022/tex
mf-
dist/fonts/enc/dvips/merriweather/merriwthr_posqbl.enc}{c:/TeXLive/2022/t
exm
f-
dist/fonts/enc/dvips/merriweather/merriwthr_owzwzj.enc}<c:/TeXLive/2022/t
exmf
-dist/fonts/type1/sorkin/merriweather/Merriwthr-
Bold.pfb><c:/TeXLive/2022/texmf
-dist/fonts/type1/sorkin/merriweather/Merriwthr-
BoldItalic.pfb><c:/TeXLive/2022
/texmf-dist/fonts/type1/sorkin/merriweather/Merriwthr-
Italic.pfb><c:/TeXLive/20
22/texmf-dist/fonts/type1/sorkin/merriweather/Merriwthr-
Regular.pfb><c:/TeXLive
/2022/texmf-
dist/fonts/type1/public/amsfonts/cm/cmsy6.pfb><c:/TeXLive/2022/texm
f-dist/fonts/type1/public/amsfonts/cm/cmsy7.pfb><c:/TeXLive/2022/texmf-
dist/fon
ts/type1/public/amsfonts/euler/eurm7.pfb><c:/TeXLive/2022/texmf-
dist/fonts/type
1/public/amsfonts/euler/eusm7.pfb><c:/TeXLive/2022/texmf-
dist/fonts/type1/publi
c/lm/lmtt8.pfb><c:/TeXLive/2022/texmf-dist/fonts/type1/public/tex-
gyre/qhvb.pfb
><c:/TeXLive/2022/texmf-dist/fonts/type1/public/tex-gyre/qhvr.pfb>
Output written on main.pdf (11 pages, 2845768 bytes).
PDF statistics:
 10057 PDF objects out of 10688 (max. 8388607)
 6726 compressed objects within 68 object streams
 59 named destinations out of 1000 (max. 500000)
 146159 words of extra memory for PDF output out of 154059 (max.
10000000)
```

Template figure

Click here

Figure S1

Click here to access/download;LaTeX - Figure (eps, ps, etc.);FigS_RF_othergenes_ROC.pdf

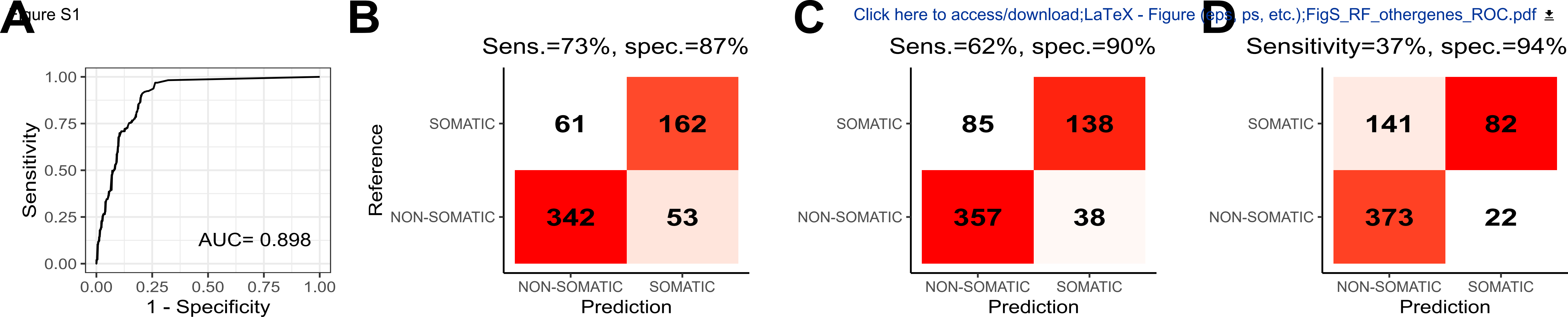

Manuscript Click here to access/download;Manuscript;GigaScience_Organoids_accept

Journal of X, 2023, 1–13

DOI: xx.xxxx/xxxx
Manuscript in Preparation
Data note

Multi-omic dataset of patient-derived tumor organoids of neuroendocrine neoplasms

Nicolas Alcala[1,*,†], Catherine Voegele[1], Lise Mangiante[1,2], Alexandra Sexton-Oates[1], Hans Clevers[3,4,5], Lynnette Fernandez-Cuesta[1], Talya L. Dayton[3,4,6,†] and Matthieu Foll[1,*,†]

[1]Rare Cancers Genomics Team (RCG), Genomic Epidemiology Branch (GEM), International Agency for Research on Cancer/World Health Organisation (IARC/WHO), Lyon, 69008, France
[2]Department of Medicine, Stanford University, Stanford, USA
[3]Hubrecht Institute, Royal Netherlands Academy of Arts and Sciences (KNAW) and UMC Utrecht, 3584 CT Utrecht, the Netherlands
[4]Oncode Institute, Hubrecht Institute, 3584 CT Utrecht, the Netherlands
[5]Current address: Roche Pharmaceutical Research and Early Development, Basel, Switzerland
[6]Current address: European Molecular Biology Laboratory (EMBL) Barcelona, Barcelona, Spain

[*]**Correspondence address.** 25 avenue Tony Garnier CS 90627 69366 Lyon Cedex 07, France. E-mail: alcalan@iarc.who.int (N.A.), follm@iarc.who.int (M.F.)
[†]These authors jointly supervised this work.

Abstract

Background: Organoids are three-dimensional experimental models that summarize the anatomical and functional structure of an organ. Although a promising experimental model for precision medicine, patient-derived tumor organoids (PDTOs) have currently been developed only for a fraction of tumor types.
Results: We have generated the first multi-omic dataset (whole-genome sequencing, WGS, and RNA-sequencing, RNA-seq) of PDTOs from the rare and understudied pulmonary neuroendocrine tumors ($n = 12$; 6 grade 1, 6 grade 2), and provide data from other rare neuroendocrine neoplasms: small intestine (ileal) neuroendocrine tumors ($n = 6$; 2 grade 1 and 4 grade 2) and large-cell neuroendocrine carcinoma ($n = 5$; 1 pancreatic and 4 pulmonary). This dataset includes a matched sample from the parental sample (primary tumor or metastasis) for a majority of samples (21/23) and longitudinal sampling of the PDTOs (1 to 2 time-points), for a total of $n = 47$ RNA-seq and $n = 33$ WGS. We here provide quality control for each technique, and provide the raw and processed data as well as all scripts for genomic analyses to ensure an optimal re-use of the data. In addition, we report gene expression data and somatic small variant calls and describe how they were generated, in particular how we used WGS somatic calls to train a random-forest classifier to detect variants in tumor-only RNA-seq. We also report all histopathological images used for medical diagnosis: hematoxylin and eosin-stained slides, brightfield images, and immunohistochemistry images of protein markers of clinical relevance.
Conclusions: This dataset will be critical to future studies relying on this PDTO biobank, such as drug screens for novel therapies and experiments investigating the mechanisms of carcinogenesis in these understudied diseases.

Keywords: organoid, cancer, neuroendocrine neoplasm, genomics, transcriptomics, quality control

Data Description

Context

Organoids are three-dimensional experimental models that summarize the anatomical and functional structure of an organ [1, 2]. Organoids are revolutionizing fundamental and medical research by allowing us to recapitulate human physiology better than animal models, and also allowing to recapitulate developmental biology contrary to traditional cell cultures [2]. Patient-derived tumor organoids (PDTOs) have been successfully derived for tumors, providing the experimental tools to model disease progression and the preclinical models for personalized treatment testing [3, 4, 5]. Although a promising experimental model, PDTOs have currently been developed only for a fraction of tumor types, focusing on the most frequent cancers and those easiest to culture, leaving rare cancers without appropriate experimental models.

We have recently described one of the very first patient-derived organoid biobanks for the rare and understudied neuroendocrine neoplasms [6]. Neuroendocrine neoplasms are rare tumors that can arise in multiple body sites, predominantly in the lung and gastrointestinal tract [7, 8, 9]. Neuroendocrine neoplasms are further classified into neuroendocrine tumors (NETs) and neuroendocrine carcinomas (NECs). NETs are themselves subdivided into grades (ranging from 1 to 2 or 3 depending on the organs), while NECs are subdivided into small cell and large cell (LCNEC). While small cell carcinomas are more common (e.g., 15% of lung tumors), benefited from more studies and have dedicated treatment options [10], the best treatment option for LCNEC is still unclear [11], and although most NETs progress slowly and have a good prognosis, a subgroup of tumors metastasize and relapse [12].

We report here the multi-omic dataset (whole-genome sequenc-

Compiled on: February 9, 2024.
Draft manuscript prepared by the author.

Key Points

- Tumor-derived organoids are revolutionary experimental resources to test biological hypotheses and treatment options
- We have generated the first multi-omic dataset for neuroendocrine tumor organoids of the lung, and for the rare neuroendocrine tumors of the pancreas, and small intestine (ileum)

ing, WGS, and RNA-sequencing, RNA-seq) of the neuroendocrine neoplasm PDTO biobank described in [6] (see Table 1). The dataset contains PDTOs of the lung (n = 12; 6 grade 1, 6 grade 2) and small intestine ileum (n = 6; 2 grade 1 and 4 grade 2), and LCNEC of the lung (n = 4) and pancreas (n = 1). This dataset includes longitudinal sampling of the organoids (2 to 3 time-points), and sequencing of the matched parental tumor for most samples (21/23, either primary tumors or metastases). Along with raw and processed data, we provide quality controls for each technique and scripts to run a complete molecular analysis. We also report hematoxylin and eosin-stained (H&E) slides for parental tumors and organoids, brightfield images of organoids, and immunohistochemistry images of neuroendocrine markers (Chromogranin A, Synaptofisin, CD56, and proliferation marker Ki67) and the EGFR protein. This unique dataset will provide a reference for future research on the understudied neuroendocrine neoplasms.

Methods

Sample collection

PDTO lines of the biobank described in [6] were established from surgical resections or biopsies, put in culture and expanded. PDTOs periodically underwent passaging, a process by which organoids are subcultured to allow future growth [13]; passage time varied from a week to several months depending on the growth rate ([6] Fig. 2). H&E stainings were performed and samples underwent an independent pathological review, and immunohistochemistry of common neuroendocrine markers (Chromogranin A, synaptophysin) were performed to confirm the tumoral neuroendrocrine nature of the parental tumors and PDTOs. See [6] for a detailed description of the protocol, and the GigaDB repository associated with this article for digital versions of H&E stainings and immunohistochemistry.

Extraction

For each tumor or PDTO, DNA and RNA were extracted from the same sample using the QIAGEN All Prep DNA/RNA Mini kit.

Sequencing

Whole-GenomeSequencing (WGS). Whole-genome sequencing was performed by the Utrecht Sequencing Facility. After DNA quality control, genomic DNA (0.5–1 μg) was used to prepare the whole-genome sequencing library, using the Illumina TruSeq DNA Nano Kit. Libraries were then sequenced on a Novaseq 6000 platform, as paired-end 150 bp reads, with a target average coverage of 30X for normal samples and 60X to 90X for tumor tissue and PDTOs.

RNA-Sequencing (RNA-seq). RNA sequencing was performed by the Utrecht Sequencing Facility. After RNA quality control, libraries were prepared using the Illumina TruSeq Stranded mRNA polyA Kit. Libraries were sequenced either on a Nextseq 2000 or an Illumina Novaseq 6000 (RRID:SCR_016387), as paired-end 150bp reads.

Data processing

All data processing was performed using the workflows developed by the rare cancers genomics team of the International Agency for Research on Cancer / World Health Organization [14], as detailed in [15] and [16]. The workflows are written in the popular domain-specific language nextflow [17]. All software dependencies are contained in conda environments and containerized with Docker and Singularity (containers available online [18, 19]).

WGS. Raw reads were mapped to reference genome GRCh38 using workflow *alignment-nf* v1.2 [20]. This workflow first maps reads (software bwa-mem2 v2.0 [21, 22]), then marks duplicates (software samblaster, v0.1.26 [23]), and finally sorts reads (software sambamba, v0.7.1 [24]).

RNA-seq. Raw reads were mapped to reference genome GRCh38 with annotation gencode v33 using the workflow *RNAseq-nf* v2.4 [25]. This workflow removes adapter sequences (wrapper Trim Galore v0.6.5 [26] for software cutadapt [27]), maps reads (software STAR v2.7.3a [28]), marks duplicated reads (software samblaster, v0.1.25), and finally sorts reads (software sambamba, v0.7.1).

Alignments were then post-processed using two workflows to improve their quality. Workflow *abra-nf* v3.0[29] performs local realignment using software ABRA2 (v2.22 [30]), and *BQSR-nf* v1.1[31] performs base quality score recalibration using gatk (v4.0.5.1 [32]).

Variant calling from WGS. Single nucleotide variants were called on all WGS samples using software Mutect2 from GATK4 (v4.2.0.0 [33, 34]) with workflow *mutect-nf* v2.2b[35], as described in [6]. Resulting variant calling format (VCF) files were normalized using bcftools v1.10.2 [36] (workflow vcf_normalization-nf v1.1 [37]) and annotated using ANNOVAR v2020Jun08 (workflow table_annovar-nf v1.1.1 [38]). Indels and multinucleotide variants were additionally filtered using the intersection of Mutect2 and strelka2 [39] calls (workflow strelka2-nf v1.2a[40]), in order to reduce false positives that are more frequent in indel calls due to the difficulty of detecting such variants with short reads sequencing.

Variant calling from RNA-seq. Variants were called on all RNA-seq samples using software Mutect2 from GATK4 (v4.2.0.0 [33, 34]) with workflow *mutect-nf* (branch RNAseq)[35], in RNA-seq and tumor-only modes. The RNA-seq mode incorporates a preprocessing step to fix CIGAR strings (removing NDN elements and ensuring that mapping quality 255 is not used as some mappers like STAR can do), and GATK4's SplitNCigarReads method that splits reads with Ns in their CIGAR string, in order to improve variant calling quality. Resulting variant calling format (VCF) files were normalized using bcftools v1.10.2 [36] (workflow vcf_normalization-nf v1.1[37]) and annotated using ANNOVAR v2020Jun08 (workflow table_annovar-nf v1.1.1 [38]). For samples which also had WGS data, RNA-seq-detected variants were classified as somatic or germline based on the WGS variant calls described above.

Quality control

For each 'omic technique, quality controls (QC) of the samples were performed at each step.

Raw reads

Software FastQC (v0.11.9 [41]; RRID:SCR_014583) was used to check raw reads quality, and software MultiQC (v1.9 [42]; RRID:SCR_005275) was used to aggregate the QC results across samples and generate interactive plots; all plots from Figs. 1 and

Table 1. Sample summary

ID	Primary site	Tumor type	WGS	RNA-seq	Normal sample (ID)	Tumor sample (ID)	Organoid passages (IDs)
LCNEC1	pancreas	LCNEC	yes	yes	blood (PANEC1N)	primary (PANEC1T)	4 (PANEC1Tp4), 14 (PANEC1Tp14)
LNET2	lung	NET (G1)	yes	no	normal-derived organoid passage 7 (LNET2Np12)	primary (LNET2T)	12 (LNET2Tp12), normal-derived organoid passage 12 (LNET2Np12)
LCNEC3	lung	LCNEC	yes	yes	tissue (LCNEC3N*)	primary (LCNEC3T)	17 (LCNEC3Tp17.2), 24 (LCNEC3Tp24)
LCNEC4	lung	LCNEC	yes	yes	normal-derived organoid passage 6 (LCNEC4Np6)	primary (LCNEC4T)	7 (LCNEC4Tp7), 24 (LCNEC4Tp24)
LNET5	lung	NET (G1)	yes	yes	blood (LNET5N)	primary (LNET5T)	4 (LNET5Tp4), 7 (LNET5Tp7), 2 (LNET5Tp2.2)†
LNET6	lung	NET (G1)	yes	yes	tissue (LNET6N)	primary (LNET6T)	1 (LNET6Tp1)
mSINET7	small intestine (ileum)	NET (G2)	yes	yes	blood (SINET7N)	mesenteric metastasis (SINET7M)	2 (SINET7Mp2)
mSINET8	small intestine (ileum)	NET (G2)	yes	yes	blood (SINET8N)	ovary metastasis (SINET8M)	2 (SINET8Mp2)
mSINET9	small intestine (ileum)	NET (G2)	yes	no	blood (SINET9N)	mesenteric metastasis (SINET9M)	1 (SINET9Tp1)
LNET10	lung	NET (G2)	yes	yes	blood (LNET10N)	primary (LNET10T)	4 (LNET10Tp4)
mLCNEC11	lung	LCNEC	no	yes	none	brain metastasis (LCNEC11M)	3 (LCNEC11Mp3)
mSINET12	small intestine (ileum)	NET (G2)	no	yes	none	mesenteric metastasis (SINET12M)	1 (SINET12Mp1 and SINET12Mp1.3)‡
LNET13	lung	NET (G1)	no	yes	none	primary (LNET13T)	1 (LNET13Tp1)
LNET14	lung	NET (G1)	no	yes	none	primary (LNET14T)	1 (LNET14Tp1)
mLNET15	lung	NET (G2)	no	yes	none	skin/soft tissue metastasis (LNET15M)	2 (LNET15Mp2)
LNET16	lung	NET (G2)	no	yes	none	primary (LNET16T)	2 (LNET16Tp2)
mLNET16	lung	NET (G2)	no	yes	none	metastasis to the ribcage (LNET16M)	1 (LNET16Mp1)
LNET18	lung	NET (G2)	no	yes	none	none	2 (LNET18Tp2, from primary)
LNET19	lung	NET (G1)	no	yes	none	primary (LNET19T)	2 (LNET19Tp2)
mLNET20	lung	NET (G2)	no	yes	none	paravertebral Th1 mestastasis (LNET20M)	2 (LNET20Mp2)
mSINET21	small intestine (ileum)	NET (G1)	no	yes	none	paravertebral Th1 mestastasis (SINET21M)	2 (SINET21Mp2)
mSINET22	lung	NET (G1)	no	yes	none	paravertebral Th1 mestastasis (SINET22M)	2 (SINET22Mp2)
mLCNEC23	unknown	LCNEC	no	yes	none	none	3 (LCNEC23Mp3, from paravertebral Th1 mestastasis)

for the normal samples, only WGS was performed
* one normal tissue for this experiment was excluded due to discordance with the tumor (see Fig. 4)
† Two lines were derived for LNET5, one sequenced at passages 4 and 7 (samples LNET5Tp4 and LNET5Tp7) and one at passage 2 (LNET5Tp2.2)
‡ Two lines were derived for SINET12, each sequenced at passage 1 (samples SINET12Mp1.1 and SINET12Mp1.3)

2 were generated by multiQC from the FastQC outputs. Original MultiQC reports are available in Supplementary Information (Files S1-S4) to allow a free exploration of the QC statistics.

WGS. Raw reads passed quality control filters in all samples. All samples displayed good sequence quality scores (mode above 30 Phred, indicating an error rate below 0.2%), both on average and across all positions in the read (Fig. 1A and B), with samples sequenced later (lower part of Table 1, from LNET5 to LNET10) displaying better scores (highest mode in Fig. 1A). GC content were slightly skewed toward lower values but proved consistent across samples (Fig. 1C), and adapter content (Fig. 1D, less than 5% of sequences with adapter sequence detected) and duplication levels (Fig. 1D, less than 20% of sequences present twice or more) were adequate and E). The number of reads were consistent between read pairs and consistent with target read depths (Fig. 1F): samples

with a target depth of 30X–normal, normal-derived organoids, the primary tumor from experiment LCNEC1, and tumor organoid passage 14 from experiment LCNEC3 (LCNEC3Tp14)–having a lower number of reads ($\sim 4 \times 100M$ reads= $400M$ reads) than the others samples ($\sim 4 \times 250M = 1000M$ reads), which had a target depth of 90X. Note that the metastasis organoid of experiment SINET9 (SINET9Mp1) has been sequenced in eight lanes, with 4 lanes with a low number of reads ($\sim 30M$) and 4 additional ones with a larger number ($\sim 140M$) so the total is comparable with that of the other samples.

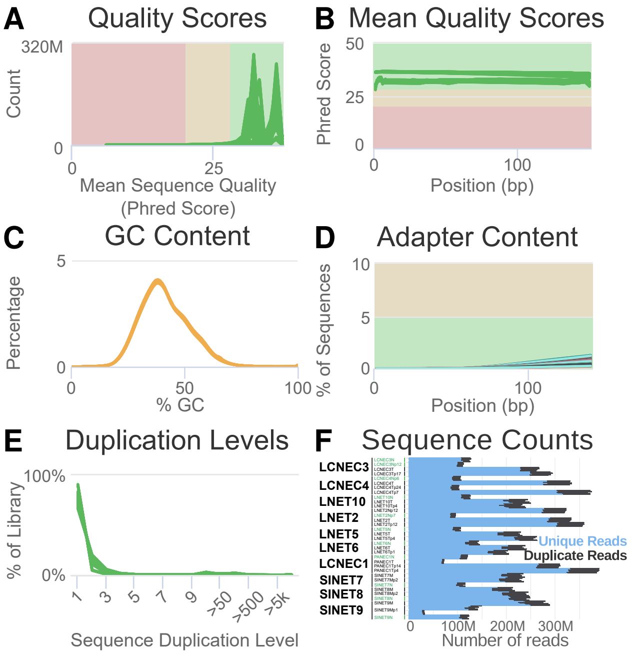

Figure 1. Quality control of the raw Whole-Genome Sequencing (WGS) data. (A) Distribution of the mean sequence quality of the reads in Phred score. (B) Mean sequence quality score as a function of the position in the read in base pairs (bp). (C) Distribution of the GC content in percent. (D) Percentage of reads containing a sequence corresponding to the Illumina adapter sequence as a function of the position in the read in bp. (E) Percentage of the library with a given level of duplication. (F) Number of unique and duplicated reads per file. In panels (A)–(E), each line corresponds to a fastq file, with each of the 34 samples from Table 1 subdivided into four sequencing lanes (except SINET9Mp1, subdivided into 8 lanes), and additionally subdivided into two read pair files, for a total of $4 \times 2 \times 33 + 8 \times 1 = 280$ files; in panel (F), each horizontal bar corresponds to a file. In (A)-(E), green lines correspond to files that passed the most stringent QC filters of software FastQC; orange lines correspond to files that passed a less stringent filter.

RNA-seq. Raw reads passed quality filters after reads trimming for adapter content and quality. All samples displayed good sequence quality scores on average both before and after read trimming (mode above 30 Phred; Fig. 2A and B), with samples sequenced later (lower part of Table 1, from LNET5 to LNET14) displaying better scores (highest mode in Fig. 1A). Six samples displayed lower scores at the end of the reads before trimming (Fig. 2C) but better scores after trimming (Fig. 2D). Indeed, most samples displayed high adapter content before trimming (Fig. 2E), and the trimming step successfully removed them (less than 0.1% in all samples; Supplementary Information File S1). The trimming step mostly removed less than 5 bp from the read, but occasionally could remove up to around 50 bp (Fig. 2F). GC content were consistent across samples (Fig. 2G-H), although the read trimming step resulted in an excess of reads with high GC content, presumably due to some reads being strongly shortened by the trimming step. Hopefully, in general the trimming step did not increase much the proportion of short reads (Fig. 2I-J). The number of reads were consistent between read pairs and across sequencing runs both before and after trimming (Fig. 2K-L), and total read numbers for each sample were consistent with the target number of $50M$ ($25M$ pairs): the smallest number, 60.8M corresponded to sample PANEC1Tp14.

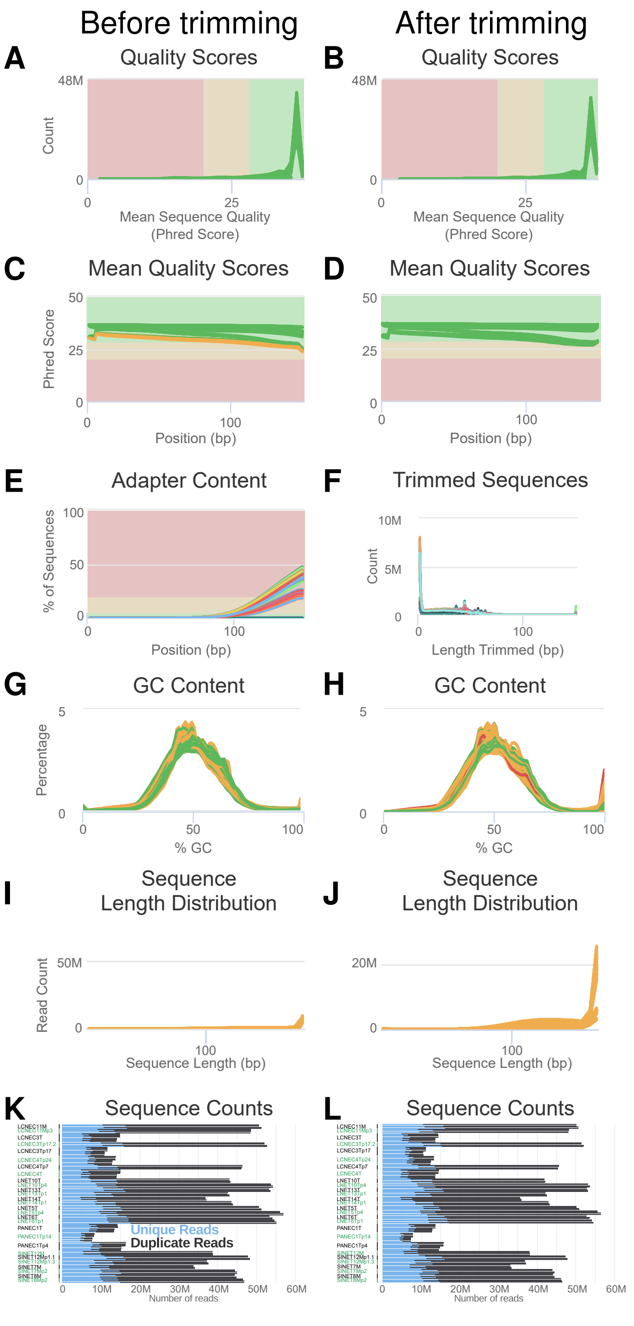

Figure 2. Quality control of the raw RNA-seq data. Panels (A), (C), (E), (G), (I), (K) correspond to controls before read trimming for quality and adapter content by wrapper Trim Galore for software cutadapt; panels (B), (D), (F), (H), (J), (L) correspond to controls after read trimming. Figure legends for panels (A)–(E) and (G)–(L) follow that of Fig. 1. (F) Distribution of the length of the reads trimmed by software cutadapt, for each file (colored lines). In panels (A)–(J), each line corresponds to a fastq file, with each of the 10 non-normal samples from Table 1 divided into two or four sequencing lanes, and further subdivided into two read pair files, for a total of $2 \times 2 \times 21 + 4 \times 2 \times 7 = 140$ files; in panels (K)–(L), each horizontal bar corresponds to a file.

Alignments

WGS. The software qualimap (v2.2.2b [43]; RRID:SCR_001209) was called by our workflow *alignment-nf* to generate QC statistics for

Table 2. Quality control of the WGS alignments

Sample Name	% GC	≥30X	≥50X	Coverage	% Aligned
PANEC1N	42%	84.6%	16.2%	41.0X	99.9%
PANEC1T	42%	93.8%	91.7%	104.0X	99.8%
PANEC1Tp4	42%	93.9%	93.3%	127.0X	99.9%
PANEC1Tp14	42%	93.6%	90.9%	89.0X	99.8%
LNET2Np7	41%	67.2%	1.9%	33.0X	99.9%
LNET2Np12	41%	93.4%	92.9%	104.0X	99.9%
LNET2T	41%	93.3%	92.9%	109.0X	99.9%
LNET2Tp12	42%	93.4%	93.1%	115.0X	99.8%
LCNEC3N	41%	82.5%	11.1%	39.0X	99.9%
LCNEC3Np12	42%	82.5%	11.7%	38.0X	99.9%
LCNEC3T	41%	93.9%	90.0%	89.0X	99.9%
LCNEC3Tp17	42%	93.5%	90.9%	90.0X	99.8%
LCNEC4Np6	42%	69.7%	2.7%	34.0X	99.9%
LCNEC4T	41%	93.0%	88.1%	102.0X	99.8%
LCNEC4Tp7	42%	91.7%	87.9%	102.0X	99.9%
LCNEC4Tp24	42%	51.9%	11.3%	30.0X	99.9%
LNET5N	41%	68.9%	2.5%	33.0X	99.9%
LNET5T	42%	91.9%	77.6%	68.0X	99.9%
LNET5Tp4	42%	93.3%	87.6%	75.0X	99.9%
LNET6N	42%	86.6%	22.8%	43.0X	99.9%
LNET6T	42%	93.0%	83.1%	72.0X	99.9%
LNET6Tp1	42%	90.2%	76.8%	61.0X	99.9%
SINET7N	42%	77.2%	4.9%	36.0X	99.9%
SINET7M	41%	92.8%	83.5%	73.0X	99.9%
SINET7Mp2	42%	92.8%	85.7%	69.0X	99.9%
SINET8N	42%	93.1%	91.7%	75.0X	99.9%
SINET8M	41%	92.6%	81.2%	64.0X	99.9%
SINET8Mp2	42%	93.0%	85.5%	70.0X	99.9%
SINET9N	42%	84.4%	7.2%	38.0X	99.9%
SINET9M	41%	93.0%	90.0%	81.0X	99.9%
SINET9Mp1	42%	90.2%	49.1%	49.0X	99.9%
LNET10N	42%	86.4%	9.5%	39.0X	99.9%
LNET10T	42%	93.0%	90.1%	71.0X	99.9%
LNET10Tp4	42%	93.0%	87.1%	66.0X	99.9%

the WGS alignments in parallel to the data processing (Table 2). All normal and normal tissue-derived organoids displayed a mean coverage ≥30X, and all tumor and tumor-derived organoids except passage 24 from the organoid of experiment LCNEC4 (sample LCNEC4Tp24) and passage 1 of the organoid of experiment SINET9 (sample SINET9Mp1) had a coverage ≥ 60X; all samples displayed at least 65% of the genome with a coverage larger than or equal to 30X except LCNEC4Tp24 and (57.4%). Percentages of aligned reads exceeded 99.8% for all samples. Interestingly, some tumor and tumor-derived organoid samples displayed bimodal coverage distributions compatible with variations in copy number state (Supplementary Information File S3).

RNA-seq. Software RSeQC (v3.0.1 [44]; RRID:SCR_005275) was called to check alignment quality in parallel to the data processing by workflow *RNAseq-nf*. For all samples, the number of known junctions (i.e., junctions annotated in the gencode v33 annotation file) was stable when resampling subsets of 75% to a 100% of the reads (all lines plateau in Fig. 3A), indicating a good saturation and suggesting that the sequencing depth was sufficient to detect known junctions. In contrast, the number of novel junctions (i.e., junctions not in the annotation file) was increasing slowly as a function of the percentage of reads resampled, but did not completely saturate (no complete plateau in Fig. 3B). This indicates that we probably detected the most abundant novel junctions but that some low abundance novel junctions were probably not detected.

Alignment scores were good, with more than 25M mapped read pairs (50M reads) for all samples, and from 4M to 7M unmapped reads, mainly due to reads being too short or having too many mismatches (Fig. 3C). The distribution of the alignments within annotated regions matched our expectations, with most reads (≥80%) either aligning to exons (≥50%), 3' UTR (~3%), and 5' UTR (~25%) (Fig. 3D).

Data Validation

Sample matching

We used software NGSCheckMate (cloned from the github repository [45] revision 10799087bdfe4b990add5b5e536f87c47bbdb688; RRID:SCR_022994) to check that samples from the same experiment indeed came from the same individual, in both WGS and RNA-seq simultaneously, using our workflow *NGSCheckMate-nf* v1.1[45]. The sample matching algorithm correctly identified all experiments except one (Fig. 4). The WGS normal-derived organoid sample from experiment LCNEC3 (LCNEC3Np12_WGS in Fig. 4) was found not to match other LCNEC3 samples, suggesting a possible sample swap and was thus excluded from further analyses. Also, the RNA-seq tumor sample for the late-passage organoid of experiment LCNEC3 (sample LCNEC3Tp17_RNA in Fig. 4) was found to better match experiment LNET2, and was thus excluded from the subsequent analyses. Finally, two samples were found to partially match LNET15 and LNET16, suggesting contamination and were also excluded (UNKN00 and UNKN01).

Sex validation

We validated the sex reported in the clinical data using the multi-omic data. For the WGS data, we used the proportion of reads aligned to the sex chromosomes to assess whether samples clustered by sex (Fig. 5A). We found that all samples clustered by sex except for the normal of experiment LCNEC3 (sample LCNEC3Np12) which clustered with females despite other samples from the experiment clearly clustering with males. This further supports the sample matching reports that suggest that this sample does not match the rest of the experiment. For the RNA-seq data, we compared the total expression level on the sex chromosomes, using the variance-stabilised read counts as a quantification of gene expression (vst function from R package DESeq2 v1.26.0 [46]) (Fig. 5B). We find that samples from the same sex cluster together for all experiments, suggesting concordance with the clinical data.

Small variant calls from RNA-seq

We classified small variants called from RNA-seq in 241 known neuroendocrine neoplasm driver genes (from Table S4 in [6]) as somatic or germline, using a random forest (RF) algorithm [47] (R package randomForest v4.7-1.1 [48]; Fig. 6), using a similar approach as we recently did to classify mutations in tumor-only WGS [49]. After filtering out non-exonic, synonymous, and nonsynonymous mutations with a REVEL score [50] below 0.5, and mutations not in the list of 241 drivers, we were left with 2430 variants. Among them, 1174 variants were in samples with WGS data available and their somatic status was thus known.

We used 10 features in the RF model. One feature was directly informative about the potential germline status and came from a public database: the frequency of the allele in human populations from the ExAc database excluding cancers from the TCGA (feature *ExAC_nontcga_ALL*). Four features were informative about the alignment and came from the sequencing data itself: the median distance from the end of the read (feature *MPOS*), the likelihood ratio score of variant existence (feature *TLOD*), the coverage at the position (feature *DP*), and the allelic fraction of the alternative al-

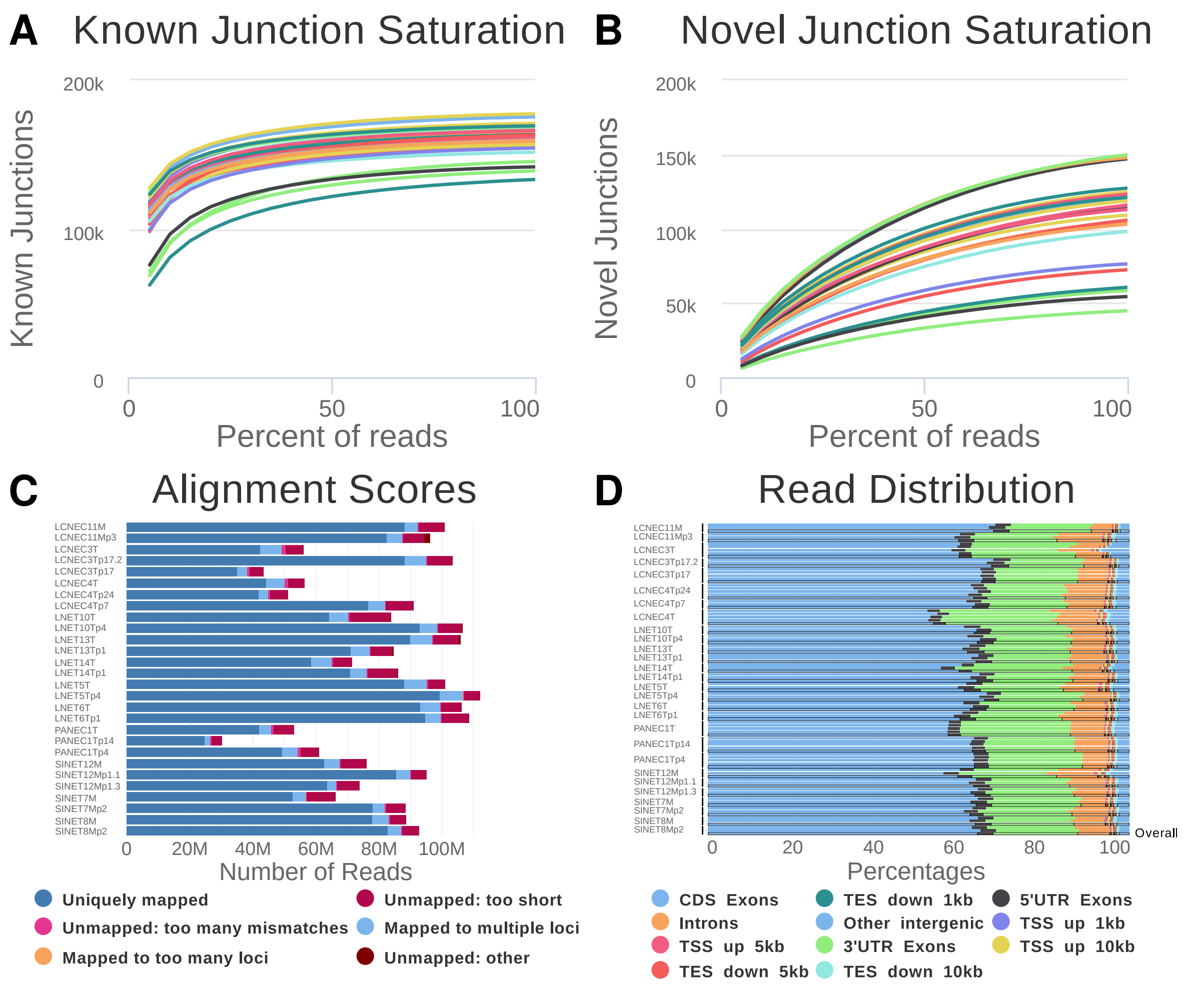

Figure 3. Quality control of the RNA-seq alignments. (A) Number of known junctions identified by software STAR in a subsample as a function of the percentage of reads in the subsample. (B) Number of novel junctions identified by STAR in a subsample as a function of the percentage of reads in the subsample. (C) Number of sequence tags with each alignment score. (D) Distribution of reads among annotated regions.

lele (RNA.AF). Finally, the other features were informative about the pathogenicity of the variant and came from public databases: the REVEL score of pathogenicity (feature *REVEL*), the presence in the COSMIC 92 database (feature *cosmic92_coding_nonnull*), the presence in the COSMIC 92 database in a lung tumor (feature *cosmic92_coding_lung*), and the InterVar annotation (feature *InterVar_automated*; with levels ".", "Uncertain_significance", "likely_pathogenic", and "Pathogenic"), and the exonic function of the variant (missense, nonsense, inframe or frameshift insertion, etc).

The RF algorithm was trained and tested on the 1174 variants with known status (1148 germline, 26 somatic) called in 22 samples from 8 experiments (Fig. 6A). Note that although the data is imbalanced, we chose to keep this imbalance in the training set to force the algorithm to take into account the fact that most variants are not somatic, and thus having a very good specificity is key to avoid large false discovery rates. We used leave-one-out cross-validation at the experiment level (8 folds), excluding all samples from one same experiment from the model fit at each iteration in order to avoid over-fitting due to the inclusion of variants from the same individual but different samples (e.g., LCNEC3T and LCNEC3Tp17) in the training and test sets. We used 5000 trees, and 3 features per split (the square root of the total number of features as recommended by default), and a minimal node size of 1. We estimated the performance of the model using the receiver operating characteristic (ROC) curve and its area under the curve (AUC, computed using the trapezoid rule), showing the sensitivity as a function of 1-specificity across different thresholds for the proportion of votes for the somatic class. We also computed the false discovery rate to get a sense of the proportion of variants classified as somatic that would actually be false positives. Once the RF model performance was assessed, we trained a RF model on the full 1174 variants and predicted the status of the remaining 1256 variants. See github repository associated with the manuscript for the complete R script [51]. Note that the same approach allowed to classify variants called from tumor-only WGS data as somatic or germline with high performance (accuracy greater than 92%; Di Genova et al. 49).

We find that we can classify variants as somatic or germline with a balanced accuracy of 86%, with both specificity greater than 98% and sensitivity greater than 73% (AUC=0.965). Interestingly, although somatic variants are just a fraction of the calls (2%), the high sensitivities and specificities of our RF algorithm allowed to classify variants with false discovery rates below 50% while still preserving sensitivities above 60% (see Fig. 6B, E-G). We also tested the predictive accuracy of the model fitted on this set of 1174 variants from known neuroendocrine neoplasm genes on the set of somatic variants from other recurrently mutated genes in our cohort (Fig. S1). We find that the predictive power of the RF model was similar (AUC=0.90, sensitivity up to 73% with a specificity above 87%).

We evaluated the importance of features for the classification both using the mean decrease in accuracy, which captures how much the model loses accuracy when the feature is excluded, and using the mean tree depth at which the feature was observed, with a low value meaning that the feature is used early in the decision trees and thus separates many variants[47, 52] (R package randomFores-

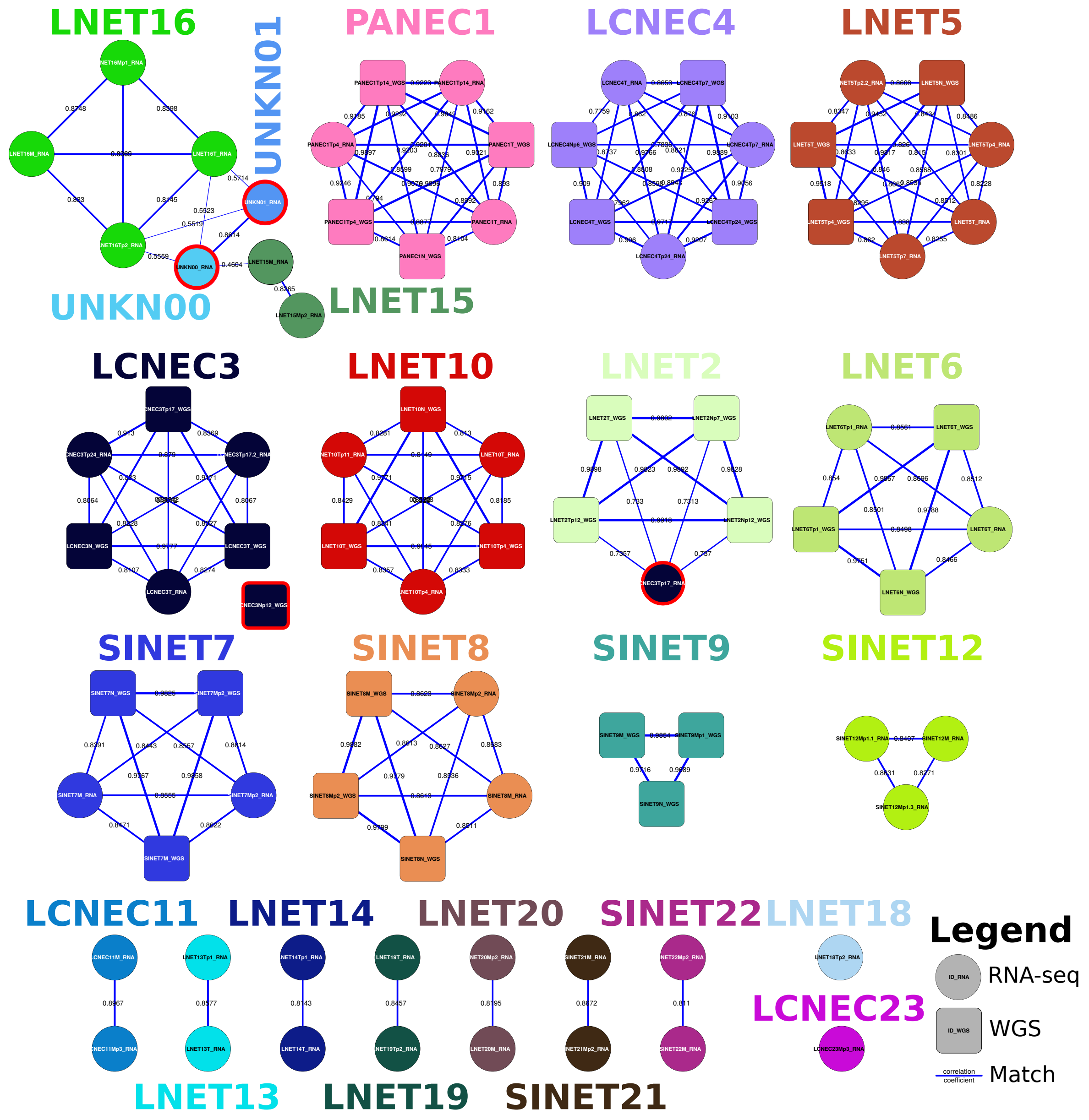

Figure 4. Network of matches between WGS and RNA-seq samples, computed with software NGSCheckmate. Numbers on the edges and edge thickness correspond to the Pearson correlation coefficient *r* between allelic fractions for the germline SNP panel; colors: experiments (see Table 1); squares: WGS, circles: RNA-seq, red contour: mismatches.

tExplainer v0.10.1). The most important features for the classification were the REVEL score, the TLOD, and the cosmic annotation, while the frequency in the ExAC database was the least important, presumably because all these variants were very rare (Fig. 6C). Indeed, the most representative tree from the RF, computed using the reprtree R package v0.6 using the d2 distance metric between tree predictions[53], relied on these three variables, with all alterations present in a lung tumor from the COSMIC 92 database automatically classified as somatic (root of the tree), and TLOD and REVEL score being the most common features used for splitting (Fig. 6D). Of note, using the most important feature alone (the REVEL score) led to a much lower accuracy, consistent with the importance of other features such as TLOD and pathogenic annotations (COSMIC, InterVar).

Comparing molecular profiles of PDTOs and parental tumors

We report here all the R scripts used in Dayton et al. [6] to validate that PDTOs faithfully represent their parental tumors (available on the github repository associated with the manuscript [51]). In particular, we provide the code that we used to compare the expression profiles of PDTOs and reference lung and SI NETs and LCNECs with that of PDTOs and their parental tumors (file Fig3B_S3BCE.md [51]). This analysis confirmed the neuroendocrine nature of the PDTOs by showing that they express neuroendocrine markers routinely used in the clinic (>1 TPM in at least one of 6 markers). We also provide the code (file Fig3CD_S3FGHI.md [51]) used in Dayton et al. [6] to demonstrate that pure PDTOs preserve the expression profiles of their parental tumor using dimensionality reduction techniques (UMAP). In addition, we provide the code (files Fig4BC_S4BC.md and Fig4D_S4D.md [51]) used to show that PDTOs preserve the genomic profile (small variants, copy number variants, and structural variants) of their parental tumor. To do so, we focused on mutations known to be drivers of neuroendocrine neoplasms [54, 55, 56, 57, 58, 59, 15, 60]. Both variants identified with WGS and variants identified with RNA-seq include driver mutations in key recurrently altered LCNEC driver genes such as *TP53* (mutated in 5/5 LCNEC) and *STK11* (mutated in 3/5 LCNEC). We also identified mutations or structural variants in known driver genes

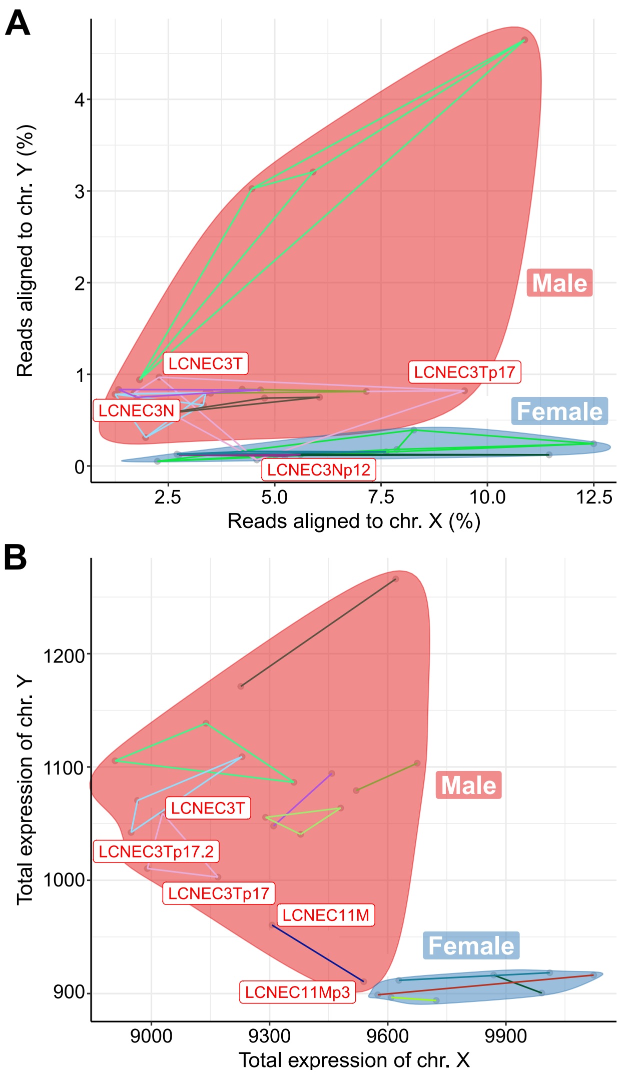

Figure 5. Validation of reported sex. (A) Percentage of reads aligned to chromosome X and Y in the whole-genome sequencing data. (B) Total gene expression in X and Y chromosome, in units of variance-stabilized read counts, computed from RNA-seq data. In all panels, samples from each sex are encircled (red:male, blue:female), excluding LCNEC3Np12, which we report as not matching the other samples from the LCNEC3 experiment.

in all but one neuroendocrine tumors (17/18), but as previously reported, they involve multiple genes instead of recurrently mutated genes [61, 15]. This confirms that PDTOs recapitulate the genomic profile of neuroendocrine neoplasms.

We also report the R scripts used in Dayton et al. [6] to analyze the temporal evolution of the PDTOs (file Fig5_S5.md [51]). These analyses showed that PDTOs preserve the genetic diversity and clonal architecture of their parents across long periods of time (6 months to more than a year). In particular, the analysis of two samples with multiple time points (LCNEC1 and LCNEC4) highlighted that the genetic makeup of the parental tumor is preserved across PDTO passages.

Of note, one sample, LCNEC23 was a paravertebral metastasis of an LCNEC of unknown primary. As mentioned in Dayton et al. [6] (Figure 3), the transcriptome of this sample did cluster with other LCNEC from the lung and pancreas; in addition, we detected from the RNA-seq two high-confidence somatic mutations characteristic of LCNEC: a nonsynonymous *TP53* and a nonsense *PIK3CA* mutation. These molecular results comfort the LCNEC nature of the PDTO, but the overlap between known lung and pancreas LCNEC profiles does not allow to infer the site of origin of the tumor.

Re-use potential

We describe here some of the very first multi-omic datasets for patient-derived tumor organoids of pancreatic, small intestine (ileum), and pulmonary neuroendocrine neoplasms, in particular including the first lung neuroendocrine tumor organoids. Because such low grade tumors are difficult to cultivate in vitro, there is currently a lack of adequate experimental systems for these tumors, and we expect the biobank associated with the data presented here to be the basis for future experimental studies–either fundamental or treatment oriented–on neuroendocrine neoplasms across body sites. The multi-omic dataset we provide here constitutes the molecular fingerprints of these experimental models, and will be key to investigate oncogenic processes responsible for tumor initiation and progression, and to link drug responses to molecular features to design future personalized treatments.

To facilitate future studies, we used the exact same data processing as in our previous studies of neuroendocrine neoplasms [15, 16] and other rare cancers [62], in particular using rigorous RNA-seq expression quantification with containerized software and operating systems (see methods section). To ease future studies, we make the expression matrix publicly available at file gene_expression_PDTOs_parents.tsv [51]. In addition, we provide all R scripts to analyze the data [51].

Note that the slow passage time of low-grade PDTOs makes them appropriate models to study the biology of neuroendocrine tumors, but challenges their use for drug testing. This is particularly true of small intestine NETs, which were only short term cultures that did not grow past four passage. Finally, as noted in most molecular studies of PDTOs [63], one of the main differences between PDTOs and their parental tumors is the absence of microenvironment. Future work would ideally focus on creating co-cultures of PDTOs and immune cells to remedy this shortcoming.

Conclusion

We have shown that our multi-omic dataset is of high quality and can be easily re-used. Given the rarity of neuroendocrine tumors from the lung, pancreas, and small intestine, past genomic studies each only reported data for a handful of samples, limiting the potential discoveries. For example, for lung NETs, 29 WGS and 39 RNAseq were reported in [61], 3 WGS and 20 RNA-seq in [15], and 30 RNA-seq in [64]; for small intestine NETs, for example, 81 RNA-seq with no WGS were reported in [65] and 7 RNA-seq in [66]). As a result, the primary tumors and metastasis sequencing data we report here (10 samples with WGS, 21 with RNA-seq) alone are very valuable, and should be combined with other datasets in future studies to provide enough power to discover informative molecular features for diagnosis, prognosis, and treatment. In addition, we report a unique multi-omic dataset generated from patient-derived tumor organoids, which will allow all researchers working on our biobank to test hypotheses regarding the molecular features associated with drug responses and thus advance research on personalized treatments for these understudied diseases.

Availability of source code and requirements

- Project name: NEN organoids project, lungNENomics
- Project home pages: `https://www.embl.org/groups/dayton/`, `http://rarecancersgenomics.com/lungnenomics/`
- Operating system(s): Platform independent
- Programming language: Nextflow, R
- Other requirements: R packages *caret*, *randomForest*
- License: GNU GPL

All nextflow command lines for data processing are available at

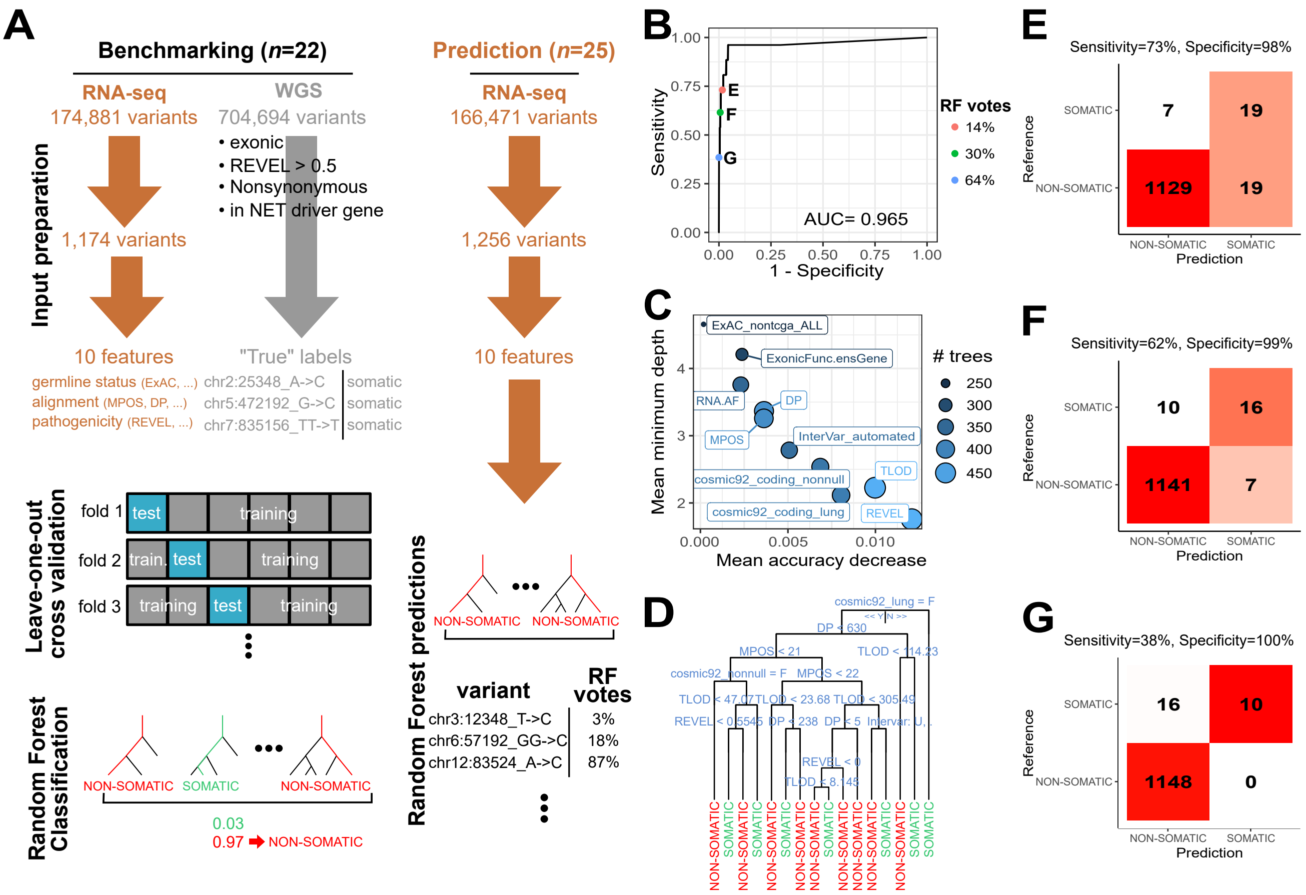

Figure 6. Random forest (RF) classification of variants as somatic or germline from RNA-seq data. (A) Schematic of the RF training, test, and prediction. B) Receiver operating characteristic (ROC) curve. C) Feature importance for classification accuracy. Mean accuracy decrease: mean difference in accuracy between trees with the feature and trees without the feature; high values indicate important features. Mean minimum depth: tree depth (1: root, value >> 1: leaves) of the first time the feature is used for classification, averaged across all trees; low values indicate features often used at the root and thus particularly important. D) Representative tree of the RF. At each split, the split condition is written above, the left branch corresponds to a Yes and the right branch to a No. Final decision (SOMATIC or NON-SOMATIC) is represented by the leaves. (E)-(G) Confusion matrix for different levels of sensitivity and specificity. Reference: somatic status assessed from whole-genome sequencing data. Prediction: somatic status predicted from RNA-seq data using the RF algorithm.

[51] in the readme. All R scripts for the analysis are available in the subfolder Rscripts [51].

Availability of supporting data and materials

The data set supporting the results of this article is available in the European Genome-Phenome archive repository, study EGAS00001005752. The study consists of seven datasets: EGAD00001009988, with WGS CRAM files for 2 experiments, EGAD00001009989 with WGS CRAM files for 6 experiments, EGAD00001009990, with WGS CRAM files for 2 experiments, EGAD00001009991 with RNA-seq fastq files from 4 experiments, EGAD00001009992, with RNAseq fastq files for 15 experiments, EGAD00001009993, with RNA-seq fastq files for 2 experiments, and EGAD00001009994, with gene expression in multiple formats (R data, tab-separated text files) and multiple units (raw counts, TPM, FPKM) for 21 samples. Because of the sensitivity of the data and the patient consent, to get access to the data, please contact the data access committee of the Division of Biomedical Genetics from UMC Utrecht at dacdbg@umcutrecht.nl. Once a data access agreement has been signed and access granted, data can be downloaded using the EGA python client (see detailed instructions [67], and video tutorial [68]). Expression matrices in raw counts format and small variants are also publicly available on the github repository under the data folder[51].

The multiQC report for WGS raw reads is available in Supplementary File S1; the multiQC report for RNA-seq raw reads is available in Supplementary File S2; the multiQC report for WGS alignments is available in Supplementary File S3; the multiQC report for RNA-seq alignments is available in Supplementary File S4.

Snapshots of our code and other data further supporting this work are openly available in the GigaScience repository, GigaDB [69].

Organoid lines mentioned in this manuscript can be requested from Hans Clevers (h.clevers@hubrecht.eu) or Talya Dayton (talya.dayton@embl.es). Distribution of organoids to third parties will have to be authorized by the relevant ethical committee and a complete material transfer agreement will be required to ensure compliance with the Dutch 'medical research involving human subjects' act. Use of organoids is subjected to patient consent; note that upon consent withdrawal, distributed organoid lines and any derived material will have to be promptly disposed of.

Abbreviations

bp: base pairs. LCNEC: large-cell neuroendocrine carcinoma. NEC: neuroendocrine carcinoma. NEN: neuroendocrine neoplasms. NET: neuroendocrine tumors. RNA-seq: RNA-sequencing. WGS: whole-genome sequencing. RF: random forest.

Ethical Approval

This study was approved by the medical ethical committee of of each respective hospital of the patients: Verenigde Commissies Mensgebonden Onderzoek of the St. Antonius Hospital Nieuwegein, Z-12.55; UMC Utrecht, METC 12-093 HUB-Cancer; NKI Institutional Review Board (IRB), M18ORG/CFMPB582; Maastricht University Medical Center, METC 2019-1061, and 2019-1039.

Consent for publication

All patients signed informed consent forms for molecular analyses and to the publishing of the data.

Competing Interests

Where authors are identified as personnel of the International Agency for Research on Cancer/World Health Organisation, the authors alone are responsible for the views expressed in this article and they do not necessarily represent the decisions, policy or views of the International Agency for Research on Cancer/World Health Organisation.

H.C.'s full disclosure is given at [70]. H.C. is inventor of several patents related to organoid technology, cofounder of Xilis Inc. and currently an employee of Roche, Basel.

Funding

The study was funded by the NET Research Foundation (2017 Petersen Accelerator Award to H.C.), Worldwide Cancer Research (2020 Grant Round to L.F-C), NET Research Foundation (2019 Investigator Award to L.F-C), French National Cancer Institute (INCa, PRT-K 2017 to L.F-C. and M.F.), and Ligue Nationale contre le Cancer (fellowship to L.Ma.). T.L.D. was supported by an EMBO long-term fellowship (ALTF-21-2017) and a Marie Skłodowska-Curie IF grant 797966 – PNECtumor. The Oncode Institute is supported by the Dutch Cancer Society.

Author's Contributions

TD designed the study and conducted the experiments. NA and MF designed the bioinformatic workflows. NA performed the data processing. NA and LM performed the analyses. CV formatted and deposited the data on EGA. MF, LFC, and TD supervised the analyses. NA, TD, AS-O, MF, and LFC wrote the manuscript. All authors reviewed and commented the paper.

Acknowledgements

We thank the patients for participating to the study. We thank Utrecht Sequencing for RNA-sequencing services. We thank the editor and the reviewers for their useful suggestions. The results shown here are in part based upon data generated by the Rare Cancers Genomics initiative (www.rarecancersgenomics.com).

References

1. Clevers H. Modeling development and disease with organoids. Cell 2016;165(7):1586–1597.
2. Kim J, Koo BK, Knoblich JA. Human organoids: model systems for human biology and medicine. Nature Reviews Molecular Cell Biology 2020;21(10):571–584.
3. Drost J, Clevers H. Organoids in cancer research. Nature Reviews Cancer 2018;18(7):407.
4. Tuveson D, Clevers H. Cancer modeling meets human organoid technology. Science 2019;364(6444):952–955.
5. LeSavage BL, Suhar RA, Broguiere N, Lutolf MP, Heilshorn SC. Next-generation cancer organoids. Nature materials 2022;21(2):143–159.
6. Dayton TL, Alcala N, Moonen L, den Hartigh L, Geurts V, Mangiante L, et al. Druggable growth dependencies and tumor evolution analysis in patient-derived organoids of neuroendocrine neoplasms from multiple body sites. Cancer Cell 2023;41(12):2083–2099.e9. https://www.sciencedirect.com/science/article/pii/S1535610823003987.
7. Rindi G, Klimstra DS, Abedi-Ardekani B, Asa SL, Bosman FT, Brambilla E, et al. A common classification framework for neuroendocrine neoplasms: an International Agency for Research on Cancer (IARC) and World Health Organization (WHO) expert consensus proposal. Modern Pathology 2018;31(12):1770–1786.
8. Travis W, Beasley M, Cree I, Papotti M, Rekhtman N, et al. Lung neuroendocrine neoplasms. In: WHO Classification of Tumours Editorial Board IARC Press Lyon; 2022.p. 109–111.
9. Klimstra D, Klöppel G, La Rosa S, Rindi G. Classification of neuroendocrine neoplasms of the digestive system. WHO Classification of tumours, 5th Edition Digestive system tumours 2019;p. 16–19.
10. Rudin CM, Poirier JT, Byers LA, Dive C, Dowlati A, George J, et al. Molecular subtypes of small cell lung cancer: a synthesis of human and mouse model data. Nature Reviews Cancer 2019;19(5):289–297.
11. Derks JL, Leblay N, Lantuejoul S, Dingemans AMC, Speel EJM, Fernandez-Cuesta L. New insights into the molecular characteristics of pulmonary carcinoids and large cell neuroendocrine carcinomas, and the impact on their clinical management. Journal of Thoracic Oncology 2018;13(6):752–766.
12. Fernandez-Cuesta L, Foll M. Molecular studies of lung neuroendocrine neoplasms uncover new concepts and entities. Translational Lung Cancer Research 2019;8(Suppl 4):S430.
13. Zhao Z, Chen X, Dowbaj AM, Sljukic A, Bratlie K, Lin L, et al. Organoids. Nature Reviews Methods Primers 2022;2(1):94.
14. IARC bioinformatics platform github repository; Accessed on 8 February 2024. https://github.com/IARCbioinfo/.
15. Alcala N, Leblay N, Gabriel A, Mangiante L, Hervas D, Giffon T, et al. Integrative and comparative genomic analyses identify clinically relevant pulmonary carcinoid groups and unveil the supra-carcinoids. Nature communications 2019;10.
16. Gabriel AA, Mathian E, Mangiante L, Voegele C, Cahais V, Ghantous A, et al. A molecular map of lung neuroendocrine neoplasms. GigaScience 2020;9(11):giaa112.
17. Di Tommaso P, Chatzou M, Floden EW, Barja PP, Palumbo E, Notredame C. Nextflow enables reproducible computational workflows. Nature biotechnology 2017;35(4):316.
18. Dockerhub Home page; Accessed on 8 February 2024. https://hub.docker.com/.
19. Singularity hub Home page; Accessed on 8 February 2024. https://singularity-hub.org/.
20. IARCbioinfo whole-genome sequencing alignment pipeline; Accessed on 8 February 2024. https://github.com/IARCbioinfo/alignment-nf.
21. Li H, Durbin R. Fast and accurate long-read alignment with Burrows–Wheeler transform. Bioinformatics 2010;26(5):589–595.
22. Vasimuddin M, Misra S, Li H, Aluru S. Efficient architecture-aware acceleration of bwa-mem for multicore systems. In: 2019 IEEE International Parallel and Distributed Processing Symposium (IPDPS) IEEE; 2019. p. 314–324.
23. Faust GG, Hall IM. SAMBLASTER: fast duplicate marking and structural variant read extraction. Bioinformatics 2014;30(17):2503–2505.
24. Tarasov A, Vilella AJ, Cuppen E, Nijman IJ, Prins P. Sambamba: fast processing of NGS alignment formats. Bioinformatics

2015;31(12):2032–2034.
25. IARCbioinfo RNA sequencing alignment pipeline; Accessed on 8 February 2024. https://github.com/IARCbioinfo/RNAseq-nf.
26. Krueger F. Trim Galore: a wrapper tool around Cutadapt and FastQC to consistently apply quality and adapter trimming to FastQ files, with some extra functionality for MspI-digested RRBS-type (Reduced Representation Bisufite-Seq) libraries. URL http://www bioinformatics babraham ac uk/projects/trim_galore/(Date of access: 28/06/2019) 2012;.
27. Martin M. Cutadapt removes adapter sequences from high-throughput sequencing reads. EMBnet journal 2011;17(1):10–12.
28. Dobin A, Davis CA, Schlesinger F, Drenkow J, Zaleski C, Jha S, et al. STAR: ultrafast universal RNA-seq aligner. Bioinformatics 2013;29(1):15–21.
29. IARCbioinfo local re-alignment pipeline; Accessed on 8 February 2024. https://github.com/IARCbioinfo/abra-nf.
30. Mose LE, Wilkerson MD, Hayes DN, Perou CM, Parker JS. ABRA: improved coding indel detection via assembly-based realignment. Bioinformatics 2014;30(19):2813–2815.
31. IARCbioinfo base quality score recalibration pipeline; Accessed on 8 February 2024. https://github.com/IARCbioinfo/BQSR-nf.
32. Van der Auwera GA, Carneiro MO, Hartl C, Poplin R, Del Angel G, Levy-Moonshine A, et al. From FastQ data to high-confidence variant calls: the genome analysis toolkit best practices pipeline. Current protocols in bioinformatics 2013;43(1):11–10.
33. Benjamin D, Sato T, Cibulskis K, Getz G, Stewart C, Lichtenstein L. Calling somatic SNVs and indels with Mutect2. BioRxiv 2019;p. 861054.
34. Van der Auwera GA, O'Connor BD. Genomics in the cloud: using Docker, GATK, and WDL in Terra. O'Reilly Media; 2020.
35. IARCbioinfo GATK mutect2 variant calling pipeline; Accessed on 8 February 2024. https://github.com/IARCbioinfo/mutect-nf.
36. Danecek P, Bonfield JK, Liddle J, Marshall J, Ohan V, Pollard MO, et al. Twelve years of SAMtools and BCFtools. Gigascience 2021;10(2):giab008.
37. IARCbioinfo variant calling format files normalization pipeline; Accessed on 8 February 2024. https://github.com/IARCbioinfo/vcf_normalization-nf.
38. IARCbioinfo variant calling format files annotation with ANNOVAR pipeline; Accessed on 8 February 2024. https://github.com/IARCbioinfo/table_annovar-nf.
39. Kim S, Scheffler K, Halpern AL, Bekritsky MA, Noh E, Källberg M, et al. Strelka2: fast and accurate calling of germline and somatic variants. Nature methods 2018;15(8):591–594.
40. IARCbioinfo strelka2 variant calling pipeline; Accessed on 8 February 2024. https://github.com/IARCbioinfo/strelka2-nf.
41. Andrews S, Krueger F, Segonds-Pichon A, Biggins L, Krueger C, Wingett S, FastQC. Babraham, UK; 2012. Babraham Institute.
42. Ewels P, Magnusson M, Lundin S, Käller M. MultiQC: summarize analysis results for multiple tools and samples in a single report. Bioinformatics 2016;32(19):3047. +http://dx.doi.org/10.1093/bioinformatics/btw354.
43. Okonechnikov K, Conesa A, García-Alcalde F. Qualimap 2: advanced multi-sample quality control for high-throughput sequencing data. Bioinformatics 2015;32(2):292–294.
44. Wang L, Wang S, Li W. RSeQC: quality control of RNA-seq experiments. Bioinformatics 2012;28(16):2184–2185.
45. IARCbioinfo NGSCheckMate sample matching pipeline; Accessed on 8 February 2024. https://github.com/parklab/NGSCheckMate.
46. Love MI, Huber W, Anders S. Moderated estimation of fold change and dispersion for RNA-seq data with DESeq2. Genome biology 2014;15(12):550.
47. Breiman L. Random forests. Machine learning 2001;45:5–32.
48. Liaw A, Wiener M, et al. Classification and regression by randomForest. R news 2002;2(3):18–22.
49. Di Genova A, Mangiante L, Sexton-Oates A, Voegele C, Fernandez-Cuesta L, Alcala N, et al. A molecular phenotypic map of malignant pleural mesothelioma. GigaScience 2023;12:giac128.
50. Ioannidis NM, Rothstein JH, Pejaver V, Middha S, McDonnell SK, Baheti S, et al. REVEL: an ensemble method for predicting the pathogenicity of rare missense variants. The American Journal of Human Genetics 2016;99(4):877–885.
51. IARCbioinfo NGSCheckMate sample matching pipeline; Accessed on 8 February 2024. https://github.com/IARCbioinfo/MS_panNEN_organoids.
52. Ishwaran H, Kogalur UB, Gorodeski EZ, Minn AJ, Lauer MS. High-dimensional variable selection for survival data. Journal of the American Statistical Association 2010;105(489):205–217.
53. Banerjee M, Ding Y, Noone AM. Identifying representative trees from ensembles. Statistics in medicine 2012;31(15):1601–1616.
54. Banck MS, Kanwar R, Kulkarni AA, Boora GK, Metge F, Kipp BR, et al. The genomic landscape of small intestine neuroendocrine tumors. The Journal of clinical investigation 2013;123(6):2502–2508.
55. Sei Y, Zhao X, Forbes J, Szymczak S, Li Q, Trivedi A, et al. A hereditary form of small intestinal carcinoid associated with a germline mutation in inositol polyphosphate multikinase. Gastroenterology 2015;149(1):67–78.
56. Miyoshi T, Umemura S, Matsumura Y, Mimaki S, Tada S, Makinoshima H, et al. Genomic profiling of large-cell neuroendocrine carcinoma of the lung. Clinical Cancer Research 2017;23(3):757–765.
57. Pelosi G, Bianchi F, Dama E, Simbolo M, Mafficini A, Sonzogni A, et al. Most high-grade neuroendocrine tumours of the lung are likely to secondarily develop from pre-existing carcinoids: innovative findings skipping the current pathogenesis paradigm. Virchows Archiv 2018;472:567–577.
58. Simbolo M, Vicentini C, Mafficini A, Fassan M, Pedron S, Corbo V, et al. Mutational and copy number asset of primary sporadic neuroendocrine tumors of the small intestine. Virchows Archiv 2018;473:709–717.
59. Walter D, Harter PN, Battke F, Winkelmann R, Schneider M, Holzer K, et al. Genetic heterogeneity of primary lesion and metastasis in small intestine neuroendocrine tumors. Scientific reports 2018;8(1):3811.
60. Samsom KG, Levy S, van Veenendaal LM, Roepman P, Kodach LL, Steeghs N, et al. Driver mutations occur frequently in metastases of well-differentiated small intestine neuroendocrine tumours. Histopathology 2021;78(4):556–566.
61. Fernandez-Cuesta L, Peifer M, Lu X, Sun R, Ozretić L, Seidel D, et al. Frequent mutations in chromatin-remodelling genes in pulmonary carcinoids. Nature communications 2014;5(1):3518.
62. Mangiante L, Alcala N, Sexton-Oates A, Di Genova A, Gonzalez-Perez A, Khandekar A, et al. Multiomic analysis of malignant pleural mesothelioma identifies molecular axes and specialized tumor profiles driving intertumor heterogeneity. Nature Genetics 2023;55(4):607–618.
63. Lee SH, Hu W, Matulay JT, Silva MV, Owczarek TB, Kim K, et al. Tumor evolution and drug response in patient-derived organoid models of bladder cancer. Cell 2018;173(2):515–528.
64. Laddha SV, Da Silva EM, Robzyk K, Untch BR, Ke H, Rekhtman N, et al. Integrative Genomic Characterization Identifies Molecular Subtypes of Lung CarcinoidsGenomic Analysis Identifies Subtypes of Lung Carcinoids. Cancer research 2019;79(17):4339–4347.
65. Alvarez MJ, Subramaniam PS, Tang LH, Grunn A, Aburi M, Rieckhof G, et al. A precision oncology approach to the pharmacological targeting of mechanistic dependencies in neuroendocrine tumors. Nature genetics 2018;50(7):979–989.

66. Hofving T, Liang F, Karlsson J, Yrlid U, Nilsson JA, Nilsson O, et al. The Microenvironment of Small Intestinal Neuroendocrine Tumours Contains Lymphocytes Capable of Recognition and Activation after Expansion. Cancers 2021;13(17):4305.
67. EGA python client for data download home page; Accessed on 8 February 2024. https://github.com/EGA-archive/ega-download-client.
68. Video tutorial for the EGA python client; Accessed on 8 February 2024. https://embl-ebi.cloud.panopto.eu/Panopto/Pages/Viewer.aspx?id=be79bb93-1737-4f95-b80f-ab4300aa6f5a.
69. Alcala N, Voegele C, Mangiante L, Sexton-Oates A, Clevers H, Fernandez-Cuesta L, et al. Supporting data for "Multi-omic dataset of patient-derived tumor organoids of neuroendocrine neoplasms". GigaScience Database 2024;http://dx.doi.org/10.5524/102494.
70. Pr. Hans Clevers competing interest disclosure; Accessed on 8 February 2024. https://www.uu.nl/staff/JCClevers/.

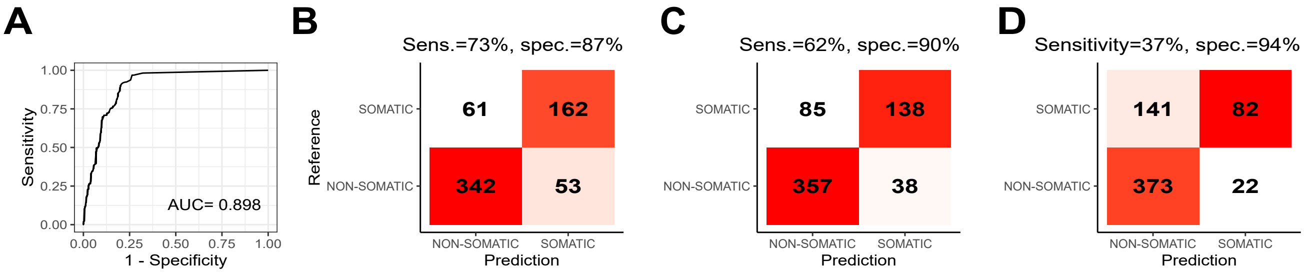

Figure S1. Random forest (RF) classification of variants in genes not reported as driver in neuroendocrine neoplasms. (A) Receiver operating characteristic (ROC) curve. (E)-(G) Confusion matrix for different levels of sensitivity and specificity. Reference: somatic status assessed from whole-genome sequencing data. Prediction: somatic status predicted from RNA-seq data using the RF algorithm.

Click here to access/download
Supplementary Material
SupplementaryFileS1.html

Supplementary File S2

Click here to access/download
Supplementary Material
SupplementaryFileS2.html

Supplementary File S3

Click here to access/download
Supplementary Material
SupplementaryFileS3.html

Supplementary File S4

Click here to access/download
Supplementary Material
SupplementaryFileS4.html
