# Supplementary material for: Multi-omic dataset of patient-derived tumor organoids of neuroendocrine neoplasms
Source: Gigascience. 2024 Mar 7;13:giae008. doi: 10.1093/gigascience/giae008 (PMC10919335; doi:10.1093/gigascience/giae008)
Supplement: giae008_Response_to_Reviewer_Comments_Original_Submission [file giae008_response_to_reviewer_comments_original_submission.pdf]

Author's Response To Reviewer Comments

Dear Dr Alcala,

Your manuscript "Multi-omic dataset of patient-derived tumor organoids of neuroendocrine neoplasms" (GIGA-D-23-00277) has been assessed by our reviewers. Based on these reports, and my own assessment as Editor, I am pleased to inform you that it is potentially acceptable for publication in GigaScience, once you have carried out some essential revisions suggested by our reviewers. In particular, Reviewer #3 would like the detailed data processing steps to be added to the GitHub page, for ease of reuse and reproducibility.
Answer: Dear Dr Nogoy, thank you very much for your positive feedback and for the possibility to submit a revised version. Please find a detailed answer to all comments below. In particular, we have added the requested details about data processing on the github page, now providing the exact command lines used for each data processing step as well as all the parameters used. Also note that we opted at submission to also submit to GigaDB all H&E section images and Immunohistochemistry section images of markers we generated, so we now also report them in this revised version of the manuscript. In the revised manuscript, we highlight in red important modifications.

Reviewer reports
Reviewer #1

In this manuscript, Alcala et al. have reported on the whole genome sequencing (WGS) and RNA sequencing (RNA-seq) of 23 patient-derived tumor organoids of neuroendocrine neoplasms. This is a detailed report on the quality control of WGS, RNA-seq, and sample swap. The methods are solid and well-described. The raw sequencing data have been deposited in a public repository. This dataset could be a valuable resource for exploring the biology and treatment of this rare type of tumor.
Answer: We thank the reviewer for their positive assessment of our work.

Here are my comments to the authors:
Comment 1. Could you please clarify whether the organoids described in this manuscript will be distributed? If so, could you provide the contact address and any restrictions, such as a material transfer agreement?
Answer: We thank the reviewer for pointing out this oversight. We now mention the contact address and procedure to obtain the organoids p. 10: “Organoid lines mentioned in this manuscript can be requested from Hans Clevers (h.clevers@hubrecht.eu) or Talya Dayton (talya.dayton@embl.es). Distribution of organoids to third parties will have to be authorized by the relevant ethical committee and a complete material transfer agreement will be required to ensure compliance with the Dutch ‘medical research involving human subjects’ act. Use of organoids is subjected to patient consent; note that upon consent withdrawal, distributed organoid lines and any derived material will have to be promptly disposed of.”

Comment 2. You have deposited the RNA-seq gene expression matrix in the public repository European Genome-phenome Archive (dataset ID: EGAD00001009994). However, the file is under controlled access. This limits the availability of data, especially for scientists who just want a quick glance at the data. Since the gene expression matrix does not contain personally identifiable information, I wonder if you could make the file open access. You have reported how you detected somatic mutations in the organoids. However, you did not share the list of detected mutations. Sharing this list would help scientists who do not have a computational background. Open access is preferable in this case, but controlled access is also acceptable because germline variants could be misclassified as somatic.
Answer: The reviewer makes a valid point; we now provide the gene expression matrix and the small variant alterations in the public github repository associated with the manuscript (https://github.com/IARCbioinfo/MS_panNEN_organoids) and provide a permanent link to their location p. 10.

Comment 3. The primary site of mLCNEC23 is unknown. Could you infer its primary site based on gene expression patterns or driver mutations?
Answer: We now mention p. 8 that molecular data of mLCNEC23 did support its LCNEC nature---clustering with other LCNEC and presence of mutations characteristic of LCNEC such as in gene TP53---but that since LCNEC from multiple organs clustered together and had similar driver mutations, the organ of origin could not be determined based on molecular data.

Comment 4. I have concerns about the generalizability of your random forest model because it was trained using only 22 somatic mutations. Could you assess your prediction model using publicly available datasets of cancer genomes (e.g., TCGA)?
Answer: We now provide additional elements to support the generalization of our model pp. 7-8. We show in a new Figure S1 that the random forest model trained on these 22 somatic mutations from known neuroendocrine neoplasm cancer genes produces similar accuracy when tested on the 223 somatic and 395 non-somatic mutations from all other recurrently mutated genes from the cohort (AUC=0.90, sensitivity up to 73% with a specificity above 87%). We also mention that we have used a similar approach in the past and validated it on WGS tumor-only samples, which allowed to "classify variants called from tumor-only WGS data as somatic or germline with high performance (accuracy greater than 92%; di Genova et al. GigaScience 2023)".

Reviewer #2

Alcala et al., did an excellent work on rare cancer type by creating PDTOs molecular fingerprint which has a direct impact for researcher working on these rare cancer type. As a data note, this is excellent resource and covering huge gap in this rare cancer field.

These PDTOs holds high impact specially for such cancers which are slow growing and not easy culture in lab. Authors covered details regarding each technique used in this study and figures are clear to understand with exceptional writing.
Answer: We thank the reviewer for their positive assessment of our work.

Minor comments:
Comment 1. Did authors compare the PDTOs to tumor molecular dataset ? This will be the key to understand how closely and qualitatively PTDOs are related to actual tumor datasets molecular profile. It is not clear in the current version and it will be helpful to readers to decide whether PTDOs molecular fingerprint system are valuable to them. This is not required for this manuscript to address but a note will be helpful to make valulabe decision to use such resources and with what limitations.
Answer: We agree with the reviewer that although this is beyond the scope of such a data note paper, the comparison of PDTOs and parental tumors is key. Thus, we have added on p. 8 a summary of the results from the associated research article (Dayton, Alcala et al. In press), where this comparison is thoroughly investigated. In addition, in this data note we report all the scripts used to perform the thorough comparison between PDTOs and parental tumors presented in the associated research article. In particular, we mention the location of the scripts showing that the organoids faithfully represent the gene expression profile and genomic profile of their parental tumors (freely available at https://github.com/IARCbioinfo/MS_panNEN_organoids/tree/main/Rscripts).

Comment 2. Authors covered longitudinal samples in this system for 1 to 2 timepoints. What changes did they observe (molecularly) looking at this data from a longitudinal timepoints view will be helpful for readers. Also, based on author's experience for longitudinal sampling, do authors have key suggestions for researcher ? a brief discussion will be helpful.
Answer: This is also a key aspect covered in the associated research article. We now summarize in this data note p. 8 the main results from the associated research article, in particular mentioning that " PDTOs preserve the genetic diversity and clonal architecture of their parents across long periods of time (6 months to more than a year)". We also report all the scripts for the longitudinal evolutionary analyses of the samples, so researchers can use it as inspiration for their own studies.

Comment 3. Authors did comprehensive small variant analysis from WGS and RNAseq. Did you authors find

known somatic variations for these samples ? mainly comparing against the known published mutational landscape. A note of this will be helpful.
Answer: We now mention p. 8 that “both variants identified with WGS and variants identified with RNA-seq include driver mutations in key recurrently altered LCNEC driver genes such as TP53 (mutated in 5/5 LCNEC) and STK11 (mutated in 3/5 LCNEC). We also identified mutations or structural variants in known driver genes in all but one neuroendocrine tumors (17/18), but as previously reported, they involve multiple genes instead of recurrently mutated genes (Fernandez-Cuesta et al. 2014). This confirms that PDTOs recapitulate the genomic profile of neuroendocrine neoplasms”. We also now mention that we report all the scripts necessary to perform these analyses p. 8.

Comment 4. A comment on limitations of PTDOs and molecular fingerprint created from such PDTOs will be valuable.
Answer: We now mention in the re-use potential section p. 9 the limitations of this PDTO biobank, notably “that the slow passage time of low-grade PDTOs makes them appropriate models to study the biology of neuroendocrine tumors, but challenges their use for drug testing. This is particularly true of small intestine NETs, which were only short term cultures that did not grow past four passage. Finally, as noted in most molecular studies of PDTOs (Lee et al Cell 2018), one of the main differences between PDTOs and their parental tumors is the absence of microenvironment. Future work would ideally focus on creating co-cultures of PDTOs and immune cells to remedy this shortcoming”.

Comment 5. Authors briefly comment on using such molecular datasets from PDTOs and combining with other datasets to improve on power statistics to discover informative molecular features of these cancers. This points towards my first point on how similar PDTOs are to tumor molecular profile.
Answer: We now provide in the github repository the expression matrix processed exactly as our past (Gabriel et al. Gigascience 2020) and future studies (Sexton-Oates et al. In prep), and mention its public location in the re-use potential section. We refer the reviewer to the answer to comment 1 for details about similarities with reference tumors.

Reviewer #3

The authors conducted a study where they generated multi-omics datasets, including whole-genome sequencing and RNA sequencing , for rare neuroendocrine tumors in the lungs, small intestine, and large cells. They used patient-derived tumor organoids and performed quality control analysis on the datasets. Additionally, they developed a random forest classifier specifically for detecting mutations in the RNA-seq data. The pipeline used in this study is well-organized, but I have a few queries that I would like to clarify before recommending it for publication.

Major concerns:
Comment 1. The data processing and quality control procedures would be valuable for other researchers working with similar datasets. It would be beneficial to add these procedures to the GitHub repository (https://github.com/IARCbioinfo/MS_panNEN_organoids). Furthermore, it would be helpful to provide insights into what constitutes good quality reads, such as the number of unique reads and the ratio of duplicate reads.
Answer: We thank the reviewer for noting this oversight in our code availability. We have now added all the command lines used for data processing the RNA-seq and WGS in the github repository (see readme https://github.com/IARCbioinfo/MS_panNEN_organoids) and mention them in the code availability section p. 10. Note that we also provide the processed data per the other reviewers' suggestions (gene expression and small variants, see answer to comment 2 from reviewer 1). Finally, we now also mention the general guidelines from software fastQC regarding the quality of reads p. 4.

Comment 2. Regarding the random forest (RF) model, it is mentioned that there are 10 features. Could you clarify if these features are from the public information, or are all the features extracted solely from the RNA-seq data? Also, does the RF model work for WGS data as well? Was there any specific design implemented to address the issue of imbalanced positive and negative samples?
Answer: We now explicitly mention p. 6 which of the 10 features come from the RNA-seq data (4/10 features) and which are annotations coming from public databases (6/10 features). We also mention p. 7

that indeed a similar model was used in Di Genova et al. on WGS data to classify variants from tumor-only samples. Regarding the issue of imbalanced positive and negative samples, we now mention pp. 6-7 that we preferred to keep this imbalance in the training set to force the algorithm to take into account the fact that most variants are not somatic, and thus having a very good specificity is key to avoid large false discovery rates.

Comment 3. RNA-seq are not used to generate the gene expression here, which would waste important information.
Answer: We now make it explicit that we did generate gene expression data and that it is available on the github and EGA repositories (see revised data availability section p. 9).

Minor concerns:
Comment 4. In Figure 6C, what does "Mean minimum depth" refer to?
Answer: We now describe in the figure legend that “Mean minimum depth” refers to the “tree depth (1: root, value $>>1$: leaves) of the first time the feature is used for classification, averaged across all trees; low values indicate features often used at the root and thus particularly important”.

Comment 5. Is the most important feature identified by the RF model a good predictor?
Answer: We now mention p. 8 that while this feature is particularly important, “using the most important feature alone (the REVEL score) led to a much lower accuracy, consistent with the importance of other features such as TLOD and pathogenic annotations (COSMIC, InterVar)”.
