# Supplementary material for: Multi-omic dataset of patient-derived tumor organoids of neuroendocrine neoplasms
Source: Gigascience. 2024 Mar 7;13:giae008. doi: 10.1093/gigascience/giae008 (PMC10919335; doi:10.1093/gigascience/giae008)
Supplement: giae008_Reviewer_1_Report_Original_Submission — Masashi Fujita -- 10/29/2023 [file giae008_reviewer_1_report_original_submission.pdf]

Reviewer Report

Title: Multi-omic dataset of patient-derived tumor organoids of neuroendocrine neoplasms

Version: Original Submission **Date:** 10/29/2023

Reviewer name: Masashi Fujita

Reviewer Comments to Author:

In this manuscript, Alcala et al. have reported on the whole genome sequencing (WGS) and RNA sequencing (RNA-seq) of 23 patient-derived tumor organoids of neuroendocrine neoplasms. This is a detailed report on the quality control of WGS, RNA-seq, and sample swap. The methods are solid and well-described. The raw sequencing data have been deposited in a public repository. This dataset could be a valuable resource for exploring the biology and treatment of this rare type of tumor.Here are my comments to the authors:Could you please clarify whether the organoids described in this manuscript will be distributed? If so, could you provide the contact address and any restrictions, such as a material transfer agreement?You have deposited the RNA-seq gene expression matrix in the public repository European Genome-phenome Archive (dataset ID: EGAD00001009994). However, the file is under controlled access. This limits the availability of data, especially for scientists who just want a quick glance at the data. Since the gene expression matrix does not contain personally identifiable information, I wonder if you could make the file open access.You have reported how you detected somatic mutations in the organoids. However, you did not share the list of detected mutations. Sharing this list would help scientists who do not have a computational background. Open access is preferable in this case, but controlled access is also acceptable because germline variants could be misclassified as somatic.The primary site of mLCNEC23 is unknown. Could you infer its primary site based on gene expression patterns or driver mutations?I have concerns about the generalizability of your random forest model because it was trained using only 22 somatic mutations. Could you assess your prediction model using publicly available datasets of cancer genomes (e.g., TCGA)?

Level of Interest

Please indicate how interesting you found the manuscript: Choose an item.

Quality of Written English

Please indicate the quality of language in the manuscript: Choose an item.

Declaration of Competing Interests

Please complete a declaration of competing interests, considering the following questions:

- Have you in the past five years received reimbursements, fees, funding, or salary from an organisation that may in any way gain or lose financially from the publication of this manuscript, either now or in the future?
- Do you hold any stocks or shares in an organisation that may in any way gain or lose financially from the publication of this manuscript, either now or in the future?
- Do you hold or are you currently applying for any patents relating to the content of the manuscript?
- Have you received reimbursements, fees, funding, or salary from an organization that holds or has applied for patents relating to the content of the manuscript?
- Do you have any other financial competing interests?
- Do you have any non-financial competing interests in relation to this paper?

If you can answer no to all of the above, write 'I declare that I have no competing interests' below. If your reply is yes to any, please give details below.

I declare that I have no competing interests.

I agree to the open peer review policy of the journal. I understand that my name will be included on my report to the authors and, if the manuscript is accepted for publication, my named report including any attachments I upload will be posted on the website along with the authors' responses. I agree for my report to be made available under an Open Access Creative Commons CC-BY license (http://creativecommons.org/licenses/by/4.0/). I understand that any comments which I do not wish to be included in my named report can be included as confidential comments to the editors, which will not be published.

Choose an item.

To further support our reviewers, we have joined with Publons, where you can gain additional credit to further highlight your hard work (see: https://publons.com/journal/530/gigascience). On publication of this paper, your review will be automatically added to Publons, you can then choose whether or not to claim your Publons credit. I understand this statement.

Yes Choose an item.
