# Supplementary material for: Multi-omic dataset of patient-derived tumor organoids of neuroendocrine neoplasms
Source: Gigascience. 2024 Mar 7;13:giae008. doi: 10.1093/gigascience/giae008 (PMC10919335; doi:10.1093/gigascience/giae008)
Supplement: giae008_Reviewer_2_Report_Original_Submission — Saurabh V Laddha -- 11/9/2023 [file giae008_reviewer_2_report_original_submission.pdf]

Reviewer Report

Title: Multi-omic dataset of patient-derived tumor organoids of neuroendocrine neoplasms

Version: Original Submission **Date:** 11/9/2023

Reviewer name: Saurabh V Laddha

Reviewer Comments to Author:

Alcala et al., did an excellent work on rare cancer type by creating PDTOs molecular fingerprint which has a direct impact for researcher working on these rare cancer type. As a data note, this is excellent resource and covering huge gap in this rare cancer field.These PDTOs holds high impact specially for such cancers which are slow growing and not easy culture in lab. Authors covered details regarding each technique used in this study and figures are clear to understand with exceptional writing.Minor comments:- Did authors compare the PDTOs to tumor molecular dataset ? This will be the key to understand how closely and qualitatively PTDOs are related to actual tumor datasets molecular profile. It is not clear in the current version and it will be helpful to readers to decide whether PTDOs molecular fingerprint system are valuable to them. This is not required for this manuscript to address but a note will be helpful to make valulabe decision to use such resources and with what limitations.- Authors covered longitudinal samples in this system for 1 to 2 timepoints. What changes did they observe (molecularly) looking at this data from a longitudinal timepoints view will be helpful for readers. Also, based on author's experience for longitudinal sampling, do authors have key suggestions for researcher ? a brief discussion will be helpful.- Authors did comprehensive small variant analysis from WGS and RNAseq. Did you authors find known somatic variations for these samples ? mainly comparing against the known published mutational landscape. A note of this will be helpful.- A comment on limitations of PTDOs and molecular fingerprint created from such PDTOs will be valuable.- Authors briefly comment on using such molecular datasets from PDTOs and combining with other datasets to improve on power statistics to discover informative molecular features of these cancers. This points towards my first point on how similar PDTOs are to tumor molecular profile.

Level of Interest

Please indicate how interesting you found the manuscript: Choose an item.

Quality of Written English

Please indicate the quality of language in the manuscript: Choose an item.

Declaration of Competing Interests

Please complete a declaration of competing interests, considering the following questions:

- Have you in the past five years received reimbursements, fees, funding, or salary from an organisation that may in any way gain or lose financially from the publication of this manuscript, either now or in the future?
- Do you hold any stocks or shares in an organisation that may in any way gain or lose financially from the publication of this manuscript, either now or in the future?
- Do you hold or are you currently applying for any patents relating to the content of the manuscript?
- Have you received reimbursements, fees, funding, or salary from an organization that holds or has applied for patents relating to the content of the manuscript?
- Do you have any other financial competing interests?
- Do you have any non-financial competing interests in relation to this paper?

If you can answer no to all of the above, write 'I declare that I have no competing interests' below. If your reply is yes to any, please give details below.

I declare that I have no competing interests

I agree to the open peer review policy of the journal. I understand that my name will be included on my report to the authors and, if the manuscript is accepted for publication, my named report including any attachments I upload will be posted on the website along with the authors' responses. I agree for my report to be made available under an Open Access Creative Commons CC-BY license (http://creativecommons.org/licenses/by/4.0/). I understand that any comments which I do not wish to be included in my named report can be included as confidential comments to the editors, which will not be published.

Choose an item.

To further support our reviewers, we have joined with Publons, where you can gain additional credit to further highlight your hard work (see: https://publons.com/journal/530/gigascience). On publication of this paper, your review will be automatically added to Publons, you can then choose whether or not to claim your Publons credit. I understand this statement.

Yes Choose an item.
