# Supplementary material for: Multi-omic dataset of patient-derived tumor organoids of neuroendocrine neoplasms
Source: Gigascience. 2024 Mar 7;13:giae008. doi: 10.1093/gigascience/giae008 (PMC10919335; doi:10.1093/gigascience/giae008)
Supplement: giae008_Reviewer_3_Report_Original_Submission — Qiuyue Yuan -- 11/15/2023 [file giae008_reviewer_3_report_original_submission.pdf]

Reviewer Report

Title: Multi-omic dataset of patient-derived tumor organoids of neuroendocrine neoplasms

Version: Original Submission **Date:** 11/15/2023

Reviewer name: Qiuyue Yuan

Reviewer Comments to Author:

The authors conducted a study where they generated multi-omics datasets, including whole-genome sequencing and RNA sequencing , for rare neuroendocrine tumors in the lungs, small intestine, and large cells. They used patient-derived tumor organoids and performed quality control analysis on the datasets. Additionally, they developed a random forest classifier specifically for detecting mutations in the RNA-seq data. The pipeline used in this study is well-organized, but I have a few queries that I would like to clarify before recommending it for publication.Major concerns:The data processing and quality control procedures would be valuable for other researchers working with similar datasets. It would be beneficial to add these procedures to the GitHub repository (https://github.com/IARCbioinfo/MS_panNEN_organoids). Furthermore, it would be helpful to provide insights into what constitutes good quality reads, such as the number of unique reads and the ratio of duplicate reads.Regarding the random forest (RF) model, it is mentioned that there are 10 features. Could you clarify if these features are from the public information, or are all the features extracted solely from the RNA-seq data? Also, does the RF model work for WGS data as well?Was there any specific design implemented to address the issue of imbalanced positive and negative samples?RNA-seq are not used to generate the gene expression here, which would waste important information.Minor concerns:In Figure 6C, what does "Mean minimum depth" refer to?Is the most important feature identified by the RF model a good predictor?

Level of Interest

Please indicate how interesting you found the manuscript: Choose an item.

Quality of Written English

Please indicate the quality of language in the manuscript: Choose an item.

Declaration of Competing Interests

Please complete a declaration of competing interests, considering the following questions:

- Have you in the past five years received reimbursements, fees, funding, or salary from an organisation that may in any way gain or lose financially from the publication of this manuscript, either now or in the future?

- Do you hold any stocks or shares in an organisation that may in any way gain or lose financially from the publication of this manuscript, either now or in the future?
- Do you hold or are you currently applying for any patents relating to the content of the manuscript?
- Have you received reimbursements, fees, funding, or salary from an organization that holds or has applied for patents relating to the content of the manuscript?
- Do you have any other financial competing interests?
- Do you have any non-financial competing interests in relation to this paper?

If you can answer no to all of the above, write 'I declare that I have no competing interests' below. If your reply is yes to any, please give details below.

I declare that I have no competing interests.

I agree to the open peer review policy of the journal. I understand that my name will be included on my report to the authors and, if the manuscript is accepted for publication, my named report including any attachments I upload will be posted on the website along with the authors' responses. I agree for my report to be made available under an Open Access Creative Commons CC-BY license (http://creativecommons.org/licenses/by/4.0/). I understand that any comments which I do not wish to be included in my named report can be included as confidential comments to the editors, which will not be published.

Choose an item.

To further support our reviewers, we have joined with Publons, where you can gain additional credit to further highlight your hard work (see: https://publons.com/journal/530/gigascience). On publication of this paper, your review will be automatically added to Publons, you can then choose whether or not to claim your Publons credit. I understand this statement.

Yes Choose an item.
