# Supplementary material for: Multi-omic dataset of patient-derived tumor organoids of neuroendocrine neoplasms
Source: Gigascience. 2024 Mar 7;13:giae008. doi: 10.1093/gigascience/giae008 (PMC10919335; doi:10.1093/gigascience/giae008)
Supplement: giae008_Authors_ReResponse_to_Reviewer_Comments_Revision_1sponse_To_Reviewer_Comments [file giae008_authors_reresponse_to_reviewer_comments_revision_1sponse_to_reviewer_comments.pdf]

Author's Response To Reviewer Comments

Dear Editor,

Thank you for the provisional acceptance of our manuscript. Please find attached a revision that follows the journal formatting guidelines.

Best,

Nicolas
